# Supplementary material for: Systematic review of diagnostic and prognostic models of chronic kidney disease in low-income and middle-income countries
Source: BMJ Open. 2022 Mar 15;12(3):e058921. doi: 10.1136/bmjopen-2021-058921 (PMC8928240; doi:10.1136/bmjopen-2021-058921)
Supplement: Author's manuscript [file bmjopen-2021-058921.draft_revisions.pdf]

BMJ Open is committed to open peer review. As part of this commitment we make the peer review history of every article we publish publicly available.

When an article is published we post the peer reviewers' comments and the authors' responses online. We also post the versions of the paper that were used during peer review. These are the versions that the peer review comments apply to.

The versions of the paper that follow are the versions that were submitted during the peer review process. They are not the versions of record or the final published versions. They should not be cited or distributed as the published version of this manuscript.

BMJ Open is an open access journal and the full, final, typeset and author-corrected version of record of the manuscript is available on our site with no access controls, subscription charges or pay-per-view fees (http://bmjopen.bmj.com).

If you have any questions on BMJ Open's open peer review process please email
info.bmjopen@bmj.com

BMJ Open

A systematic review of diagnostic and prognostic models of chronic kidney disease in low- and middle- income countries

Journal:	*BMJ Open*
Manuscript ID	bmjopen-2021-058921
Article Type:	Original research
Date Submitted by the Author:	02-Nov-2021

Complete List of Authors:	Aparcana-Granda, Diego J.; School of Medicine 'Alberto Hurtado', Universidad Peruana Cayetano Heredia, Lima, Peru; CRONICAS Centre of Excellence in Chronic Diseases, Universidad Peruana Cayetano Heredia, Lima, Peru<br>Ascencio, Edson J.; School of Medicine 'Alberto Hurtado', Universidad Peruana Cayetano Heredia, Lima, Peru; Emerge, Emerging Diseases and Climate Change Research Unit, School of Public Health and Administration, Universidad Peruana Cayetano Heredia, Lima, Peru<br>Carrillo Larco, Rodrigo M. ; Imperial College London,

Keywords:	Chronic renal failure < NEPHROLOGY, EPIDEMIOLOGY, PUBLIC HEALTH, NEPHROLOGY

SCHOLARONE™
Manuscripts

I, the Submitting Author has the right to grant and does grant on behalf of all authors of the Work (as defined in the below author licence), an exclusive licence and/or a non-exclusive licence for contributions from authors who are: i) UK Crown employees; ii) where BMJ has agreed a CC-BY licence shall apply, and/or iii) in accordance with the terms applicable for US Federal Government officers or employees acting as part of their official duties; on a worldwide, perpetual, irrevocable, royalty-free basis to BMJ Publishing Group Ltd ("BMJ") its licensees and where the relevant Journal is co-owned by BMJ to the co-owners of the Journal, to publish the Work in this journal and any other BMJ products and to exploit all rights, as set out in our licence.

The Submitting Author accepts and understands that any supply made under these terms is made by BMJ to the Submitting Author unless you are acting as an employee on behalf of your employer or a postgraduate student of an affiliated institution which is paying any applicable article publishing charge ("APC") for Open Access articles. Where the Submitting Author wishes to make the Work available on an Open Access basis (and intends to pay the relevant APC), the terms of reuse of such Open Access shall be governed by a Creative Commons licence – details of these licences and which Creative Commons licence will apply to this Work are set out in our licence referred to above.

Other than as permitted in any relevant BMJ Author's Self Archiving Policies, I confirm this Work has not been accepted for publication elsewhere, is not being considered for publication elsewhere and does not duplicate material already published. I confirm all authors consent to publication of this Work and authorise the granting of this licence.

For peer review only

A systematic review of diagnostic and prognostic models of Chronic kidney disease in Low- and Middle- Income Countries

Diego J. Aparcana-Granda[1,2], Edson J. Ascencio[1,3], Rodrigo M. Carrillo-Larco[2,4]*

[1] School of Medicine 'Alberto Hurtado', Universidad Peruana Cayetano Heredia, Lima, Peru

[2] CRONICAS Centre of Excellence in Chronic Diseases, Universidad Peruana Cayetano Heredia, Lima, Peru

[3] Emerge, Emerging Diseases and Climate Change Research Unit, School of Public Health and Administration, Universidad Peruana Cayetano Heredia, Lima, Peru

[4] Department of Epidemiology and Biostatistics, School of Public Health, Imperial College London, London, UK

*Corresponding author (RMCL)

Email: rcarrill@ic.ac.uk

For peer review only

For peer review only - http://bmjopen.bmj.com/site/about/guidelines.xhtml

ABSTRACT

Objective: To summarize available chronic kidney disease (CKD) diagnostic and prognostic models in Low- and Middle-Income countries (LMIC)

Method: Systematic review (PRISMA guidelines). We searched Medline, Embase, Global Health, Scopus and Web of Science. We first screened titles and abstracts, and then studied in detail the selected reports; both phases were conducted by two reviewers independently. We followed the CHARMS recommendations and used the PROBAST for risk of bias was assessment.

Results: The search retrieved 14,845 results, 11 reports were studied in detail and nine (n= 61,134) were included in the qualitative analysis. The proportion of women in the study population varied between 24.5%-76.6%, and the mean age ranged between 41.8-57.7 years. Prevalence of undiagnosed chronic kidney disease ranged between 1.1%-29.7%. Age, diabetes mellitus and sex were the most common predictors in the diagnostic and prognostic models. Outcome definition varied greatly, mostly consisting of urinary albumin-to-creatinine ratio and estimated glomerular filtration rate. The highest performance metric was the negative predictive value. All studies exhibited high risk of bias, and some had methodological limitations.

Conclusion: There is no strong evidence to support the use of a CKD diagnostic or prognostic model throughout LMIC. The development, validation and implementation of risk scores must be a research and public health priority in LMIC to enhance CKD screening to improve timely diagnosis.

Keywords: population health; prognosis research; non-communicable diseases

For peer review only

Strengths and limitations of this study

- An extensive search was conducted, involving five major databases (Medline, Embase, Global Health, Scopus and Web of Science).
- A comprehensive list of available CKD diagnostic and prognostic models and their limitations is provided, which were not previously accounted for the LMIC population.
- This study adhered to PRISMA, CHARMS and PROBAST guidelines.
- Meta-analysis was not possible due to the heterogeneity in the measurement of outcomes.
- Additional data sources such as grey literature were not retrieved.

For peer review only

INTRODUCTION

Chronic kidney disease (CKD) is a condition with a large burden globally. Between 1990 and 2017, the health metrics of CKD showed a bleak profile: mortality, incidence and kidney transplantation rates increased by 3%, 29% and 34%, respectively.[1] CKD led to 1.2 million deaths in 2017 and in the best-case scenario, CKD mortality will increase to 2.2 million deaths and become the 5th cause of years of life lost (YLL) by 2040.[2] CKD reveals disparities between low- and middle-income countries (LMIC) and high-income countries (HIC). In the period 1990-2016, the age-standardised disability-adjusted life-years (DALY) due to CKD was the highest in LMIC,[3] where they need to optimize CKD early diagnosis.

Risk scores are a cost-effective alternative for CKD screening and early diagnosis.[4] These equations require less resources and contribute to decision making,[5] and allow screening large populations.[4] Many of the available CKD risk scores have been developed in HIC,[6-8] and they may not be used in LMIC without recalibration to secure accurate predictions. How many CKD risk scores there are for LMIC, and what their strengths and limitations are, remains largely unknown.[9 10] This limits our knowledge of what tools there are to enhance CKD screening in LMIC. Similarly, this lack of evidence prevents planning research to overcome the limitations of available models. To fill these gaps and to inform CKD screening strategies in LMIC, we summarized available CKD diagnostic and prognostic models in LMIC.

For peer review only

METHODS

Protocol and registration

This systematic review and critical appraisal of the scientific literature was conducted following the Preferred Reporting Items for Systematic Reviews and Meta-Analysis guidelines (PRISMA) statement[11] (S1 Table). Protocol is available elsewhere[12] and in the S1 Text. We followed the CHecklist for critical Appraisal and data extraction for systematic Reviews of prediction Modelling Studies (CHARMS) guidelines.[13 14]

Information sources

We searched Medline, EMBASE, Global Health (these three through OVID), Scopus and Web of Science from inception to April 9th, 2021, April 17th, 2021 and April 18th, 2021, respectively. The

For peer review only - http://bmjopen.bmj.com/site/about/guidelines.xhtml

search strategy is available in S2 Table. We also screened the references of relevant systemic reviews[10] and of the selected studies.

Eligibility criteria

We sought diagnostic and prognostic models which assessed the current CKD status (i.e., diagnostic) or future CKD risk (i.e., prognostic), aiming to inform physicians, researchers and the general population (Table 1). Reports could include model derivation, external validation or both. The target population was adults (≥18 years) in LMIC according to The World Bank.[15]

Study selection

Reports were selected if the study population included people who were from and currently living in LMIC. Cross-sectional (diagnostic models) and longitudinal studies (prognostic models) with a random sample of the general population were included. The outcome was CKD based on a laboratory or imaging test (isolated or in combination with self-reported diagnosis): urine albumin-creatinine ratio, urine protein-creatinine ratio, albumin excretion ratio, urine sediment, kidney images, kidney biopsy or the estimated glomerular filtration rate (eGFR).[12]

Reports had to present the development and/or validation of a multivariable model. On the other hand, reports with LMIC populations outside LMIC, or those including foreigners living in LMIC, were excluded. Reports that only studied people with underlying conditions (e.g., patients with diabetes), people with a specific risk factor (e.g., alcohol consumption), or a hospital-based population, were excluded. We also excluded models that were developed using machine learning techniques.

Data collation

We used EndNote20 and Rayyan[16] to remove duplicates from the search results. We used Rayyan[16] to screen titles and abstracts by two reviewers independently (DJA-G and EJA); discrepancies were solved by consensus. Two reviewers independently (DJA-G and EJA) studied the full length of the reports selected in the screening phase; discrepancies were solved by consensus. If consensus was not reached, a third party was consulted (RMC-L). A data extraction form based on the CHARMS guidelines[14] was developed and not modified during data collation. Data was extracted as presented in the original reports by two reviewers independently (DJA-G and EJA); discrepancies were solved by consensus.

For peer review only - http://bmjopen.bmj.com/site/about/guidelines.xhtml

For peer review only

Risk of bias of individual studies

We used the PROBAST (Prediction model Risk of Bias ASsessment Tool) to assess the risk of bias of diagnostic and prognostic models.[17 18] Two reviewers (EJA and DJA-G) independently ascertained the risk of bias of individual reports; discrepancies were solved by consensus or a third party (RMC-L).

Synthesis of results

A qualitative synthesis was conducted whereby the characteristics of the selected models was comprehensively described.[12] Quantitative analysis (meta-analysis) was not conducted because the selected models used different predictors and they had different outcome definitions.

Ethics

This review was deemed as a low risk because human subjects were not directly involved. The funder did not have any role in the conception, conduction, results interpretation, and drafting of this work. Results and opinions expressed in the article are entirely the author's alone responsibility.

RESULTS

Reports selection

The search yielded 14,845 reports. After removing duplicates (1,462 articles), we screened 13,383 titles and abstracts**.** Then, 11 reports were selected, one of them was not available as full-text,[19] and the rest (10 articles) were studied in detail. We excluded one report because the study population was selected by convenience,[20] and another report because it was conducted in a HIC.[21] Additionally, one report was identified by reference searching.[22] Finally, nine reports (n=61,134) were included in the qualitative synthesis (Figure 1).

General characteristics of the selected reports

Original reports were from Iran,[23] India,[24] Peru, [25] South Africa, [22] two from China[26 27] and three from Thailand[28-30] (S1 Figure). All studies were developed on community-based populations with random sampling (S3 Table).

Overall, Wu and colleagues studied the largest sample size (n=14,374) which was a population of workers who underwent health checks;[27] conversely, the smallest sample was studied by Mogueo *et al* (n=902).[22] The oldest data was collected in 1999[23] whereas the most recent one dated from 2018.[23]

For peer review only - http://bmjopen.bmj.com/site/about/guidelines.xhtml

The sample size analysed to derive the diagnostic models ranged from 2,368[25] to 14,374 people,[27] and from 902[22] to 4,940[24] for the validation models. The mean age of participants in the derivation models varied from 44.9 to 57.7 years, and the proportion of male subjects ranged from 46.8% to 70.5%.[24-27 29 30] The mean age of participants in the validation models varied from 41.8 to 57.1 years, and the proportion of male subjects ranged from 23.4% to 75.5%[22-25 27-29] (Table 2; S3 Table).

The number of CKD cases varied greatly in the derivation models, from 81[25] to 947;[24] the corresponding numbers in the validation models were 27[29] and 1,359[23]. Of note, number of CKD cases could not be extracted from the validation work by Bradshaw *et al*[24]. The ratio of outcome events per number of candidate predictors in the derivation models ranged from 2.3[25] to 135.3[24]. This ratio could not be calculated for the derivation models by Wen *et al*[26] and Wu *et al*[27]. Across all reports, missing data were handled by conducting a complete-case analysis;[22-29] this information was not available in the study by Thakkinstian's *et al*[30] (Table 2; S3 Table).

What has been done?

In 2020, Asgari and colleagues prospectively validated a model from the Netherlands for 6- and 9-years CKD prediction, i.e. they provided estimates for two model validations.[23] In 2019, Bradshaw *et al* used cross-sectional data to derive four models, one of them was validated on two populations (rural and urban), i.e. they provided estimates for six models (four derivations and two validations).[24] In 2017, Carrillo-Larco *et al* used cross-sectional data to derive and validate two models, i.e., they provided estimates for four models (two derivations and two validations).[25] In 2015, Mogueo *et al* used cross-sectional data to validate two models that were previously developed in Korea and Thailand using two different outcome definitions for each model, i.e., they provided estimates for four model validations.[22] In 2017, Saranburut *et al* prospectively validated the Framingham Heart Study risk score on a cohort using two different outcome definitions, i.e., they provided estimates for two model validations.[28] In 2017, Saranburut *et al* prospectively developed four models and validated two of them using cohort data, i.e., they provided estimates for six models (four derivations and two validations).[29] In 2011, Thakkinstian *et al* derived one model using cross-sectional data.[30] In 2020, Wen *et al* prospectively derived two models.[26] In 2016, Wu *et al* used cross-sectional data to derive and validate one model, i.e., they provided estimates for two models (one derivation and one

For peer review only - http://bmjopen.bmj.com/site/about/guidelines.xhtml

validation).[27] Overall, fourteen models were derived and fifteen underwent validation (hence the 29 rows in Tables 2, 3 and 4).

Outcome ascertainment

Across all reports, CKD was defined as eGFR <60 mL/min/1.73m^2 [22-30] assessed by either the Modification of Diet Renal Disease (MDRD) formula[22 23 25 26 28 30] or the Chronic Kidney Disease Epidemiology Collaboration (CKD-EPI) formula.[24 27-29] In addition to the eGFR assessment, Bradshaw *et al*[24] and Wen *et al*[26] defined CKD as a urinary albumin-to-creatinine ratio (UACR) ≥30 mg/g. Mogueo *et al* validations also considered CKD as any nephropathy including stages I to V of the “Kidney Disease: Improving Global Outcomes (KDIGO)” classification.[22] Thakkinstian *et al*, also considered CKD as eGFR ≥60 mL/min/1.73m^2 if it had haematuria or UACR ≥30 mg/g [30] (Table 2).

Predictors and modelling

Logistic regression analysis was conducted in all derivation models.[24-27 29 30] Selection of the final predictors was based on modelling techniques: backward[24 25] and forward selection[26 27 29 30] (S3 Table). All studies categorized numerical variables. The most frequent predictors included in the models were: age, diabetes mellitus and sex (S2 Figure).

Model performance

All studies reported calibration and discrimination metrics, except for the validations by Bradshaw *et al*[24] and Carrillo Larco *et al*[25] (S3 Table). Regarding discrimination metrics, the area under the Receiver Operating Characteristic (ROC) curve and C-statistic were over 63%[28] and 70%,[24] respectively. Among all studies, sensibility ranged from 56.8%[26] to 84.0%,[22] specificity ranged from 65.1%[26] to 86.3%,[27] positive predictive value (PPV) ranged from 8.8%[25] to 33.8%,[26] and negative predictive value (NPV) ranged from 89.4%[26] to 99.1%.[25] The NPV was the best metric, consistently above 89.4% (Table 3).

For peer review only - http://bmjopen.bmj.com/site/about/guidelines.xhtml

Risk of bias

All studies showed a high risk of bias due to insufficient or inadequate analytical reporting. The flaw regarding the analysis criteria can be explained by how original reports handled missing data and predictors categorization. The participants and predictors criteria had low risk of bias in most of the reports. Most of the individual reports demonstrated an inappropriate evaluation of performance metrics.[23 25-30] Low applicability concern was noted (Table 4; S4Table).

DISCUSSION

Main findings

This systematic review summarized all available risk scores for CKD in LMIC. In so doing, we provided the most comprehensive list of CKD risk scores to enhance primary prevention and early diagnosis of CKD in LMIC. Although the available models had acceptable discrimination metrics and, when available, acceptable calibration metrics, these models had serious methodological limitations such as a reduced number of outcome events. The best performance metric across risk scores was the negative predictive value. Overall, CKD risk prediction tools in LMIC need rigorous development and validation so that they can be incorporated into clinical practice and interventions. The available evidence would not support using any of the available CKD risk scores across LMIC.

Limitations of the review

We did not search grey literature. We argue that this limitation would not substantially change our results because these sources are most likely not to have included a random sample of the general population, and are likely to have included a small sample size with few outcome events. That is, we would not expect to find a report in the grey literature with a much better methodology than that of the studies herein summarised.

Limitations of the selected reports

Several LMIC do not have a CKD risk score, particularly countries in Central America and Oceania. This should encourage public health officers and researchers to develop CKD prediction models. They could conduct new epidemiological studies or leverage on available health surveys with kidney biomarkers. These models could have pragmatic and direct applications in clinical medicine, by providing a tool for early identification of CKD cases. Similarly, these models could inform public

For peer review only - http://bmjopen.bmj.com/site/about/guidelines.xhtml

health interventions and planning, by providing a tool to quantify the size of the population likely to have or to develop CKD.

Clinical guidelines state that CKD is defined as a sustained structural or functional kidney damage for ≥3 months.[31] In the studies herein summarised, CKD was defined at one point in time. Future work could expand the definition of CKD to also incorporate the lapse during which the patient had kidney damage. In addition, different procedures were used to define CKD including eGFR, proteinuria, and UACR. Even amongst those studies in which CKD was defined with eGFR, they used different equations to compute the eGFR. Researchers and practitioners in LMIC could agree on the best and most pragmatic as well as cost-effective definition of CKD, so that future models could use this definition. This would improve the comparability and extrapolability of the models.

All reports in which a new CKD risk score was developed selected the predictors through univariate analyses,[24-27 29 30] which is not be the best approach to choose predictors.[32-34] Ideally, predictors should be selected based on expert knowledge, or amongst those with the strongest association evidence with CKD. In a similar vein, predictors selection should be guided by the target population. For example, CKD prediction models for populations in LMIC should prioritize simple biomarkers or inexpensive clinical evaluations (e.g., blood pressure). In this way, the risk score is likely to be used in clinical practice in resource-limited settings. Another relevant methodological limitation was how the original reports handled missing data. To the extent possible, multiple imputation should be implemented to maximize available data and to avoid potential bias by studying only observations with complete information. Finally, calibration metrics need to be consistently reported and should inform the direction of the miscalibration. Most of the studies used the Hosmer-Lemeshow X^2 test as the calibration metric. Unfortunately, this test does not inform on whether the model prediction is overestimating or underestimating the observed risk; calibration plots are a useful alternative.

Clinical and public health relevance

The Latin American Society of Nephrology and Hypertension (Sociedad Latinoamericana de Nefrología e Hipertensión - SLANH) recommends to annually screen for CKD with several studies: blood pressure, serum creatinine, proteinuria and urinalysis.[35] The South African Renal Society (SARS) guidelines also recommend CKD screening annually, yet they focus on high-risk populations: people with diabetes, hypertension, or HIV.[36] This recommendation is endorsed by the Asian Forum

For peer review only - http://bmjopen.bmj.com/site/about/guidelines.xhtml

for Chronic Kidney Disease Initiatives (AFCKDI), extending it to individuals ≥65 years, people consuming nephrotoxic substances, and those with family history of CKD and past history of acute kidney injury.[37] Although it seems reasonable to screen people with risk factors such as hypertension and diabetes, this approach may miss a large proportion of the high-risk population because they could be unaware of their condition.[38 39] In this case, risk scores could be useful because they can be applied to large populations regardless of whether they are aware of their hypertension or diabetes status. Unfortunately, our work would not support nor encourage the inclusion of available risk scores for CKD in clinical guidelines in LMIC. Instead, our results urgently call to improve risk prediction research in LMIC. Therefore, CKD risk scores could be included into clinical practice to identify high-risk individuals and to inform the patient's management plan as it the case in other fields such as cardiovascular primary prevention.

Conclusions

This systematic review of diagnostic and prognostic models of CKD did not find conclusive evidence to recommend the use of a single CKD score across LMIC. Nonetheless, we identified relevant efforts in Iran, India, Peru, South Africa, China and Thailand; these models would require further external validation before they can be applied in other LMIC. We encourage researchers and practitioners to develop and validate CKD risk scores, which are cost-efficient tools to early identify CKD prevalent and incident cases so that they can receive timely treatment.

For peer review only

For peer review only - http://bmjopen.bmj.com/site/about/guidelines.xhtml

Contributors: RMC-L, DJA-G and EJA conceived the idea. RMC-L, DJA-G and EJA conducted the search. DJA-G and EJA wrote the manuscript. All authors approved the submitted version.

Funding: RMC-L is supported by a Wellcome Trust International Training Fellowship (214185/Z/18/Z).

Competing interests: None declared.

Patient consent for publication: Not required.

Data availability statement: Data sharing not applicable as no data sets generated for this study. Given the nature of systematic reviews, the data set generated and analysed for the current study is already available. All studies analysed for the present review are referenced for readers.

For peer review only

For peer review only - http://bmjopen.bmj.com/site/about/guidelines.xhtml

REFERENCES

1. Bikbov B, Purcell CA, Levey AS, et al. Global, regional, and national burden of chronic kidney disease, 1990–2017: a systematic analysis for the Global Burden of Disease Study 2017. *The Lancet* 2020;395(10225):709-33. doi: 10.1016/s0140-6736(20)30045-3
2. Foreman KJ, Marquez N, Dolgert A, et al. Forecasting life expectancy, years of life lost, and all-cause and cause-specific mortality for 250 causes of death: reference and alternative scenarios for 2016–40 for 195 countries and territories. *The Lancet* 2018;392(10159):2052-90. doi: 10.1016/s0140-6736(18)31694-5 [published Online First: 2018/10/21]
3. Xie Y, Bowe B, Mokdad AH, et al. Analysis of the Global Burden of Disease study highlights the global, regional, and national trends of chronic kidney disease epidemiology from 1990 to 2016. *Kidney International* 2018;94(3):567-81. doi: 10.1016/j.kint.2018.04.011
4. Yarnoff BO, Hoerger TJ, Simpson SK, et al. The cost-effectiveness of using chronic kidney disease risk scores to screen for early-stage chronic kidney disease. *BMC Nephrology* 2017;18(1):85. doi: 10.1186/s12882-017-0497-6 [published Online First: 2017/03/16]
5. Samanta R, Misir R, Mitra M. A reduced set of features for chronic kidney disease prediction. *Journal of Pathology Informatics* 2017;8(1):24. doi: 10.4103/jpi.jpi_88_16 [published Online First: 2017/07/15]
6. Chang H-L, Wu C-C, Lee S-P, et al. A predictive model for progression of CKD. *Medicine* 2019;98(26):e16186. doi: 10.1097/md.0000000000016186 [published Online First: 2019/07/03]
7. Lerner B, Desrochers S, Tangri N. Risk Prediction Models in CKD. *Seminars in Nephrology* 2017;37(2):144-50. doi: 10.1016/j.semnephrol.2016.12.004 [published Online First: 2017/04/16]
8. Tangri N, Inker LA, Hiebert B, et al. A Dynamic Predictive Model for Progression of CKD. *American Journal of Kidney Diseases* 2017;69(4):514-20. doi: 10.1053/j.ajkd.2016.07.030 [published Online First: 2016/10/04]
9. Ramspek CL, de Jong Y, Dekker FW, et al. Towards the best kidney failure prediction tool: a systematic review and selection aid. *Nephrology Dialysis Transplantation* 2020;35(9):1527-38. doi: 10.1093/ndt/gfz018
10. Remuzzi G, Echouffo-Tcheugui JB, Kengne AP. Risk Models to Predict Chronic Kidney Disease and Its Progression: A Systematic Review. *PLoS medicine* 2012;9(11):e1001344. doi: 10.1371/journal.pmed.1001344 [published Online First: 2012/11/28]
11. Page MJ, McKenzie JE, Bossuyt PM, et al. The PRISMA 2020 statement: An updated guideline for reporting systematic reviews. *PLoS medicine* 2021;18(3):e1003583. doi: 10.1371/journal.pmed.1003583
12. Ascencio EJ, Aparcana-Granda DJ, Carrillo-Larco RM. Chronic kidney disease in Low- and Middle-Income Countries: Protocol for a systematic review of diagnostic and prognostic models. *medRxiv* 2021:2021.04.24.21256041. doi: 10.1101/2021.04.24.21256041
13. Debray TPA, Damen JAAG, Snell KIE, et al. A guide to systematic review and meta-analysis of prediction model performance. *Bmj* 2017;356:i6460. doi: 10.1136/bmj.i6460 [published Online First: 2017/01/07]
14. Moons KGM, de Groot JAH, Bouwmeester W, et al. Critical Appraisal and Data Extraction for Systematic Reviews of Prediction Modelling Studies: The CHARMS Checklist. *PLoS medicine* 2014;11(10):e1001744. doi: 10.1371/journal.pmed.1001744 [published Online First: 2014/10/15]
15. The World Bank. World Bank Country and Lending Groups [Available from: https://datahelpdesk.worldbank.org/knowledgebase/articles/906519-world-bank-country-and-lending-groupsfiles/235/906519-world-bank-country-and-lending-groups.html.
16. Ouzzani M, Hammady H, Fedorowicz Z, et al. Rayyan—a web and mobile app for systematic reviews. *Systematic Reviews* 2016;5(1):210. doi: 10.1186/s13643-016-0384-4 [published Online First: 2016/12/07]

For peer review only - http://bmjopen.bmj.com/site/about/guidelines.xhtml

17. Moons KGM, Wolff RF, Riley RD, et al. PROBAST: A Tool to Assess Risk of Bias and Applicability of Prediction Model Studies: Explanation and Elaboration. *Annals of internal medicine* 2019;170(1):W1-W33. doi: 10.7326/m18-1377 [published Online First: 2019/01/01]
18. Wolff RF, Moons KGM, Riley RD, et al. PROBAST: A Tool to Assess the Risk of Bias and Applicability of Prediction Model Studies. *Annals of internal medicine* 2019;170(1):51-58. doi: 10.7326/m18-1376 [published Online First: 2019/01/01]
19. Zhou JH, Wei Y, Lyu YB, et al. Prediction of 6-year incidence risk of chronic kidney disease in the elderly aged 65 years and older in 8 longevity areas in China. *Zhonghua liu xing bing xue za zhi = Zhonghua liuxingbingxue zazhi* 2020;41(1):42-47. doi: 10.3760/cma.j.issn.0254-6450.2020.01.009 [published Online First: 2020/02/18]
20. Mahapatra H, Gupta Y, Sharma N, et al. Identification of high-risk population and prevalence of kidney damage among asymptomatic central government employees in Delhi, India. *Saudi Journal of Kidney Diseases and Transplantation* 2016;27(2):362-70. doi: 10.4103/1319-2442.178564
21. Alvarez-Gregori JA, Robles NR, Mena C, et al. The value of a formula including haematocrit, blood urea and gender (HUGE) as a screening test for chronic renal insufficiency. *The journal of nutrition, health & aging* 2011;15(6):480-84. doi: 10.1007/s12603-011-0001-0 [published Online First: 2011/05/31]
22. Mogueo A, Echouffo-Tcheugui JB, Matsha TE, et al. Validation of two prediction models of undiagnosed chronic kidney disease in mixed-ancestry South Africans. *BMC Nephrology* 2015;16(1):94. doi: 10.1186/s12882-015-0093-6 [published Online First: 2015/07/05]
23. Asgari S, Moosaie F, Khalili D, et al. External validation of the European risk assessment tool for chronic cardio-metabolic disorders in a Middle Eastern population. *Journal of Translational Medicine* 2020;18(1):267. doi: 10.1186/s12967-020-02434-5 [published Online First: 2020/07/04]
24. Bradshaw C, Kondal D, Montez-Rath ME, et al. Early detection of chronic kidney disease in low-income and middle-income countries: development and validation of a point-of-care screening strategy for India. *BMJ Global Health* 2019;4(5):e001644. doi: 10.1136/bmjgh-2019-001644 [published Online First: 2019/09/24]
25. Carrillo-Larco RM, Miranda JJ, Gilman RH, et al. Risk score for first-screening of prevalent undiagnosed chronic kidney disease in Peru: the CRONICAS-CKD risk score. *BMC Nephrology* 2017;18(1):343. doi: 10.1186/s12882-017-0758-4 [published Online First: 2017/12/01]
26. Wen J, Hao J, Zhang Y, et al. Risk scores for predicting incident chronic kidney disease among rural Chinese people: a village-based cohort study. 2020;21(1):120.
27. Wu L, Guo VY, Wong CKH, et al. Innovative non-invasive model for screening reduced estimated glomerular filtration rate in a working population. *Nephrology* 2017;22(11):892-98. doi: 10.1111/nep.12921 [published Online First: 2016/09/16]
28. Saranburut K, Vathesatogkit P, Chittamma A, et al. Evaluation of the Framingham Heart Study risk factors and risk score for incident chronic kidney disease at 10 years in a Thai general population. 2017;49(5):851-57.
29. Saranburut K, Vathesatogkit P, Thongmung N, et al. Risk scores to predict decreased glomerular filtration rate at 10 years in an Asian general population. 2017;18(1):240.
30. Thakkinstian A, Ingsathit A, Chaiprasert A, et al. A simplified clinical prediction score of chronic kidney disease: A cross-sectional-survey study. *BMC Nephrology* 2011;12(1):45. doi: 10.1186/1471-2369-12-45 [published Online First: 2011/09/29]
31. Kidney Disease: Improving Global Outcomes (KDIGO) CKD Work Group. KDIGO 2012 clinical practice guideline for the evaluation and management of chronic kidney disease. *Kidney Int Suppl* 2013;3:1-150.
32. Harrell FE. Regression Modeling Strategies, With Applications to Linear Models, Logistic Regression, and Survival Analysis. New York: Springer 2001.

For peer review only - http://bmjopen.bmj.com/site/about/guidelines.xhtml

33. Steyerberg EW. Clinical Prediction Models: A Practical Approach to Development, Validation, and Updating. New York: Springer 2009.
34. Sun G-W, Shook TL, Kay GL. Inappropriate use of bivariable analysis to screen risk factors for use in multivariable analysis. *Journal of clinical epidemiology* 1996;49(8):907-16. doi: 10.1016/0895-4356(96)00025-x [published Online First: 1996/08/01]
35. Sociedad Latinoamericana de Nefrología e H. Guías Latinoamericanas de Práctica Clínica sobre la Prevención, Diagnóstico y Tratamiento de los Estadios 1-5 de la Enfermedad Renal Crónica. *1* 2012
36. South African Renal Society. Guideline for the optimal care of patients on chronic dialysis in South Africa. 2015
37. Li PK-T. Asian chronic kidney disease best practice recommendations: Positional statements for early detection of chronic kidney disease from Asian Forum for Chronic Kidney Disease Initiatives (AFCKDI). *Nephrology* 2011;16:633-41.
38. Wareham NJ, Manne-Goehler J, Geldsetzer P, et al. Health system performance for people with diabetes in 28 low- and middle-income countries: A cross-sectional study of nationally representative surveys. *PLoS medicine* 2019;16(3):e1002751. doi: 10.1371/journal.pmed.1002751
39. Zhou B, Carrillo-Larco RM, Danaei G, et al. Worldwide trends in hypertension prevalence and progress in treatment and control from 1990 to 2019: a pooled analysis of 1201 population-representative studies with 104 million participants. *The Lancet* 2021 doi: 10.1016/s0140-6736(21)01330-1 [published Online First: 2021/08/28]

For peer review only

For peer review only - http://bmjopen.bmj.com/site/about/guidelines.xhtml

TABLES:

Table 1. CHARMS criteria to define research question and strategy.

Concept	Criteria
Prognostic or diagnostic?	Both - this review focused on diagnostic and prognostic risk scores for chronic kidney disease (CKD)
Scope	Diagnostic/prognostic models to inform physicians, researchers and the general population whether they are likely to have CKD (i.e., diagnostic) or will be likely to have CKD (i.e., prognostic)
Type of prediction modelling studies	• Diagnostic/prognostic models with external validation<br>• Diagnostic/prognostic models without external validation<br>• Diagnostic/prognostic models validation
Target population to whom the prediction model applies	General adult population in Low- and Middle-Income Countries (LMIC). No age or gender restrictions
Outcome to be predicted	CKD (diagnostic or prognostic)
Time span of prediction	Any, prognostic models will not be included/excluded based on the prediction time span
Intended moment of using the model	Diagnostic/prognostic models to be used in asymptomatic adults of LMIC to ascertain current CKD status or future risk of developing CKD. These models could be used for screening, treatment allocation in primary prevention, or research purposes

Based on the CHARMS checklist.[14]

For peer review only

For peer review only - http://bmjopen.bmj.com/site/about/guidelines.xhtml

212 **Table 2. General characteristics.**

Nº of report	Study	Country	Outcome prevalence (%)	Mean age (years)	Men (%)	Outcome details	Baseline sample size	Number of outcome events	Outcome events per candidate predictors
1	Asgari *et al*, 2020 European Risk Assessment tool (6-years validation)	Iran	22.08	46.02	40.1	CKD was defined as eGFR <60 mL/min/1.73 m^2, provided by the MDRD formula	3,270	722	n/a
1	Asgari *et al*, 2020 European Risk Assessment tool (9-years validation)	Iran	41.94	NI	40.6	CKD was defined as eGFR <60 mL/min/1.73 m^2, provided by the MDRD formula	3,240	1,359	n/a
2	Bradshaw *et al*, 2019 - Model 1 (derivation)	India	10.89	44.9	46.8	CKD was defined as an eGFR rate <60 mL/min/1.73 m^2 (estimated with the CKD-EPI equation) or UACR ≥30 mg/g	8,698	947	31.6
2	Bradshaw *et al*, 2019 - Model 2 (derivation)	India	10.89	44.9	46.8	CKD was defined as an eGFR rate <60 mL/min/1.73 m^2 (estimated with the CKD-EPI equation) or UACR ≥30 mg/g	8,698	947	41.2
2	Bradshaw *et al*, 2019 - Model 3a (derivation)	India	10.89	44.9	46.8	CKD was defined as an eGFR rate <60 mL/min/1.73 m^2 (estimated with the CKD-EPI equation) or UACR ≥30 mg/g	8,698	947	135.3
2	Bradshaw *et al*, 2019 - Model 3b (derivation)	India	10.89	44.9	46.8	CKD was defined as an eGFR rate <60 mL/min/1.73 m^2 (estimated with the CKD-EPI equation) or UACR ≥30 mg/g	8,698	947	118.4

For peer review only - http://bmjopen.bmj.com/site/about/guidelines.xhtml

2	Bradshaw *et al*, 2019 - Model 3a (CARRS-I urban validation)	India	NI	NI	NI	CKD was defined as an eGFR rate <60 mL/min/1.73 m^2 (estimated with the CKD-EPI equation) or UACR ≥30 mg/g	4,065	NI	n/a
2	Bradshaw *et al*, 2019 - Model 3a (UDAY rural validation)	India	NI	NI	NI	CKD was defined as an eGFR rate <60 mL/min/1.73 m^2 (estimated with the CKD-EPI equation) or UACR ≥30 mg/g	4,940	NI	n/a
3	Carrillo-Larco *et al*, 2017 - CRONICAS-CKD (derivation complete)	Peru	3.42	57.7	49.4	CKD was defined as eGFR <60 mL/min/1.73 m^2, provided by the MDRD formula	2,368	81	2.25
3	Carrillo-Larco *et al*, 2017 - CRONICAS-CKD (derivation lab-free)	Peru	3.42	57.7	49.4	CKD was defined as eGFR <60 mL/min/1.73 m^2, provided by the MDRD formula	2,368	81	3.1
3	Carrillo-Larco *et al*, 2017 - CRONICAS-CKD (validation complete)	Peru	5.41	57.1	47.7	CKD was defined as eGFR <60 mL/min/1.73 m^2, provided by the MDRD formula	1,459	79	n/a
3	Carrillo-Larco *et al*, 2017 - CRONICAS-CKD (validation lab-free)	Peru	5.41	57.1	47.7	CKD was defined as eGFR <60 mL/min/1.73 m^2, provided by the MDRD formula	1,459	79	n/a
4	Mogueo *et al*, 2015 - Korean model (eGFR validation)	South Africa	28.71	55	23.4	CKD was defined as eGFR <60 mL/min/1.73 m^2, provided by the 4-variable MDRD formula	902	259	n/a
4	Mogueo *et al*, 2015 - Thai model (eGFR validation)	South Africa	28.71	55	23.4	CKD was defined as eGFR <60 mL/min/1.73 m^2, provided by the 4-variable MDRD formula	902	259	n/a
4	Mogueo *et al*, 2015 - Korean model (eGFR or proteinuria validation)	South Africa	29.71	55	23.4	CKD was defined as eGFR <60 mL/min/1.73 m^2, provided by the 4-variable MDRD formula and "any nephropathy" (Any of the stages I to V of the KDIGO classification)	902	268	n/a

For peer review only - http://bmjopen.bmj.com/site/about/guidelines.xhtml

4	Mogueo *et al*, 2015 - Thai model (eGFR or proteinuria validation)	South Africa	29.71	55	23.4	CKD was defined as eGFR <60 mL/min/1.73 m^2, provided by the 4-variable MDRD formula and "any nephropathy" (Any of the stages I to V of the KDIGO classification)	902	268	n/a
5	Saranburut *et al*, 2017 - Framingham Heart Study (MDRD validation)	Thailand	10.37	54.6	70.8	CKD was defined as eGFR <60 mL/min/1.73 m^2, provided by the MDRD formula	2,141	222	n/a
5	Saranburut *et al*, 2017 - Framingham Heart Study (CKD-EPI validation)	Thailand	10.01	54.7	71.5	CKD was defined as eGFR <60 mL/min/1.73 m^2, provided by the CKD-EPI equation	2,328	233	n/a
6	Saranburut *et al*, 2017 - Model 1 (derivation Clinical only)	Thailand	8.51	51.3	70.5	CKD was defined as a preserved GFR (eGFR ≥60 mL/min/1.73m^2) at baseline and subsequently developed decreased GFR (eGFR <60 mL/min/1.73m^2) at the 10 year follow-up, provided by the Two-level Race Variable CKD-EPI equation (using the non-black coefficient)	3,186	271	18.1
6	Saranburut *et al*, 2017 - Model 1 BMI (derivation Clinical only)	Thailand	8.51	51.3	70.5	CKD was defined as a preserved GFR (eGFR ≥60 mL/min/1.73m^2) at baseline and subsequently developed decreased GFR (eGFR <60 mL/min/1.73m^2) at the 10 year follow-up, provided by the Two-level Race Variable CKD-EPI equation (using the non-black coefficient)	3,186	271	18.1
6	Saranburut *et al*, 2017 - Model 2 (derivation Clinical + Limited laboratory tests)	Thailand	8.51	51.3	70.5	CKD was defined as a preserved GFR (eGFR ≥60 mL/min/1.73m^2) at baseline and subsequently developed decreased GFR (eGFR <60 mL/min/1.73m^2) at the 10 year follow-up, provided by the Two-level Race Variable CKD-EPI equation (using the non-black coefficient)	3,186	271	16.9

For peer review only - http://bmjopen.bmj.com/site/about/guidelines.xhtml

6	Saranburut *et al*, 2017 - Model 3 (derivation Clinical + Full laboratory tests)	Thailand	8.51	51.3	70.5	CKD was defined as a preserved GFR (eGFR ≥60 mL/min/1.73m²) at baseline and subsequently developed decreased GFR (eGFR <60 mL/min/1.73m²) at the 10 year follow-up, provided by the Two-level Race Variable CKD-EPI equation (using the non-black coefficient)	3,186	271	12.3
6	Saranburut *et al*, 2017 - Model 1 (validation Clinical only)	Thailand	1.94	45.6	70.5	CKD was defined as a preserved GFR (eGFR ≥60 mL/min/1.73m²) at baseline and subsequently developed decreased GFR (eGFR <60 mL/min/1.73m²) at the 10 year follow-up, provided by the Two-level Race Variable CKD-EPI equation (using the non-black coefficient)	1,395	27	n/a
6	Saranburut *et al*, 2017 - Model 2 (validation Clinical + Limited laboratory tests)	Thailand	1.94	45.6	70.5	CKD was defined as a preserved GFR (eGFR ≥60 mL/min/1.73m²) at baseline and subsequently developed decreased GFR (eGFR <60 mL/min/1.73m²) at the 10 year follow-up, provided by the Two-level Race Variable CKD-EPI equation (using the non-black coefficient)	1,395	27	n/a
7	Thakkinstian *et al*, 2011 (derivation)	Thailand	18.10	45.2	45.5	CKD was defined as a combination of stages I to V. CKD stage I & II was defined as eGFR ≥90 and eGFR 60-89 ml/min/1.73 m², respectively; with haematuria or UACR ≥30 mg/g. CKD stage III, IV, and V was defined as eGFR 30-59, 15-29, and <15 ml/min/1.73 m², respectively; regardless of kidney damage (eGFR was calculated using the MDRD formula)	3,459	626	16.9
8	Wen *et al*, 2020 - Simple Risk Score (derivation)	China	18.06	50	44.7	CKD was defined as an eGFR rate <60 mL/min/1.73 m² (assessed with the modified Chinese MDRD equation) or UACR ≥30 mg/g	3,266	590	NI

8	Wen *et al*, 2020 - Best-fit Risk Score (derivation)	China	18.06	50	44.7	CKD was defined as an eGFR rate <60 mL/min/1.73 m^2 (assessed with the modified Chinese MDRD equation) or UACR ≥30 mg/g	3,266	590	NI
9	Wu *et al*, 2016 (derivation)	China	2.05	45.3	56.7	CKD was defined as eGFR <60 mL/min/1.73 m^2, provided by the CKD-EPI equation	14,374	294	NI
9	Wu *et al*, 2016 (validation)	China	1.10	41.8	63.7	CKD was defined as eGFR <60 mL/min/1.73 m^2, provided by the CKD-EPI equation	4,371	48	n/a

CKD, chronic kidney disease; CKD-EPI, chronic kidney disease epidemiology collaboration; eGFR, estimated glomerular filtration rate; GFR, glomerular filtration rate; KDIGO, MDRD, modification of diet renal disease; n/a, not applicable; NI, no information; UACR, urinary albumin-to-creatinine ratio.

For peer review only

For peer review only - http://bmjopen.bmj.com/site/about/guidelines.xhtml

Table 3. Performance metrics.

Nº	Study	Discrimination (%)	Classification measures
1	Asgari *et al*, 2020 European Risk Assessment tool (6-years validation)	AUC (95% CI) for final intercept adjusted model = Male: 76 (72-79) and Female: 71 (69-73)	For men at a cut-off of 25: sensibility=72.7%; specificity=67.6%. For women at a cut-off of 19: sensibility=66.8%; specificity=65.6%
1	Asgari *et al*, 2020 European Risk Assessment tool (9-years validation)	AUC (95% CI) for final intercept adjusted model = Male: 71 (67-74) and Female: 70 (68-73)	For men at a cut-off of 25: sensibility=64.5%; specificity=69.5%. For women at a cut-off of 23: sensibility=56.9%; specificity=76.6%
2	Bradshaw *et al*, 2019 - Model 1 (derivation)	C-statistic (95% CI) = 79 (78-81)	At a cut-off of 0.09: sensibility=72%; specificity=72%; positive predictive value=24%; negative predictive value=96%
2	Bradshaw *et al*, 2019 - Model 2 (derivation)	C-statistic (95% CI) = 73 (72-75)	At a cut-off of 0.09: sensibility=68%; specificity=67%; positive predictive value=20%; negative predictive value=95%
2	Bradshaw *et al*, 2019 - Model 3a (derivation)	C-statistic (95% CI) = 77 (75-79)	At a cut-off of 0.09: sensibility=71%; specificity=70%; positive predictive value=22%; negative predictive value=95%
2	Bradshaw *et al*, 2019 - Model 3b (derivation)	C-statistic (95% CI) = 77 (76-79)	At a cut-off of 0.09: sensibility=71%; specificity=70%; positive predictive value=22%; negative predictive value=95%
2	Bradshaw *et al*, 2019 - Model 3a (CARRS-I urban validation)	C-statistic (95% CI) = 74 (73-74)	NI
2	Bradshaw *et al*, 2019 - Model 3a (UDAY rural validation)	C-statistic (95% CI) = 70 (69-71)	NI

For peer review only

For peer review only - http://bmjopen.bmj.com/site/about/guidelines.xhtml

3	Carrillo-Larco *et al*, 2017 - CRONICAS-CKD (derivation complete)	AUC = 76.2	At a cut-off of 2: sensibility=82.5%; specificity=70.0%; positive predictive value=8.8%; negative predictive value=99.1%; likelihood ratio positive=2.8; likelihood ratio negative=0.3
3	Carrillo-Larco *et al*, 2017 - CRONICAS-CKD (derivation lab-free)	AUC = 76	At a cut-off of 2: sensibility=80%; specificity=72%; positive predictive value=9.1%; negative predictive value=99%; likelihood ratio positive=2.9; likelihood ratio negative=0.3
3	Carrillo-Larco *et al*, 2017 - CRONICAS-CKD (validation complete)	AUC = 70	At a cut-off of 2: sensibility=70.5%; specificity=69.1%; positive predictive value=11.4%; negative predictive value=97.6%; likelihood ratio positive=2.3; likelihood ratio negative=0.4
3	Carrillo-Larco *et al*, 2017 - CRONICAS-CKD (validation lab-free)	AUC = 70	At a cut-off of 2: sensibility=70.5%; specificity=69.7%; positive predictive value=11.6%; negative predictive value=97.7%; likelihood ratio positive=2.3; likelihood ratio negative=0.4
4	Mogueo *et al*, 2015 - Korean model (eGFR validation)	C-statistic (95% CI) = 79.7 (76.5-82.9)	At a cut-off of 0.30: sensibility=82%; specificity=67%
4	Mogueo *et al*, 2015 - Thai model (eGFR validation)	C-statistic (95% CI) = 76 (72.6-79.3)	At a cut-off of 0.31: sensibility=73%; specificity=72%
4	Mogueo *et al*, 2015 - Korean model (eGFR or proteinuria validation)	C-statistic (95% CI) = 81.1 (78.0-84.2)	At a cut-off of 0.31: sensibility=84%; specificity=68%
4	Mogueo *et al*, 2015 - Thai model (eGFR or proteinuria validation)	C-statistic (95% CI) = 77.2 (73.9-80.5)	At a cut-off of 0.32: sensibility=74%; specificity=73%

For peer review only - http://bmjopen.bmj.com/site/about/guidelines.xhtml

For peer review only

5	Saranburut *et al*, 2017 - Framingham Heart Study (MDRD validation)	AUC (95% CI) = 69 (66-73)	NI
5	Saranburut *et al*, 2017 - Framingham Heart Study (CKD-EPI validation)	AUC (95% CI) = 63 (57-65)	NI
6	Saranburut *et al*, 2017 - Model 1 (derivation Clinical only)	AUC (95% CI) = 72 (69-75)	NI
6	Saranburut *et al*, 2017 - Model 1 BMI (derivation Clinical only)	AUC (95% CI) = 72 (69-75)	NI
6	Saranburut *et al*, 2017 - Model 2 (derivation Clinical + Limited laboratory tests)	AUC (95% CI) = 79 (76-82)	NI
6	Saranburut *et al*, 2017 - Model 3 (derivation Clinical + Full laboratory tests)	AUC (95% CI) = 80 (77-82)	NI
6	Saranburut *et al*, 2017 - Model 1 (validation Clinical only)	AUC (95% CI) = 66 (55-78)	NI
6	Saranburut *et al*, 2017 - Model 2 (validation Clinical + Limited laboratory tests)	AUC (95% CI) = 88 (80-95)	NI
7	Thakkinstian *et al*, 2011 (derivation)	C-statistic of internal validation = 74.1	At a cut-off of 5: sensitivity=76%; specificity=69%

For peer review only

8	Wen *et al*, 2020 - Simple Risk Score (derivation)	AUC (95% CI) = 71.7 (68.9-74.4)	At a cut-off of 14: sensitivity=70.5%; specificity=65.1%; positive predictive value=29.8%; negative predictive value=91.3%; likelihood ratio positive=2.0; likelihood ratio negative=0.5
8	Wen *et al*, 2020 - Best-fit Risk Score (derivation)	AUC (95% CI) = 72.1 (69.3-74.8)	At a cut-off of 24: sensitivity=56.8%; specificity=76.6%; positive predictive value=33.8%; negative predictive value=89.4%; likelihood ratio positive=2.4 likelihood ratio negative=0.6
9	Wu *et al*, 2016 (derivation)	AUC (95% CI) of internal validation = 89.4 (86.1-92.6)	At a cut-off of 36: sensitivity=82%; specificity=86.3%
9	Wu *et al*, 2016 (validation)	AUC (95% CI) = 88.0 (82.9-93.1)	NI

AUC, area under the curve; CI, confident interval; NI, no information.

For peer review only

217 **Table 4: Risk of bias assessment of individual diagnostic/prediction models**

Study	Objective	Risk of Bias (RoB)				Applicability			Overall	
		Participants	Predictors	Outcome	Analysis	Participants	Predictors	Outcome	RoB	Applicability
Asgari *et al*, 2020 European Risk Assessment tool (6-years)	Validation	+	+	?	-	+	+	+	-	+
Asgari *et al*, 2020 European Risk Assessment tool (9-years)	Validation	+	+	?	-	+	+	+	-	+
Bradshaw *et al*, 2019 - Model 1	Derivation	+	+	?	-	+	+	+	-	+
Bradshaw *et al*, 2019 - Model 2	Derivation	+	+	?	-	+	+	+	-	+
Bradshaw *et al*, 2019 - Model 3a	Derivation	+	+	?	-	+	+	+	-	+
Bradshaw *et al*, 2019 - Model 3b	Derivation	+	+	?	-	+	+	+	-	+
Bradshaw *et al*, 2019 - Model 3a (CARRS-I urban)	Validation	+	+	?	-	+	+	+	-	+
Bradshaw *et al*, 2019 - Model 3a (UDAY rural)	Validation	+	+	?	-	+	+	+	-	+
Carrillo-Larco *et al*, 2017 - CRONICAS-CKD (complete)	Derivation	+	+	+	-	+	+	+	-	+
Carrillo-Larco *et al*, 2017 - CRONICAS-CKD (lab-free)	Derivation	+	+	+	-	+	+	+	-	+
Carrillo-Larco *et al*, 2017 - CRONICAS-CKD (complete)	Validation	+	+	+	-	+	+	+	-	+

For peer review only - http://bmjopen.bmj.com/site/about/guidelines.xhtml

Carrillo-Larco *et al*, 2017 - CRONICAS-CKD (lab-free)	Validation	+	+	+	-	+	+	+	-	+
Mogueo *et al*, 2015 - Korean model (eGFR)	Validation	+	+	?	-	+	+	+	-	+
Mogueo *et al*, 2015 - Thai model (eGFR)	Validation	+	+	?	-	+	+	+	-	+
Mogueo *et al*, 2015 - Korean model (eGFR or proteinuria)	Validation	+	+	?	-	+	+	+	-	+
Mogueo *et al*, 2015 - Thai model (eGFR or proteinuria)	Validation	+	+	?	-	+	+	+	-	+
Saranburut *et al*, 2017 - Framingham Heart Study (MDRD)	Validation	+	+	?	-	+	+	+	-	+
Saranburut *et al*, 2017 - Framingham Heart Study (CKD-EPI)	Validation	+	+	?	-	+	+	+	-	+
Saranburut *et al*, 2017 - Model 1 (Clinical only)	Derivation	+	+	?	-	+	+	+	-	+
Saranburut *et al*, 2017 - Model 1 BMI (Clinical only)	Derivation	+	+	?	-	+	+	+	-	+
Saranburut *et al*, 2017 - Model 2 (Clinical + Limited laboratory tests)	Derivation	+	+	?	-	+	+	+	-	+
Saranburut *et al*, 2017 - Model 3 (Clinical + Full laboratory tests)	Derivation	+	+	?	-	+	+	+	-	+
Saranburut *et al*, 2017 - Model 1 (Clinical only)	Validation	+	+	?	-	+	+	+	-	+

For peer review only

For peer review only - http://bmjopen.bmj.com/site/about/guidelines.xhtml

Saranburut *et al*, 2017 - Model 2 (Clinical + Limited laboratory tests)	Validation	+	+	?	-	+	+	+	-	+
Thakkinstian *et al*, 2011	Derivation	+	+	?	-	+	+	+	-	+
Wen *et al*, 2020 - Simple Risk Score	Derivation	+	+	?	-	+	+	+	-	+
Wen *et al*, 2020 - Best-fit Risk Score	Derivation	+	+	?	-	+	+	+	-	+
Wu *et al*, 2016	Derivation	+	+	?	-	+	+	+	-	+
Wu *et al*, 2016	Validation	+	+	?	-	+	+	+	-	+

PROBAST = Prediction model Risk of Bias ASsessment Tool;[17 18] RoB = risk of bias. + indicates low RoB/low concern regarding applicability; − indicates high RoB/high concern regarding applicability; and ? indicates unclear RoB/unclear concern regarding applicability.

For peer review only

FIGURES

Figure 1. PRISMA 2020 flow diagram.

For peer review only

For peer review only - http://bmjopen.bmj.com/site/about/guidelines.xhtml

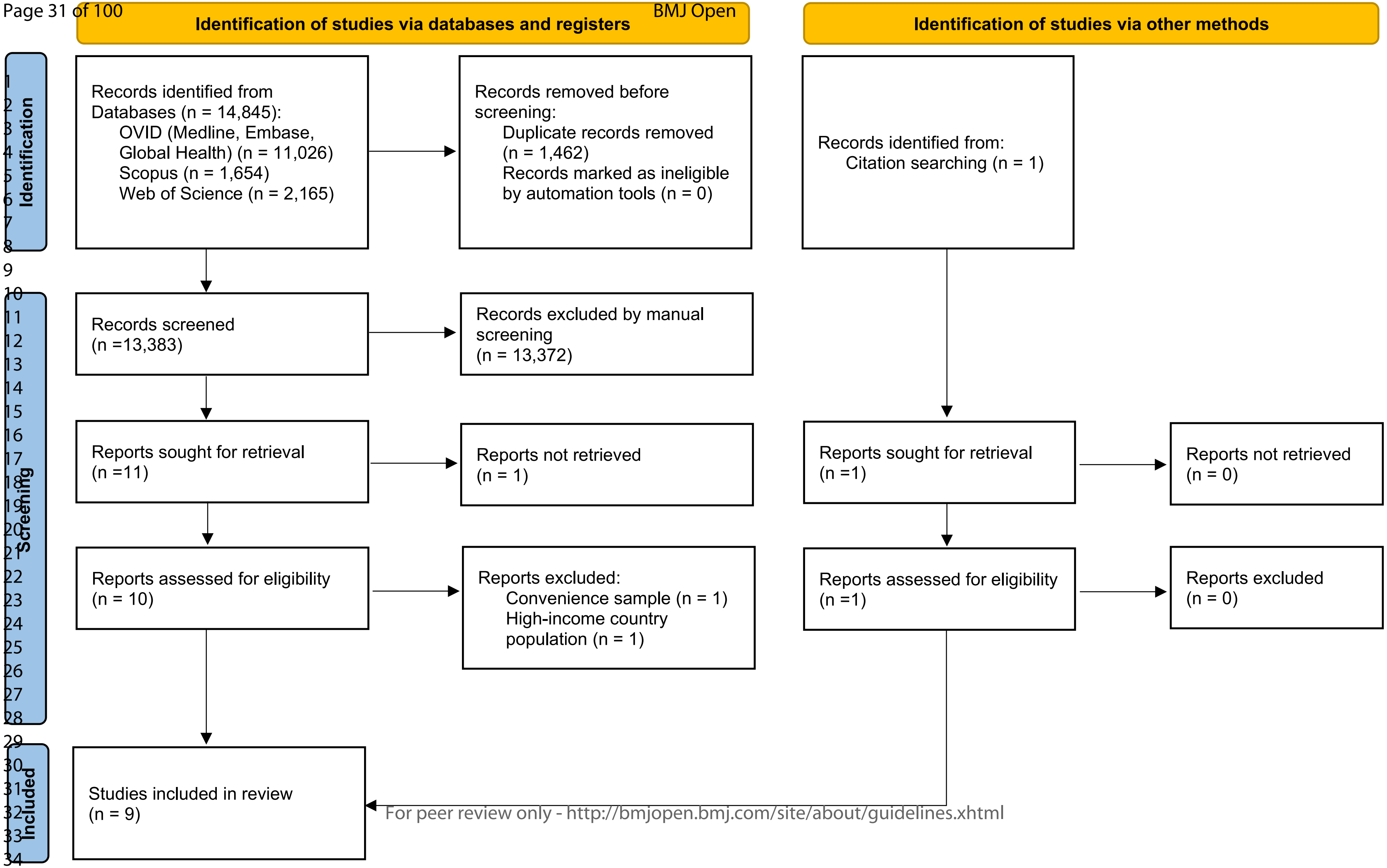
Identification of studies via databases and registers
Identification of studies via other methods
Identification
Records identified from
Databases (n = 14,845):
OVID (Medline, Embase, Global Health) (n = 11,026)
Scopus (n = 1,654)
Web of Science (n = 2,165)
Records removed before screening:
Duplicate records removed (n = 1,462)
Records marked as ineligible by automation tools (n = 0)
Records identified from:
Citation searching (n = 1)
Screening
Records screened
(n =13,383)
Records excluded by manual screening
(n = 13,372)
Reports sought for retrieval
(n =11)
Reports not retrieved
(n = 1)
Reports sought for retrieval
(n =1)
Reports not retrieved
(n = 0)
Reports assessed for eligibility
(n = 10)
Reports excluded:
Convenience sample (n = 1)
High-income country population (n = 1)
Reports assessed for eligibility
(n =1)
Reports excluded
(n = 0)
Included
Studies included in review
(n = 9)

Supplementary Material

. A systematic review of diagnostic and prognostic models of Chronic kidney disease in Low- and Middle- Income Countries

For peer review only

For peer review only - http://bmjopen.bmj.com/site/about/guidelines.xhtml

S1 Text: Protocol (also available at https://doi.org/10.1101/2021.04.24.21256041) 1

S1 Table: PRISMA Checklist 6

S2 Table: Search terms 11

S2.1 Table: Embase, Medline and Global Health (OVID) 11

S2.2 Table: SCOPUS 12

S2.3 Table: WEB OF SCIENCE 14

S3 Table: Data extraction form (by chapters) 15

S3.1 Table: Source of data and participants 15

S3.2 Table:: Outcome 26

S3.3 Table: Candidate predictors 31

S3.4 Table: Sample size and missing data 44

S3.5 Table: Model development 46

S3.6 Table: Model performance 49

S3.7 Table: Results 53

S3.8 Table: Discussion 55

S4 Table: PROBAST 57

S4.1 Table: Risk of Bias (RoB) 57

S4.2 Table: Applicability 65

S1 Figure: Countries where studies were conducted. 67

S2 Figure: Predictors included in the final models. 68

For peer review only

For peer review only - http://bmjopen.bmj.com/site/about/guidelines.xhtml

S1 Text: **Protocol (also available at https://doi.org/10.1101/2021.04.24.21256041)**

Chronic Kidney Disease in Low- and Middle- Income Countries: Protocol for a systematic review of diagnostic and prognostic models

Edson J Ascencio[1,2]

Diego J Aparcana-Granda[1,3]

Rodrigo M Carrillo-Larco[3,4]

1. Facultad de Medicina Alberto Hurtado, Universidad Peruana Cayetano Heredia, Lima, Perú.
2. Emerge, Emerging Diseases and Climate Change Research Unit, School of Public Health and Administration, Universidad Peruana Cayetano Heredia, Lima, Peru
3. CRONICAS Centre of Excellence in Chronic Diseases, Universidad Peruana Cayetano Heredia, Lima, Peru
4. Department of Epidemiology and Biostatistics, School of Public Health, Imperial College London, London, UK

Corresponding author

Rodrigo M Carrillo-Larco, MD

Department of Epidemiology and Biostatistics

School of Public Health, Imperial College London, London W2 1PG, UK

E-mail: rcarrill@ic.ac.uk

Phone: +44 0 7578240395

Competing interests: The authors declare no conflict of interests.

Support: RMC-L is supported by a Wellcome Trust International Training Fellowship (214185/Z/18/Z).

ABSTRACT

Background: Chronic Kidney Disease (CKD) is a highly prevalent condition with a large disease burden globally. In low- and middle-income countries (LMIC) the CKD screening challenges the health system. This systematic and comprehensive search of all CKD diagnostic and prognostic models in LMIC will inform screening strategies in LMIC following a risk-based approach.

Objective: To summarize all multivariate diagnostic and prognostic models for CKD in adults in LMIC.

Methods: Systematic review. Without date or language restrictions we will search Embase, Medline, Global Health (these three through Ovid), SCOPUS and Web of Science. We seek multivariable diagnostic or prognostic models which included a random sample of the general population. We will screen titles and abstracts; we will then study the selected reports. Both phases will be done by two reviewers independently. Data extraction will be performed by two researchers independently using a pre-specified Excel form (CHARMS model). We will evaluate the risk of bias with the PROBAST tool.

Conclusion: This systematic review will provide the most comprehensive list and critical appraisal of diagnostic and prognostic models for CKD available for the general population in LMIC. This evidence could

For peer review only - http://bmjopen.bmj.com/site/about/guidelines.xhtml

inform policies and interventions to improve CKD screening in LMIC following a risk-based approach, maximizing limited resources and reaching populations with limited access to CKD screening tests. This systematic review will also reveal methodological limitations and research needs to improve CKD diagnostic and prognostic models in LMIC.

Keywords: Chronic Kidney Disease; Diagnostic Models; Prognostic Models; Low- and Middle-income countries.

INTRODUCTION

Chronic kidney disease (CKD) is a highly prevalent condition that contributes to a large part of disease burden globally. Between 1990 and 2017, the health metrics of CKD showed a bleak profile: mortality rate, incidence and kidney transplantation rate increased by 2.8%, 29.3% and 34.4%, respectively.[1] CKD led to 1.2 million deaths in 2017 and in the best-case scenario, mortality is projected to increase to 2.2 million deaths[2] and become the 5th cause of years of life lost (YLL) by 2040.[3] Currently, 2.5 million of patients receive kidney transplantation therapy and it is projected to increase to 5.4 million by 2030.[1] CKD also reveals disparities between low- and middle-income countries (LMIC) and high income countries (HIC); for example, the age-standardised disability-adjusted life-year (DALY) rate due to CKD was the highest in LMIC between 1990-2017.[4] In LMIC, that remain as resource-constrained settings, there is a need for optimization of the CKD screening strategies which usually challenge the health system.[5]

Risk equations or risk scores are a cost-effective alternative for CKD screening.[6] These equations are less invasive and accepted by the general population;[7] also, they require less resources like laboratory tests.[8] Many scores were developed in high-income countries,[9-11] and they may not be used in LMIC because their accuracy is better where they have been developed.[12] Current strategies for CKD screening suggest studying people with risk factors (e.g. diabetes, hypertension).[13-15] These recommendations rely on studies where albuminuria and proteinuria were used as screening tools for identifying CKD patients.[16] Nevertheless, a systematic review found that using risk scores allows screening of a larger population and therefore can be useful for detecting more CKD cases.[6]

To date, there are no systematic reviews of diagnostic or prognostic models for CKD with a focus on LMIC.[17, 18] This limits our knowledge of what tools we have to enhance CKD screening in LMIC; similarly, this dearth of evidence prevents from planning future research to overcome the limitations of available models. This will be the first systematic review to fill these knowledge gaps in LMIC to improve and complement the CKD screening programmes in LMIC.

METHODS

Objective

To synthesise CKD diagnostic and prognostic models for the adult population of LMIC.

Study design

This systematic review and meta-analysis will be conducted following the preferred reporting items for systematic reviews and meta-analyses (PRISMA) 2020 guidelines.[19] We will also adhere to the recommendations for systematic reviews of diagnostic and prognostic models following the CHARMS guidelines[20] and the PROBAST tool to assess risk of bias.[21]

Eligibility criteria

Participants/population: We will include the general adult population (18 years and above) of LMIC with no gender restrictions. Studies following a population-based random sampling approach will be included. We will only include populations from LMIC according to The World Bank.[22] Conversely, studies with a study population of only patients (e.g., people with hypertension) or high-risk individuals (e.g., smokers) will be excluded. We will exclude studies with LMIC populations outside a LMIC.

Intervention, exposure: None (this review is looking at CKD diagnostic and prognostic models in LMIC).

For peer review only - http://bmjopen.bmj.com/site/about/guidelines.xhtml

Comparator, control: None (this review is looking at CKD diagnostic and prognostic models in LMIC).

Outcome: Diagnostic and prognostic models for CKD. The CKD diagnosis should have been based on a laboratory or imaging test including: urine albumin- creatinine ratio, urine protein-creatinine ratio, albumin excretion ratio, urine sediment, kidney images, kidney biopsy or the estimated glomerular filtration rate (eGFR). In other words, research in which CKD diagnosis was based on self-reported information only will not be considered. However, if a study combined both self-reported information and a laboratory or imaging tests, this will be included.

Types of studies: Studies with an observational design will be included, which encompasses crosssectional (for diagnostic models) and prospective longitudinal studies (for prognostic models). If we retrieve any systematic review on this subject, we will revise its reference list to identify relevant original sources.

Literature Search and Data collation

The search will be conducted in five search engines: Embase, Medline, Global Health (these three through Ovid), SCOPUS and Web of Science. No date or language restrictions will be set. The complete search strategy can be found in Supplementary Material.

Titles and abstracts will be screened by two researchers independently (DJA-G and EJA), looking for studies that meet the selection criteria above detailed. Full-text reports of the selected publications will be studied by two researchers independently (DJA-G and EJA). Discrepancies at any stage will be solved by consensus or by a third party (RMC-L).

During the full-text phase, if there are any original reports in which the population, methodology or results are not clear enough to assess the inclusion/exclusion criteria, we will contact the corresponding author by email. We will wait for two weeks, if we receive no answer and cannot solve our doubts through other means, this report will be excluded based on the lack of clarity to assess inclusion/exclusion criteria.

We will record the reasons for exclusion in the full-text phase and summarize the number of included/excluded reports following the PRISMA flow diagram.

Data extraction

We will develop a data extraction form following the CHARMS recommendations.[20] Data extraction will be conducted by two researchers independently; discrepancies will be solved by consensus or by a third party (RMC-L).

Risk of bias of individual studies

The risk of bias assessment of individual reports will be conducted using the Prediction model Risk Of

Bias ASsessment Tool (PROBAST) tool.[21]

Statistical Analysis

A qualitative synthesis is planned, whereby we will narratively synthesise the findings from the selected studies. We will summarize the key elements from each report such as study design, study population and characteristics of the study population. Also, we will summarize the key features of the risk scores as provided by each report, including discrimination, calibration, sensitivity, specificity, and predictive values. A quantitative synthesis will be carried out if the included studies are found to be sufficiently homogenous and we have at least four original reports.

Ethics

This review did not directly include human subjects. We considered this work as 'low risk' and did not request approval by an Ethics Committee. Results and opinions included in this protocol, and those included in the final report, are the author's alone and do not represent those of the institutions to which they belong.

For peer review only - http://bmjopen.bmj.com/site/about/guidelines.xhtml

CONCLUSIONS

This systematic review will provide a comprehensive list of diagnostic and prognostic models for CKD for people in LMIC, along with their accuracy metrics. Currently, information lacks in LMIC where diagnostic and prognostic models could inform CKD screening strategies. Similarly, this work will elucidate the limitations of available diagnostic and prognostic models for CKD in LMIC, so that future research can be planned accordingly to overcome these caveats and deliver robust models to advance

CKD screening strategies in LMIC.

REFERENCES

1. Collaboration GBDCKD. Global, regional, and national burden of chronic kidney disease, 19902017: a systematic analysis for the Global Burden of Disease Study 2017. Lancet. 2020;395(10225):709-33.
2. Agudelo-Botero M, Valdez-Ortiz R, Giraldo-Rodriguez L, Gonzalez-Robledo MC, Mino-Leon D, Rosales-Herrera MF, et al. Overview of the burden of chronic kidney disease in Mexico: secondary data analysis based on the Global Burden of Disease Study 2017. BMJ Open. 2020;10(3):e035285.
3. Foreman KJ, Marquez N, Dolgert A, Fukutaki K, Fullman N, McGaughey M, et al. Forecasting life expectancy, years of life lost, and all-cause and cause-specific mortality for 250 causes of death: reference and alternative scenarios for 2016-40 for 195 countries and territories. Lancet. 2018;392(10159):2052-90.
4. Bikbov B, Purcell CA, Levey AS, Smith M, Abdoli A, Abebe M, et al. Global, regional, and national burden of chronic kidney disease, 1990–2017: a systematic analysis for the Global Burden of Disease Study 2017. The Lancet. 2020;395(10225):709-33.
5. Jafar TH, Ramakrishnan C, John O, Tewari A, Cobb B, Legido-Quigley H, et al. Access to CKD Care in Rural Communities of India: a qualitative study exploring the barriers and potential facilitators. BMC Nephrol. 2020;21(1):26.
6. Yarnoff BO, Hoerger TJ, Simpson SK, Leib A, Burrows NR, Shrestha SS, et al. The costeffectiveness of using chronic kidney disease risk scores to screen for early-stage chronic kidney disease. BMC Nephrol. 2017;18(1):85.
7. Bradshaw C, Kondal D, Montez-Rath ME, Han J, Zheng Y, Shivashankar R, et al. Early detection of chronic kidney disease in low-income and middle-income countries: development and validation of a point-of-care screening strategy for India. BMJ Glob Health. 2019;4(5):e001644.
8. Misir R, Mitra M, Samanta RK. A Reduced Set of Features for Chronic Kidney Disease Prediction. J Pathol Inform. 2017;8:24.
9. Chang HL, Wu CC, Lee SP, Chen YK, Su W, Su SL. A predictive model for progression of CKD.
10. Medicine (Baltimore). 2019;98(26):e16186.
11. Lerner B, Desrochers S, Tangri N. Risk Prediction Models in CKD. Semin Nephrol. 2017;37(2):144-50.
12. Tangri N, Inker LA, Hiebert B, Wong J, Naimark D, Kent D, et al. A Dynamic Predictive Model for Progression of CKD. American journal of kidney diseases : the official journal of the National Kidney Foundation. 2017;69(4):514-20.
13. Grant SW, Collins GS, Nashef SAM. Statistical Primer: developing and validating a risk prediction model. Eur J Cardiothorac Surg. 2018;54(2):203-8.
14. Boulware LE, Jaar BG, Tarver-Carr ME, Brancati FL, Powe NR. Screening for Proteinuria in US
15. AdultsA Cost-effectiveness Analysis. JAMA. 2003;290(23):3101-14.
16. Hoerger TJ, Wittenborn JS, Segel JE, Burrows NR, Imai K, Eggers P, et al. A health policy model of CKD: 2. The cost-effectiveness of microalbuminuria screening. American journal of kidney diseases : the official journal of the National Kidney Foundation. 2010;55(3):463-73.
17. Manns B, Hemmelgarn B, Tonelli M, Au F, Chiasson TC, Dong J, et al. Population based screening for chronic kidney disease: cost effectiveness study. BMJ. 2010;341:c5869.
18. Komenda P, Ferguson TW, Macdonald K, Rigatto C, Koolage C, Sood MM, et al. Costeffectiveness of primary screening for CKD: a systematic review. American journal of kidney diseases : the official journal of the National Kidney Foundation. 2014;63(5):789-97.
19. Echouffo-Tcheugui JB, Kengne AP. Risk models to predict chronic kidney disease and its progression: a systematic review. PLoS medicine. 2012;9(11):e1001344.
20. Ramspek CL, de Jong Y, Dekker FW, van Diepen M. Towards the best kidney failure prediction tool: a systematic review and selection aid. Nephrology Dialysis Transplantation. 2019;35(9):1527-38.

For peer review only - http://bmjopen.bmj.com/site/about/guidelines.xhtml

21. Page MJ, McKenzie JE, Bossuyt PM, Boutron I, Hoffmann TC, Mulrow CD, et al. The PRISMA 2020 statement: an updated guideline for reporting systematic reviews. BMJ. 2021;372:n71.
22. Moons KG, de Groot JA, Bouwmeester W, Vergouwe Y, Mallett S, Altman DG, et al. Critical appraisal and data extraction for systematic reviews of prediction modelling studies: the CHARMS checklist. PLoS medicine. 2014;11(10):e1001744.
23. Moons KGM, Wolff RF, Riley RD, Whiting PF, Westwood M, Collins GS, et al. PROBAST: A Tool to Assess Risk of Bias and Applicability of Prediction Model Studies: Explanation and Elaboration. Annals of internal medicine. 2019;170(1):W1-W33.
24. Bank TW. World Bank Country and Lending Groups 2021 [Available from: https://datahelpdesk.worldbank.org/knowledgebase/articles/906519-world-bank-countryand-lending-groups.

For peer review only

For peer review only - http://bmjopen.bmj.com/site/about/guidelines.xhtml

S1 Table: PRISMA Checklist

Section and Topic	Item #	Checklist item	Location where item is reported
**TITLE**			
Title	1	Identify the report as a systematic review.	page 01
**ABSTRACT**			
Abstract	2	See the PRISMA 2020 for Abstracts checklist.	page 02
**INTRODUCTION**			
Rationale	3	Describe the rationale for the review in the context of existing knowledge.	page 03
Objectives	4	Provide an explicit statement of the objective(s) or question(s) the review addresses.	page 04
**METHODS**			
Eligibility criteria	5	Specify the inclusion and exclusion criteria for the review and how studies were grouped for the syntheses.	page 04-05
Information sources	6	Specify all databases, registers, websites, organisations, reference lists and other sources searched or consulted to identify studies. Specify the date when each source was last searched or consulted.	page 04

For peer review only - http://bmjopen.bmj.com/site/about/guidelines.xhtml

Search strategy	7	Present the full search strategies for all databases, registers and websites, including any filters and limits used.	supplementary page 03-07
Selection process	8	Specify the methods used to decide whether a study met the inclusion criteria of the review, including how many reviewers screened each record and each report retrieved, whether they worked independently, and if applicable, details of automation tools used in the process.	page 05
Data collection process	9	Specify the methods used to collect data from reports, including how many reviewers collected data from each report, whether they worked independently, any processes for obtaining or confirming data from study investigators, and if applicable, details of automation tools used in the process.	page 05-06
Data items	10a	List and define all outcomes for which data were sought. Specify whether all results that were compatible with each outcome domain in each study were sought (e.g. for all measures, time points, analyses), and if not, the methods used to decide which results to collect.	page 04-05, table 1
	10b	List and define all other variables for which data were sought (e.g. participant and intervention characteristics, funding sources). Describe any assumptions made about any missing or unclear information.	page 04-05, table 1
Study risk of bias assessment	11	Specify the methods used to assess risk of bias in the included studies, including details of the tool(s) used, how many reviewers assessed each study and whether they worked independently, and if applicable, details of automation tools used in the process.	page 06
Effect measures	12	Specify for each outcome the effect measure(s) (e.g. risk ratio, mean difference) used in the synthesis or presentation of results.	NA
Synthesis methods	13a	Describe the processes used to decide which studies were eligible for each synthesis (e.g. tabulating the study intervention characteristics and comparing against the planned groups for each synthesis (item #5)).	page 06
	13b	Describe any methods required to prepare the data for presentation or synthesis, such as handling of missing summary statistics, or data conversions.	NA
	13c	Describe any methods used to tabulate or visually display results of individual studies and syntheses.	page 06

For peer review only

	13d	Describe any methods used to synthesize results and provide a rationale for the choice(s). If meta-analysis was performed, describe the model(s), method(s) to identify the presence and extent of statistical heterogeneity, and software package(s) used.	page 06
	13e	Describe any methods used to explore possible causes of heterogeneity among study results (e.g. subgroup analysis, meta-regression).	NA
	13f	Describe any sensitivity analyses conducted to assess robustness of the synthesized results.	NA
Reporting bias assessment	14	Describe any methods used to assess risk of bias due to missing results in a synthesis (arising from reporting biases).	NA
Certainty assessment	15	Describe any methods used to assess certainty (or confidence) in the body of evidence for an outcome.	page 11
**RESULTS**			
Study selection	16a	Describe the results of the search and selection process, from the number of records identified in the search to the number of studies included in the review, ideally using a flow diagram.	page 06-07
	16b	Cite studies that might appear to meet the inclusion criteria, but which were excluded, and explain why they were excluded.	page 06-07
Study characteristics	17	Cite each included study and present its characteristics.	page 08-09
Risk of bias in studies	18	Present assessments of risk of bias for each included study.	page 11, supplementary page 39-45

For peer review only - http://bmjopen.bmj.com/site/about/guidelines.xhtml

Results of individual studies	19	For all outcomes, present, for each study: (a) summary statistics for each group (where appropriate) and (b) an effect estimate and its precision (e.g. confidence/credible interval), ideally using structured tables or plots.	page 9-11
Results of syntheses	20a	For each synthesis, briefly summarise the characteristics and risk of bias among contributing studies.	page 9-11
	20b	Present results of all statistical syntheses conducted. If meta-analysis was done, present for each the summary estimate and its precision (e.g. confidence/credible interval) and measures of statistical heterogeneity. If comparing groups, describe the direction of the effect.	table 3
	20c	Present results of all investigations of possible causes of heterogeneity among study results.	NA
	20d	Present results of all sensitivity analyses conducted to assess the robustness of the synthesized results.	NA
Reporting biases	21	Present assessments of risk of bias due to missing results (arising from reporting biases) for each synthesis assessed.	NA
Certainty of evidence	22	Present assessments of certainty (or confidence) in the body of evidence for each outcome assessed.	page 11
**DISCUSSION**			
Discussion	23a	Provide a general interpretation of the results in the context of other evidence.	page 11
	23b	Discuss any limitations of the evidence included in the review.	page 11-13
	23c	Discuss any limitations of the review processes used.	page 11-13

For peer review only - http://bmjopen.bmj.com/site/about/guidelines.xhtml

	23d	Discuss implications of the results for practice, policy, and future research.	page 14-15
**OTHER INFORMATION**			
Registration and protocol	24a	Provide registration information for the review, including register name and registration number, or state that the review was not registered.	page 04
	24b	Indicate where the review protocol can be accessed, or state that a protocol was not prepared.	page 04
	24c	Describe and explain any amendments to information provided at registration or in the protocol.	NA
Support	25	Describe sources of financial or non-financial support for the review, and the role of the funders or sponsors in the review.	page 01
Competing interests	26	Declare any competing interests of review authors.	page 01
Availability of data, code and other materials	27	Report which of the following are publicly available and where they can be found: template data collection forms; data extracted from included studies; data used for all analyses; analytic code; any other materials used in the review.	page 15

NA: Not applicable

For peer review only

For peer review only - http://bmjopen.bmj.com/site/about/guidelines.xhtml

S2 Table: Search terms

S2.1 Table: Embase, Medline and Global Health (OVID)

01	chronic renal insufficiency.mp.
02	chronic kidney disease.mp.
03	chronic kidney failure.mp.
04	CKD.mp.
05	exp Renal Insufficiency, Chronic/
06	(chronic adj2 kidney adj2 disease).mp.
07	(chronic adj2 kidney adj2 failure).mp.
08	chronic renal failure.mp.
09	chronic renal disease.mp.
10	chronic kidney insufficiency.mp.
11	end stage renal disease.mp.
12	ESRD.mp.
13	kidney function.mp.
14	renal function.mp.
15	kidney dysfunction.mp.
16	renal dysfunction.mp.
17	01 or 02 or 03 or 04 or 05 or 06 or 07 or 08 or 09 or 10 or 11 or 12 or 13 or 14 or 15 or 16
18	(("Afghanistan") or ("Benin") or ("Burkina Faso") or ("Burundi") or ("Central African Republic") or ("Chad") or ("Comoros") or ("Democratic Republic of the Congo") or ("Eritrea") or ("Ethiopia") or ("Gambia") or ("Guinea") or ("Guinea-Bissau") or ("Haiti") or ("Democratic People's Republic of Korea") or ("Liberia") or ("Madagascar") or ("Malawi") or ("Mali") or ("Mozambique") or ("Nepal") or ("Niger") or ("Rwanda") or ("Senegal") or ("Sierra Leone") or ("Somalia") or ("South Sudan") or ("Tanzania") or ("Togo") or ("Uganda") or ("Zimbabwe") or ("Armenia") or ("Bangladesh") or ("Bhutan") or ("Bolivia") or ("Cape Verde") or ("Cambodia") or ("Cameroon") or ("Congo") or ("Cote d'Ivoire") or ("Djibouti") or ("Egypt") or ("El Salvador") or ("Ghana") or ("Guatemala") or ("Honduras") or ("India") or ("Indonesia") or ("Kenya") or ("Micronesia") or ("Kosovo") or ("Kyrgyzstan") or ("Laos") or ("Lesotho") or ("Mauritania") or ("Moldova") or ("Mongolia") or ("Morocco") or ("Myanmar") or ("Nicaragua") or ("Nigeria") or ("Pakistan") or ("Papua New Guinea") or ("Philippines") or ("Samoa") or ("Atlantic Islands") or ("Melanesia") or ("Sri Lanka") or ("Sudan") or ("Swaziland") or ("Syria") or ("Tajikistan") or ("Timor-Leste") or ("Tonga") or ("Tunisia") or ("Ukraine") or ("Uzbekistan") or ("Vanuatu") or ("Vietnam") or ("Middle East") or ("Yemen") or ("Zambia") or ("Albania") or ("Algeria") or ("American Samoa") or ("Angola") or ("Argentina") or ("Azerbaijan") or ("Republic of Belarus") or ("Belize") or ("Bosnia and Herzegovina") or ("Botswana") or ("Brazil") or ("Bulgaria") or ("China") or ("Colombia") or ("Costa Rica") or ("Cuba") or ("Dominica") or ("Dominican Republic") or ("Equatorial Guinea") or ("Ecuador") or ("Fiji") or ("Gabon") or ("Georgia") or ("Grenada") or ("Guyana") or ("Iran") or ("Iraq") or ("Jamaica") or ("Jordan") or ("Kazakhstan") or ("Lebanon") or ("Libya") or ("Macedonia") or ("Malaysia") or ("Indian Ocean Islands") or ("Mexico") or ("Montenegro") or ("Namibia") or ("Palau") or ("Panama") or ("Paraguay") or ("Peru") or ("Russia") or ("Serbia") or ("South Africa") or ("Saint Lucia") or ("Saint Vincent and the Grenadines") or ("Suriname") or ("Thailand") or ("Turkey") or ("Turkmenistan") or ("Venezuela") or (developing countr*) or (lowincome countr*) or (middle-income countr*) or (low-middle income countr*) or (upper-middle income countr*))
19	risk assessment.mp.
20	risk functions.mp.
21	Risk Assessment/mt
22	risk equation$.mp.
23	risk chart?.mp.
24	(risk adj3 tool$).mp.
25	risk assessment function?.mp.
26	risk assessor.mp.
27	risk appraisal$.mp.
28	risk calculation$.mp.
29	risk calculator$.mp.

For peer review only - http://bmjopen.bmj.com/site/about/guidelines.xhtml

30	risk factor$ calculator$.mp.
31	risk factor$ calculation$.mp.
32	risk engine$.mp.
33	risk equation$.mp.
34	risk table$.mp.
35	risk threshold$.mp.
36	risk disc?.mp.
37	risk disk?.mp.
38	risk scoring method?.mp.
39	scoring scheme?.mp.
40	risk scoring system?.mp.
41	risk scal$.mp.
42	risk prediction?.mp.
43	risk algorith$.mp.
44	prediction model$.mp.
45	predictive instrument?.mp.
46	project$ risk?.mp.
47	predictive model?.mp.
48	scoring method$.mp.
49	(prediction$ adj3 method$).mp.
50	exp Risk Assessment/
51	(risk? adj1 assess$).mp.
52	screening.mp.
53	diagnostic test.mp.
54	19 or 20 or 21 or 22 or 23 or 24 or 25 or 26 or 27 or 28 or 29 or 30 or 31 or 32 or 33 or 34 or 35 or 36 or 37 or 38 or 39 or 40 or 41 or 42 or 43 or 44 or 45 or 46 or 47 or 48 or 49 or 50 or 51 or 52 or 53
55	17 and 18 and 54
56	exp animals/ not humans.sh.
57	55 not 56
58	Remove duplicates from 57

For peer review

S2.2 Table: SCOPUS

((TITLE-ABS-KEY("Afghanistan") OR TITLE-ABS-KEY("Benin") OR TITLE-ABS-KEY("Burkina Faso") OR TITLE-ABS-KEY("Burundi") OR TITLE-ABS-KEY("Central African Republic") OR TITLE-ABS-KEY("Chad") OR TITLE-ABS-KEY("Comoros") OR TITLE-ABS-KEY("Democratic Republic of the Congo") OR TITLE ABSKEY("Eritrea") OR TITLE-ABS-KEY("Ethiopia") OR TITLE-ABS-KEY("Gambia") OR TITLE ABSKEY("Guinea") OR TITLE-ABS-KEY("Guinea-Bissau") OR TITLE-ABS-KEY("Haiti") OR TITLE ABSKEY("Democratic People's Republic of Korea") OR TITLE-ABS-KEY("Liberia") OR TITLE ABSKEY("Madagascar") OR TITLE-ABS-KEY("Malawi") OR TITLE-ABS-KEY("Mali") OR TITLE ABSKEY("Mozambique") OR TITLE-ABS-KEY("Nepal") OR TITLE-ABS-KEY("Niger") OR TITLE-ABS-KEY("Rwanda") OR TITLE-ABS-KEY("Senegal") OR TITLE-ABS-KEY("Sierra Leone") OR TITLE-ABSKEY("Somalia") OR TITLE-ABS-KEY("South Sudan") OR TITLE-ABS-KEY("Tanzania") OR TITLE-ABSKEY("Togo") OR TITLE-ABS-KEY("Uganda") OR TITLE-ABS-KEY("Zimbabwe") OR TITLE-ABS-KEY("Armenia") OR TITLE-ABS-KEY("Bangladesh") OR TITLE-ABS-KEY("Bhutan") OR TITLE-ABSKEY("Bolivia") OR TITLE-ABS-KEY("Cape Verde") OR TITLE-ABS-KEY("Cambodia") OR TITLE-ABSKEY("Cameroon") OR TITLE-ABS-KEY("Congo") OR TITLE-ABS-KEY("Cote d'Ivoire") OR TITLE-ABSKEY("Djibouti") OR TITLE-ABS-KEY("Bolivia") OR TITLE-ABS-KEY("Cape Verde") OR TITLE-ABS-KEY("Cambodia") OR TITLE-ABS-KEY("Cameroon") OR TITLE-ABS-KEY("Congo") OR TITLE-ABSKEY("Cote d'Ivoire") OR TITLE-ABS-KEY("Djibouti") OR TITLE-ABS-KEY("Egypt") OR TITLE-ABS-KEY("El Salvador") OR TITLE-ABS-KEY("Ghana") OR TITLE-ABS-KEY("Guatemala") OR TITLE-ABSKEY("Honduras") OR TITLE-ABS-KEY("India") OR TITLE-ABS-KEY("Indonesia") OR TITLE-ABSKEY("Kenya") OR TITLE-ABS-KEY("Micronesia") OR TITLE-ABS-KEY("Kosovo") OR TITLE-ABSKEY("Kyrgyzstan") OR TITLE-ABS-KEY("Laos") OR TITLE-ABS-KEY("Lesotho") OR TITLE-ABS-KEY("Mauritania") OR TITLE-ABS-KEY("Moldova") OR TITLE-ABS-KEY("Mongolia") OR TITLE-ABSKEY("Morocco") OR TITLE-

For peer review only - http://bmjopen.bmj.com/site/about/guidelines.xhtml

ABS-KEY("Myanmar") OR TITLE-ABS-KEY("Nicaragua") OR TITLE-ABS-KEY("Nigeria") OR TITLE-ABS-KEY("Pakistan") OR TITLE-ABS-KEY("Papua New Guinea") OR TITLE-ABSKEY("Philippines") OR TITLE-ABS-KEY("Samoa") OR TITLE-ABS-KEY("Atlantic Islands") OR TITLE-ABSKEY("Melanesia") OR TITLE-ABS-KEY("Sri Lanka") OR TITLE-ABS-KEY("Sudan") OR TITLE-ABSKEY("Swaziland") OR TITLE-ABS-KEY("Syria") OR TITLE-ABS-KEY("Tajikistan") OR TITLE-ABSKEY("Timor-Leste") OR TITLE-ABS-KEY("Tonga") OR TITLE-ABS-KEY("Tunisia") OR TITLE-ABSKEY("Ukraine") OR TITLE-ABS-KEY("Uzbekistan") OR TITLE-ABS-KEY("Vanuatu") OR TITLE-ABSKEY("Vietnam") OR TITLE-ABS-KEY("Middle East") OR TITLE-ABS-KEY("Yemen") OR TITLE-ABS-KEY("Zambia") OR TITLE-ABS-KEY("Albania") OR TITLE-ABS-KEY("Algeria") OR TITLE-ABSKEY("American Samoa") OR TITLE-ABS-KEY("Angola") OR TITLE-ABS-KEY("Argentina") OR TITLE-ABSKEY("Azerbaijan") OR TITLE-ABS-KEY("Republic of Belarus") OR TITLE-ABS-KEY("Belize") OR TITLE-ABSKEY("Bosnia and Herzegovina") OR TITLE-ABS-KEY("Botswana") OR TITLE-ABS-KEY("Brazil") OR TITLEABS-KEY("Bulgaria") OR TITLE-ABS-KEY("China") OR TITLE-ABS-KEY("Colombia") OR TITLE-ABS-KEY("Costa Rica") OR TITLE-ABS-KEY("Cuba") OR TITLE-ABS-KEY("Dominica") OR TITLE-ABSKEY("Dominican Republic") OR TITLE-ABS-KEY("Equatorial Guinea") OR TITLE-ABS-KEY("Ecuador") OR TITLE-ABS-KEY("Fiji") OR TITLE-ABS-KEY("Gabon") OR TITLE-ABS-KEY("Georgia") OR TITLE-ABSKEY("Grenada") OR TITLE-ABS-KEY("Guyana") OR TITLE-ABS-KEY("Iran") OR TITLE-ABS-KEY("Iraq") OR TITLE-ABS-KEY("Jamaica") OR TITLE-ABS-KEY("Jordan") OR TITLE-ABS-KEY("Kazakhstan") OR TITLEABS-KEY("Lebanon") OR TITLE-ABS-KEY("Libya") OR TITLE-ABS-KEY("Macedonia (Republic)") OR TITLEABS-KEY("Malaysia") OR TITLE-ABS-KEY("Indian Ocean Islands") OR TITLE-ABS-KEY("Mexico") OR TITLE-ABS-KEY("Montenegro") OR TITLE-ABS-KEY("Namibia") OR TITLE-ABS-KEY("Palau") OR TITLEABS-KEY("Panama") OR TITLE-ABS-KEY("Paraguay") OR TITLE-ABS-KEY("Peru") OR TITLE-ABSKEY("Russia") OR TITLE-ABS-KEY("Serbia") OR TITLE-ABS-KEY("South Africa") OR TITLE-ABS-KEY("Saint Lucia") OR TITLE-ABS-KEY("Saint Vincent and the Grenadines") OR TITLE-ABS-KEY("Suriname") OR TITLE-ABS-KEY("Thailand") OR TITLE-ABS-KEY("Turkey") OR TITLE-ABS-KEY("Turkmenistan") OR TITLEABS-KEY("Venezuela") OR TITLE-ABS-KEY(developing countr*) OR TITLE-ABS-KEY(lowincome countr*) OR TITLE-ABS-KEY(middle-income countr*) OR TITLE-ABS-KEY(low-middle income countr*) OR TITLEABS-KEY(upper-middle income countr*) OR TITLE-ABS-KEY(“low resource") OR TITLE-ABS-KEY ("underresourced") OR TITLE-ABS-KEY("resource poor") OR TITLE-ABS-KEY("under-developed") OR TITLE-ABSKEY("underdeveloped") OR TITLE-ABS-KEY("developing world") OR TITLE-ABS-KEY(“third world”) OR TITLE-ABS-KEY(lmic) OR TITLE-ABS-KEY(low AND middle AND income)) AND (TITLE-ABS-KEY(Risk Assessment) OR TITLE-ABS-KEY(risk? adj1 assess*) OR TITLE-ABS-KEY(risk function) OR TITLE-ABS-KEY(Risk Assessment) OR TITLE-ABS-KEY(risk functions) OR TITLE-ABS-KEY(risk equation*) OR TITLEABS-KEY(risk chart?) OR TITLE-ABS-KEY(risk adj3 tool*) OR TITLE-ABS-KEY(risk assessment function?) OR TITLE-ABS-KEY(risk assessor) OR TITLE-ABS-KEY(risk appraisal*) OR TITLE-ABS-KEY(risk calculation*) OR TITLE-ABS-KEY(risk calculator*) OR TITLE-ABS-KEY(risk factor* calculator*) OR TITLEABS-KEY(risk factor* calculation*) OR TITLE-ABS-KEY(risk engine*) OR TITLE-ABS-KEY(risk equation*) OR TITLE-ABS-KEY(risk table*) OR TITLE-ABS-KEY(risk threshold*) OR TITLE-ABS-KEY(risk disc?) OR TITLE-ABS-KEY(risk disk?) OR TITLE-ABS-KEY(risk scoring method?) OR TITLE-ABS-KEY(scoring scheme?) OR TITLE-ABS-KEY(risk scoring system?) OR TITLE-ABS-KEY(risk prediction?) OR TITLE-ABSKEY(risk algorith*) OR TITLE-ABS-KEY(prediction model*) OR TITLE-ABS-KEY(predictive instrument?) OR TITLE-ABS-KEY(project* risk?) OR TITLE-ABS-KEY(predictive model?) OR TITLE-ABS-KEY(scoring method*) OR TITLE-ABS-KEY(prediction* adj3 method*) OR TITLE-ABS-KEY(screening) OR TITLE-ABSKEY(risk scal*) OR TITLE-ABS-KEY(diagnostic test)) AND (TITLE-ABS-KEY(chronic renal insufficiency) OR TITLE-ABS-KEY(chronic kidney disease) OR TITLE-ABS-KEY(chronic kidney failure) OR TITLE-ABS-KEY(CKD) OR TITLE-ABS-KEY(chronic renal failure) OR TITLE-ABS-KEY(chronic renal disease) OR TITLE-ABS-KEY(chronic kidney insufficiency) OR TITLE-ABS-KEY(end stage renal disease) OR TITLE-ABSKEY(ESRD) OR TITLE-ABS-KEY(kidney function) OR TITLE-ABS-KEY(renal function) OR TITLE-ABSKEY(kidney dysfunction) OR TITLE-ABS-KEY(renal dysfunction) OR TITLE-ABS-KEY(chronic W/2 kidney W/2 disease) OR TITLE- ABS-KEY(chronic W/2 kidney W/2 failure) AND NOT DBCOLL(medl))

For peer review only - http://bmjopen.bmj.com/site/about/guidelines.xhtml

S2.3 Table: WEB OF SCIENCE

(((chronic renal insufficiency) OR (chronic kidney disease) OR (chronic kidney failure) OR (CKD) OR (Renal Insufficiency, Chronic) OR (chronic NEAR/2 kidney NEAR/2 disease) OR (chronic NEAR/2 kidney NEAR/2 failure) OR (chronic renal failure) OR (chronic renal disease) OR (chronic kidney insufficiency) OR (end stage renal disease) OR (ESRD) OR (kidney function) OR (renal function) OR (kidney dysfunction) OR (renal dysfunction)) AND (("Afghanistan") OR ("Benin") OR ("Burkina Faso") OR ("Burundi") OR ("Central African Republic") OR ("Chad") OR ("Comoros") OR ("Democratic Republic of the Congo") OR ("Eritrea") OR ("Ethiopia") OR ("Gambia") OR ("Guinea") OR ("Guinea-Bissau") OR ("Haiti") OR ("Democratic People's Republic of Korea") OR ("Liberia") OR ("Madagascar") OR ("Malawi") OR ("Mali") OR ("Mozambique") OR ("Nepal") OR ("Niger") OR ("Rwanda") OR ("Senegal") OR ("Sierra Leone") OR ("Somalia") OR ("South Sudan") OR ("Tanzania") OR ("Togo") OR ("Uganda") OR ("Zimbabwe") OR ("Armenia") OR ("Bangladesh") OR ("Bhutan") OR ("Bolivia") OR ("Cape Verde") OR ("Cambodia") OR ("Cameroon") OR ("Congo") OR ("Cote d'Ivoire") OR ("Djibouti") OR ("Egypt") OR ("El Salvador") OR ("Ghana") OR ("Guatemala") OR ("Honduras") OR ("India") OR ("Indonesia") OR ("Kenya") OR ("Micronesia") OR ("Kosovo") OR ("Kyrgyzstan") OR ("Laos") OR ("Lesotho") OR ("Mauritania") OR ("Moldova") OR ("Mongolia") OR ("Morocco") OR ("Myanmar") OR ("Nicaragua") OR ("Nigeria") OR ("Pakistan") OR ("Papua New Guinea") OR ("Philippines") OR ("Samoa") OR ("Atlantic Islands") OR ("Melanesia") OR ("Sri Lanka") OR ("Sudan") OR ("Swaziland") OR ("Syria") OR ("Tajikistan") OR ("Timor-Leste") OR ("Tonga") OR ("Tunisia") OR ("Ukraine") OR ("Uzbekistan") OR ("Vanuatu") OR ("Vietnam") OR ("Middle East") OR ("Yemen") OR ("Zambia") OR ("Albania") OR ("Algeria") OR ("American Samoa") OR ("Angola") OR ("Argentina") OR ("Azerbaijan") OR ("Republic of Belarus") OR ("Belize") OR ("Bosnia and Herzegovina") OR ("Botswana") OR ("Brazil") OR ("Bulgaria") OR ("China") OR ("Colombia") OR ("Costa Rica") OR ("Cuba") OR ("Dominica") OR ("Dominican Republic") OR ("Equatorial Guinea") OR ("Ecuador") OR ("Fiji") OR ("Gabon") OR ("Georgia") OR ("Grenada") OR ("Guyana") OR ("Iran") OR ("Iraq") OR ("Jamaica") OR ("Jordan") OR ("Kazakhstan") OR ("Lebanon") OR ("Libya") OR ("Macedonia (Republic) ") OR ("Malaysia") OR ("Indian Ocean Islands") OR ("Mexico") OR ("Montenegro") OR ("Namibia") OR ("Palau") OR ("Panama") OR ("Paraguay") OR ("Peru") OR ("Russia") OR ("Serbia") OR ("South Africa") OR ("Saint Lucia") OR ("Saint Vincent and the Grenadines") OR ("Suriname") OR ("Thailand") OR ("Turkey") OR ("Turkmenistan") OR ("Venezuela") OR (developing countr) OR (lowincome countr*) OR (middle-income countr*) OR (low-middle income countr*) OR (upper-middle income countr*)) AND ((risk assessment) OR (risk equation$) OR (risk chart?) OR (risk NEAR/3 tool$) OR (risk assessment function?) OR (risk assessor) OR (risk appraisal$) OR (risk calculation$) OR (risk calculator$) OR (risk factor$ calculation$) OR (risk engine$) OR (risk equation$) OR (risk table$) OR (risk threshold$) OR (risk disc?) OR (risk disk?) OR (risk scoring method?) OR (scoring scheme?) OR (risk scoring system?) OR (risk scal$) OR (risk prediction?) OR (risk algorith$) OR (prediction model$) OR (predictive instrument?) OR (project$ risk?) OR (predictive model?) OR (scoring method$) OR (prediction$ NEAR/3 method$) OR (risk? NEAR/1 assess$) OR (screening) OR (diagnostic test))) NOT ((animal*) OR ("not humans"))

For peer review only

S3 Table: **Data extraction form (by chapters)**

S3.1 Table: **Source of data and participants**

		Source of data	Participants									
N°	Study	Source of data	Participant location	Baseline year	End year (cohorts)	Sampling	Inclusion criteria	Exclusion criteria	Outcome prevalence (%)	Outcome incidence (for cohorts)	Baseline mean age	Baseline % men
1	Asgari, 2020 European Risk Assessment tool (6-years validation)	Cohort	Community	1999-2005	2011	Random	Tehran lipids and glucose study (TLGS) cohort participants.	Persons with prevalent Cardiovascular Disease (CVD), Type 2 Diabetes Mellitus or End-stage Renal Disease with (eGFR) <15 mL/min/1.73 m2. Also excluded those with missing data at baseline for creatinine (Cr), fasting plasma glucose (FPG), 2-hour postchallenge plasma	46.02 (11.95)	40.1%	58.34	29.53

For peer review only - http://bmjopen.bmj.com/site/about/guidelines.xhtml

								glucose (2 h-PCG), body mass index (BMI), waist circumference (WC) and smoking status as well as participants with missing data during follow-up on Cr, FPG, 2 h-PCG and CVD status				
1	Asgari, 2020 European Risk Assessment tool (9-years validation)	Cohort	Community	1999-2005	2009-2018	Random	Tehran lipids and glucose study (TLGS) cohort participants.	Persons with prevalent Cardiovascular Disease (CVD), Type 2 Diabetes Mellitus or End-stage Renal Disease with (eGFR) <15 mL/min/1.73 m2. Also excluded those with missing data at baseline for creatinine (Cr), fasting plasma glucose (FPG), 2-hour postchallenge plasma glucose (2 h-PCG), body mass index (BMI), waist circumference (WC) and smoking status as well as participants with missing data during follow-up on Cr, FPG, 2 h-PCG and CVD status	NI	40.6%	48.20	49.70
2	Bradshaw, 2019 - Model 1 (derivation)	Cross-sectional	Community	2015	n/a	Random	Any individual aged ≥20 years and permanently residingin at Delhi and Chennai (CARRS-II). A permanent resident was defined as a person living in the selected household, was related to the household head and ate at least 3 meals in a week with the family.	Beddriden individuals, pregnant women, participants with missing both or either serum creatinine or urine albumin-to- creatinine ratio data and participants on dialysis.	44.9 (13.5)	46.8%	48.20	49.70

For peer review only

							Households were defined as "a group of people wholive together, usually pool their income and eat atleast one meal together a day when they are at home. This does not include people who have migratedpermanently or are considered visitors"					
2	Bradshaw, 2019 - Model 2 (derivation)	Cross-sectional	Community	2015	n/a	Random	Any individual aged ≥20 years and permanently residingin at Delhi and Chennai (CARRS-II). A permanent resident was defined as a person living in the selected household, was related to the household head and ate at least 3 meals in a week with the family. Households were defined as "a group of people wholive together, usually pool their income and eat atleast one meal together a day when they are at home. This does not include people who have migratedpermanently or are considered visitors"	Beddriden individuals, pregnant women, participants with missing both or either serum creatinine or urine albumin-to- creatinine ratio data and participants on dialysis.	44.9 (13.5)	46.8%	48.20	49.70
2	Bradshaw, 2019 - Model 3a (derivation)	Cross-sectional	Community	2015	n/a	Random	Any individual aged ≥20 years and permanently residingin at Delhi and Chennai (CARRS-II). A permanent resident was defined as a person living in the selected household,	Beddriden individuals, pregnant women, participants with missing both or either serum creatinine or urine albumin-to- creatinine ratio data and participants on dialysis.	44.9 (13.5)	46.8%	39.90	46.97

For peer review only - http://bmjopen.bmj.com/site/about/guidelines.xhtml

							was related to the household head and ate at least 3 meals in a week with the family. Households were defined as "a group of people wholive together, usually pool their income and eat atleast one meal together a day when they are at home. This does not include people who have migratedpermanently or are considered visitors"					
2	Bradshaw, 2019 - Model 3b (derivation)	Cross-sectional	Community	2015	n/a	Random	Any individual aged ≥20 years and permanently residingin at Delhi and Chennai (CARRS-II). A permanent resident was defined as a person living in the selected household, was related to the household head and ate at least 3 meals in a week with the family. Households were defined as "a group of people wholive together, usually pool their income and eat atleast one meal together a day when they are at home. This does not include people who have migratedpermanently or are considered visitors"	Beddriden individuals, pregnant women, participants with missing both or either serum creatinine or urine albumin-to- creatinine ratio data and participants on dialysis.	44.9 (13.5)	46.8%	39.90	46.97
2	Bradshaw, 2019 - Model	Cross-sectional	Community	2010-2012	n/a	Random	Any individual aged ≥20 years and permanently residingin at Delhi	Beddriden individuals, pregnant women, participants with missing	NI	NI	47.20	38.00

For peer review only - http://bmjopen.bmj.com/site/about/guidelines.xhtml

	3a (CARRS-I urban validation)						(CARRS-I). A permanent resident was defined as a person living in the selected household, was related to the household head and ate at least 3 meals in a week with the family. Households were defined as "a group of people wholive together, usually pool their income and eat atleast one meal together a day when they are at home. This does not include people who have migratedpermanently or are considered visitors"	both or either serum creatinine or urine albumin-to- creatinine ratio data and participants on dialysis.				
2	Bradshaw, 2019 - Model 3a (UDAY rural validation)	Cross-sectional	Community	2014	n/a	Random	UDAY cohort participants ((a) adults aged ≥30 years residing in the sampled urban and rural areas of Sonipat and Vizag, respectively; and (b) willing to participate and provide informed consent).	Participants with missing both or either serum creatinine or urine albumin-to- creatinine ratio data, unwilling to provide informed consent, with serious chronic illnesses [such as that of the liver (cirrhosis), kidneys (renal failure) or malignancies], and pregnant women.	NI	NI	47.20	38.00
3	Carrillo-Larco, 2017 - CRONICAS-CKD (derivation	Cross-sectional	Community	2013-2014	n/a	Random	Full time resident, capable of giving informed consent, one subject per household.	Being pregnant, having active pulmonary tuberculosis, and having any disability preventing from undergoing anthropometric assessments, having CKD, missing values in the prediction variables, missing	57.7 (12.4)	49.4%		

For peer review only - http://bmjopen.bmj.com/site/about/guidelines.xhtml

	complete)							values in key variables to calculate eGFR, subjects with BMI >40 kg/m2 or BMI <18.5 kg/m2.				
3	Carrillo-Larco, 2017 - CRONICAS-CKD (derivation lab-free)	Cross-sectional	Community	2013-2014	n/a	Random	Full time resident, capable of giving informed consent, one subject per household.	Being pregnant, having active pulmonary tuberculosis, and having any disability preventing from undergoing anthropometric assessments, having CKD, missing values in the prediction variables, missing values in key variables to calculate eGFR, subjects with BMI >40 kg/m2 or BMI <18.5 kg/m2.	57.7 (12.4)	49.4%		
3	Carrillo-Larco, 2017 - CRONICAS-CKD (validation complete)	Cross-sectional	Community	2004-2006	n/a	Random	PREVENCION cohort participants.	Report having CKD, missing values in key variables to calculate eGFR, subjects with BMI >40 kg/m2 or BMI <18.5 kg/m2, age < 35 years, missing values in prediction variables.	57.1 (12.6)	47.7%		
3	Carrillo-Larco, 2017 - CRONICAS-CKD (validation lab-free)	Cross-sectional	Community	2004-2006	n/a	Random	PREVENCION cohort participants.	Report having CKD, missing values in key variables to calculate eGFR, subjects with BMI >40 kg/m2 or BMI <18.5 kg/m2, age < 35 years, missing values in prediction variables.	57.1 (12.6)	47.7%		
4	Mogueo, 2015 -	Cross-sectional	Community	2008-2011	n/a	Random	Cape Town Bellville-South study cohort participants.	Participants with missing data on all variables, except	55 (15)	23.4%		

For peer review only

	Korean model (eGFR validation)							anaemia				
4	Mogueo, 2015 - Thai model (eGFR validation)	Cross-sectional	Community	2008-2011	n/a	Random	Cape Town Bellville-South study cohort participants.	Participants with missing data on all variables, except kidney stones	55 (15)	23.4%		
4	Mogueo, 2015 - Korean model (eGFR or proteinuria validation)	Cross-sectional	Community	2008-2011	n/a	Random	Cape Town Bellville-South study cohort participants.	Participants with missing data on all variables, except anaemia	55 (15)	23.4%		
4	Mogueo, 2015 - Thai model (eGFR or proteinuria validation)	Cross-sectional	Community	2008-2011	n/a	Random	Cape Town Bellville-South study cohort participants.	Participants with missing data on all variables, except kidney stones	55 (15)	23.4%		
5	Saranburut, 2017 - Framingham Heart	Cohort	Community	2002	2012	Random	Employees of the Electric Generating Authority of Thailand (EGAT) who participated in a health survey in 2002	Subjects who had CKD at baseline or did not have serum creatinine at baseline or at follow-up.	54.6 (5.6)	70.8%		

For peer review only

	Study (MDRD validation)											
5	Saranburut, 2017 - Framingham Heart Study (CKD-EPI validation)	Cohort	Community	2002	2012	Random	Employees of the Electric Generating Authority of Thailand (EGAT) who participated in a health survey in 2003	Subjects who had CKD at baseline or did not have serum creatinine at baseline or at follow-up.	54.7 (5.7)	71.5%		
6	Saranburut, 2017 - Model 1 (derivation Clinical only)	Cohort	Community	2002-2003	2012-2013	Random	EGAT 1-2 cohort participants with preserved GFR (estimate glomerular filtration rate (eGFR) ≥ 60 mL/min/1.73m2) at baseline who attended both the examinations (EGAT 1 5rd examination and EGAT 2 4nd examination).	Patients who died, retired, moved, did not want to participate o had with missing baseline serum creatinine data. Also, patients with eGFR<60 at baseline in 2002-2003 were excluded	51.3 (7.4)	70.5%		
6	Saranburut, 2017 - Model 1 BMI (derivation Clinical only)	Cohort	Community	2002-2003	2012-2013	Random	EGAT 1-2 cohort participants with preserved GFR (estimate glomerular filtration rate (eGFR) ≥ 60 mL/min/1.73m2) at baseline who attended both the examinations (EGAT 1 5rd examination and EGAT 2 4nd examination).	Patients who died, retired, moved, did not want to participate o had with missing baseline serum creatinine data. Also, patients with eGFR<60 at baseline in 2002-2003 were excluded	51.3 (7.4)	70.5%		

For peer review only - http://bmjopen.bmj.com/site/about/guidelines.xhtml

6	Saranburut, 2017 - Model 2 (derivation Clinical + Limited laboratory tests)	Cohort	Community	2002-2003	2012-2013	Random	EGAT 1-2 cohort participants with preserved GFR (estimate glomerular filtration rate (eGFR) ≥ 60 mL/min/1.73m2) at baseline who attended both the examinations (EGAT 1 5rd examination and EGAT 2 4nd examination).	Patients who died, retired, moved, did not want to participate o had with missing baseline serum creatinine data. Also, patients with eGFR<60 at baseline in 2002-2003 were excluded	51.3 (7.4)	70.5%		
6	Saranburut, 2017 - Model 3 (derivation Clinical + Full laboratory tests)	Cohort	Community	2002-2003	2012-2013	Random	EGAT 1-2 cohort participants with preserved GFR (estimate glomerular filtration rate (eGFR) ≥ 60 mL/min/1.73m2) at baseline who attended both the examinations (EGAT 1 5rd examination and EGAT 2 4nd examination).	Patients who died, retired, moved, did not want to participate o had with missing baseline serum creatinine data. Also, patients with eGFR<60 at baseline in 2002-2003 were excluded	51.3 (7.4)	70.5%		
6	Saranburut, 2017 - Model 1 (validation Clinical only)	Cohort	Community	2009	2014	Random	EGAT 3 cohort participants with preserved GFR (eGFR ≥ 60) at baseline in 2009 (EGAT 3 1st examination) who were followed up 5 years later in 2014 (EGAT 3 2nd examination).	Participants younger than 40 years old at baseline, with missing serum creatinine values, parrients who died, retired and moved, unwilling to participate and with an eGFR <60 at baseline.	45.6 (4.2)	75.5%		
6	Saranburut, 2017 - Model 2 (validation Clinical +	Cohort	Community	2009	2014	Random	EGAT 3 cohort participants with preserved GFR (eGFR ≥ 60) at baseline in 2009 (EGAT 3 1st examination) who were followed up 5 years later in 2014 (EGAT 3 2nd examination).	Participants younger than 40 years old at baseline, with missing serum creatinine values, parrients who died, retired and moved, unwilling to participate and with an eGFR <60 at baseline.	45.6 (4.2)	75.5%		

For peer review only - http://bmjopen.bmj.com/site/about/guidelines.xhtml

	Limited laboratory tests)											
7	Thakkinstian, 2011 (derivation)	Cross-sectional	Community	2007-2008	n/a	Random	Global Screening and Early Evaluation of Kidney Disease (SEEK) study subjects: being 18 years or older, had no menstruation period for at least a week prior to the examination date if women, and whom were willing participants of the study and provided signed consent forms.	Subjects without blood or urine specimens.	45.2 (0.79)	45.5%		
8	Wen, 2020 - Simple Risk Score (derivation)	Cohort	Community	2006-2007	2012-2013	Random	Handan Eye Study (HES) participants (rural residents aged ≥30 years old living in Yongnian County).	Subjects who were diagnosed with CKD, unwilling to participate, missing follow up data (eGFR or UACR).	50 (10)	44.7%		
8	Wen, 2020 - Best-fit Risk Score (derivation)	Cohort	Community	2006-2007	2012-2013	Random	Handan Eye Study (HES) participants (rural residents aged ≥30 years old living in Yongnian County).	Subjects who were diagnosed with CKD, unwilling to participate, missing follow up data (eGFR or UACR).	50 (10)	44.7%		
9	Wu, 2016 (derivation)	Cross-sectional	Community	2012	n/a	Random	Adults older than 18 years and having given consent to this study.	Participants without: age information; body mass index (BMI) information; blood pressure (BP) measurement; serum creatinine test.	45.3 (14.3)	56.7%		
9	Wu, 2016	Cross-sectional	Community	2012	n/a	Random	Adults older than 18 years and having given consent to this study.	Participants without: age information; body mass index (BMI) information;	41.8 (11.7)	63.7%		

For peer review only - http://bmjopen.bmj.com/site/about/guidelines.xhtml

	(validation)							blood pressure (BP) measurement; serum creatinine test.				

For peer review only - http://bmjopen.bmj.com/site/about/guidelines.xhtml

S3.2 Table:: Outcome

		Outcome					
N°	Study	Outcome	Outcome details	Same outcome definition for all patients?	Blinded outcome	Predictors part of the outcome	Mean follow-up (years) (cohorts)
1	Asgari, 2020 European Risk Assessment tool (6-years validation)	CKD composite	CKD was defined as eGFR < 60 mL/min/1.73 m2, provided by the Modification of Diet in Renal Disease (MDRD).	Yes	NI	No	6.2
1	Asgari, 2020 European Risk Assessment tool (9-years validation)	CKD composite	CKD was defined as eGFR < 60 mL/min/1.73 m2, provided by the Modification of Diet in Renal Disease (MDRD).	Yes	NI	No	9.2
2	Bradshaw, 2019 - Model 1 (derivation)	CKD composite	CKD was defined as an eGFR rate <60 mL/min/1.73 m2 (estimated by the CKD-EPI equation) or UACR ≥30 mg/g	Yes	NI	No	0
2	Bradshaw, 2019 - Model 2 (derivation)	CKD composite	CKD was defined as an eGFR rate <60 mL/min/1.73 m2 (estimated by the CKD-EPI equation) or UACR ≥30 mg/g	Yes	NI	No	0
2	Bradshaw, 2019 - Model 3a (derivation)	CKD composite	CKD was defined as an eGFR rate <60 mL/min/1.73 m2 (estimated by the CKD-EPI equation) or UACR ≥30 mg/g	Yes	NI	No	0
2	Bradshaw, 2019 - Model 3b (derivation)	CKD composite	CKD was defined as an eGFR rate <60 mL/min/1.73 m2 (estimated by the CKD-EPI equation) or UACR ≥30 mg/g	Yes	NI	No	0
2	Bradshaw, 2019 - Model 3a	CKD composite	CKD was defined as an eGFR rate <60 mL/min/1.73 m2 (estimated by the CKD-EPI equation) or UACR ≥30 mg/g	Yes	NI	No	0

For peer review only - http://bmjopen.bmj.com/site/about/guidelines.xhtml

	(CARRS-I urban validation)						
2	Bradshaw, 2019 - Model 3a (UDAY rural validation)	CKD composite	CKD was defined as an eGFR rate <60 mL/min/1.73 m2 (estimated by the CKD-EPI equation) or UACR ≥30 mg/g	Yes	NI	No	0
3	Carrillo-Larco, 2017 - CRONICAS-CKD (derivation complete)	CKD composite	CKD defined as an eGFR <60 mL/min/1.73m2, using the MDRD (Modification of Diet in Renal Disease) formula, also known as CKD stage III	Yes	Yes	No	0
3	Carrillo-Larco, 2017 - CRONICAS-CKD (derivation lab-free)	CKD composite	CKD defined as an eGFR <60 mL/min/1.73m2, using the MDRD (Modification of Diet in Renal Disease) formula, also known as CKD stage III	Yes	Yes	No	0
3	Carrillo-Larco, 2017 - CRONICAS-CKD (validation complete)	CKD composite	CKD defined as an eGFR <60 mL/min/1.73m2, using the MDRD (Modification of Diet in Renal Disease) formula, also known as CKD stage III	Yes	Yes	No	0
3	Carrillo-Larco, 2017 - CRONICAS-CKD (validation lab-free)	CKD composite	CKD defined as an eGFR <60 mL/min/1.73m2, using the MDRD (Modification of Diet in Renal Disease) formula, also known as CKD stage III	Yes	Yes	No	0
4	Mogueo, 2015 - Korean model (eGFR validation)	CKD composite	eGFR <60 ml/min/1.73 m2 based on the 4-variable Modification of Diet in Renal Disease (MDRD) formula	Yes	NI	No	0
4	Mogueo, 2015 - Thai model (eGFR validation)	CKD composite	eGFR <60 ml/min/1.73 m2 based on the 4-variable Modification of Diet in Renal Disease (MDRD) formula	Yes	NI	No	0
4	Mogueo, 2015 - Korean model (eGFR or	CKD composite	eGFR <60 ml/min/1.73 m2 based on the 4-variable Modification of Diet in Renal Disease (MDRD) formula and 'any nephropathy' including any of the stages I to V of the Kidney Disease: Improving Global Outcomes Chronic	Yes	NI	No	0

For peer review only - http://bmjopen.bmj.com/site/about/guidelines.xhtml

	proteinuria validation)		Kidney Disease (KDIGO) classification				
4	Mogueo, 2015 - Thai model (eGFR or proteinuria validation)	CKD composite	eGFR <60 ml/min/1.73 m2 based on the 4-variable Modification of Diet in Renal Disease (MDRD) formula and ‘any nephropathy’ including any of the stages I to V of the Kidney Disease: Improving Global Outcomes Chronic Kidney Disease (KDIGO) classification	Yes	NI	No	0
5	Saranburut, 2017 - Framingham Heart Study (MDRD validation)	CKD composite	CKD was defined as estimate glomerular filtration rate (eGFR) <60 mL/min/1.73 m2 using the Modification of Diet in Renal Disease (MDRD)	Yes	NI	No	10
5	Saranburut, 2017 - Framingham Heart Study (CKD-EPI validation)	CKD composite	CKD defined as (eGFR) <60 mL/min/1.73 m2 using the CKD-EPI equation.	Yes	NI	No	10
6	Saranburut, 2017 - Model 1 (derivation Clinical only)	CKD composite	Preserved GFR (eGFR ≥60) at baseline and subsequently developed decreased GFR (eGFR < 60 mL/min/1.73m2) at the 10 year follow-up calculated according to two-level race variable Chronic Kidney Disease–Epidemiology Collaboration (CKDEPI) equation using the non-black coefficient. The outcome is a modification from the KDIGO definition of CKD stage 3-5	Yes	NI	No	10
6	Saranburut, 2017 - Model 1 BMI (derivation Clinical only)	CKD composite	Preserved GFR (eGFR ≥60) at baseline and subsequently developed decreased GFR (eGFR < 60 mL/min/1.73m2) at the 10 year follow-up calculated according to two-level race variable Chronic Kidney Disease–Epidemiology Collaboration (CKDEPI) equation using the non-black coefficient. The outcome is a modification from the KDIGO definition of CKD stage 3-5	Yes	NI	No	10
6	Saranburut, 2017 - Model 2 (derivation Clinical + Limited laboratory tests)	CKD composite	Preserved GFR (eGFR ≥60) at baseline and subsequently developed decreased GFR (eGFR < 60 mL/min/1.73m2) at the 10 year follow-up calculated according to two-level race variable Chronic Kidney Disease–Epidemiology Collaboration (CKDEPI) equation using the non-black	Yes	NI	No	10

For peer review only

			coefficient. The outcome is a modification from the KDIGO definition of CKD stage 3-5				
6	Saranburut, 2017 - Model 3 (derivation Clinical + Full laboratory tests)	CKD composite	Preserved GFR (eGFR ≥60) at baseline and subsequently developed decreased GFR (eGFR < 60 mL/min/1.73m2) at the 10 year follow-up calculated according to two-level race variable Chronic Kidney Disease–Epidemiology Collaboration (CKDEPI) equation using the non-black coefficient. The outcome is a modification from the KDIGO definition of CKD stage 3-5	Yes	NI	No	10
6	Saranburut, 2017 - Model 1 (validation Clinical only)	CKD composite	Preserved GFR (eGFR ≥60) at baseline and subsequently developed decreased GFR (eGFR < 60 mL/min/1.73m2) at the 10 year follow-up calculated according to two-level race variable Chronic Kidney Disease–Epidemiology Collaboration (CKDEPI) equation using the non-black coefficient. The outcome is a modification from the KDIGO definition of CKD stage 3-5	Yes	NI	No	5
6	Saranburut, 2017 - Model 2 (validation Clinical + Limited laboratory tests)	CKD composite	Preserved GFR (eGFR ≥60) at baseline and subsequently developed decreased GFR (eGFR < 60 mL/min/1.73m2) at the 10 year follow-up calculated according to two-level race variable Chronic Kidney Disease–Epidemiology Collaboration (CKDEPI) equation using the non-black coefficient. The outcome is a modification from the KDIGO definition of CKD stage 3-5	Yes	NI	No	5
7	Thakkinstian, 2011 (derivation)	CKD composite	CKD was defined as stage I & II if GFR ≥ 90 and GFR 60-89 ml/min/1.73 m2 with haematuria and/or albumin-creatinine ratio 30 mg/g or greater, stage III, IV, and V if the GFR of 30-59, 15-29, and < 15 ml/min/1.73 m2 respectively, regardless of kidney damage. eGFR was calculated using the MDRD equation for IDMS traceable serum creatinine values.	Yes	NI	No	0
8	Wen, 2020 - Simple Risk Score (derivation)	CKD composite	CKD was defined as an eGFR rate <60 mL/min/1.73 m2 ((assessed by the modified Chinese MDRD equation) or UACR ≥30 mg/g	Yes	NI	No	5.6
8	Wen, 2020 - Best-fit Risk Score (derivation)	CKD composite	CKD was defined as an eGFR rate <60 mL/min/1.73 m2 ((assessed by the modified Chinese MDRD equation) or UACR ≥30 mg/g	Yes	NI	No	5.6

For peer review only - http://bmjopen.bmj.com/site/about/guidelines.xhtml

9	Wu, 2016 (derivation)	CKD composite	Reduced eGFR was defined as eGFR<60 mL/min/1.73 m2 using the CKD-EPI equation.	Yes	NI	No	0
9	Wu, 2016 (validation)	CKD composite	Reduced eGFR was defined as eGFR<60 mL/min/1.73 m2 using the CKD-EPI equation.	Yes	NI	No	0

CKD, chronic kidney disease; CKD-EPI, chronic kidney disease epidemiology collaboration; eGFR, estimated glomerular filtration rate; GFR, glomerular filtration rate; KDIGO, MDRD, modification of diet renal disease; n/a, not applicable; NI, no information; UACR, urinary albumin-to-creatinine ratio.

For peer review only

S3.3 Table: Candidate predictors

		Candidate Predictors						
N°	Study	Number of candidate predictors	Number of predictors in the final model	Predictors timing	List of predictors in the final model	Predictors definition	Predictors ascertainment	Predictors modelling
1	Asgari, 2020 European Risk Assessment tool (6-years validation)	n/a	18	NI	Age; BMI (body mass index); waist circumference; use of antihypertensives; current smoking, parent and/or sibling with myocardial infarction or stroke; parent and/or sibling with diabetes.	Age (<45, ≥45 to <50, ≥50 to <55, ≥55 to <60, ≥60 to <65, ≥65 to <70, ≥70 to <75, ≥75 to <85); Body mass index (<25, ≥25 to <30, ≥30); Waist circumference [<94, ≥94 to <102, ≥102 (for men) and <80, ≥80 to <88, ≥88 (for women)]; use of antihypertensive medications; current smoking ('who smokes cigarettes daily or occasionally'); family history of cardiovascular disease (CVD) and/or diabetes (previously diagnosed CVD in first-degree male and female relatives aged < 55 and < 65 years, respectively)	BMI was calculated as weight (kg) divided by height (m2). Data collected by trained interviewer using a standard questionnaire	n/a
1	Asgari, 2020 European Risk Assessment tool (9-years validation)	n/a	18	NI	Age; BMI (body mass index); waist circumference; use of antihypertensives; current smoking, parent and/or sibling with myocardial infarction or stroke; parent and/or sibling with diabetes.	Age (<45, ≥45 to <50, ≥50 to <55, ≥55 to <60, ≥60 to <65, ≥65 to <70, ≥70 to <75, ≥75 to <85); Body mass index (<25, ≥25 to <30, ≥30); Waist circumference [<94, ≥94 to <102, ≥102 (for men) and <80, ≥80 to <88, ≥88 (for women)]; use of antihypertensive medications; current smoking ('who smokes cigarettes daily	BMI was calculated as weight (kg) divided by height (m2). Data collected by trained interviewer using a standard questionnaire	n/a

For peer review only - http://bmjopen.bmj.com/site/about/guidelines.xhtml

						or occasionally'); family history of cardiovascular disease (CVD) and/or diabetes (previously diagnosed CVD in first-degree male and female relatives aged < 55 and < 65 years, respectively)		
2	Bradshaw, 2019 - Model 1 (derivation)	30	NI	NI	NI	NI	NI	All continuous variables used cubic spline terms with knots placed at fixed quantiles of the predictor's marginal distribution, categorical variables were summarized using percentages and counts.
2	Bradshaw, 2019 - Model 2 (derivation)	23	NI	NI	NI	NI	NI	All continuous variables used cubic spline terms with knots placed at fixed quantiles of the predictor's marginal

For peer review only - http://bmjopen.bmj.com/site/about/guidelines.xhtml

								distribution, categorical variables were summarized using percentages and counts.
2	Bradshaw, 2019 - Model 3a (derivation)	NI	NI	NI	NI	NI	NI	All continuous variables used cubic spline terms with knots placed at fixed quantiles of the predictor's marginal distribution, categorical variables were summarized using percentages and counts.
2	Bradshaw, 2019 - Model 3b (derivation)	8	NI	NI	NI	NI	NI	All continuous variables used cubic spline terms with knots placed at fixed quantiles of the

For peer review only - http://bmjopen.bmj.com/site/about/guidelines.xhtml

								predictor's marginal distribution, categorical variables were summarized using percentages and counts.
2	Bradshaw, 2019 - Model 3a (CARRS-I urban validation)	n/a	NI	NI	NI	NI	NI	n/a
2	Bradshaw, 2019 - Model 3a (UDAY rural validation)	n/a	NI	NI	NI	NI	NI	n/a
3	Carrillo-Larco, 2017 - CRONICAS-CKD (derivation complete)	36	7	NI	Age; hypertension; anemia.	Age (< 50, 50-69, ≥ 70 years), hypertension (blood pressure ≥ 140/90 mmHg OR previous diagnosis of hypertension and currently under treatment) and anemia (haemoglobin < 13 g/dL if male and < 12 g/dL if female).	Age (information was collected by trained fieldworkers through face-to-face interviews), hypertension (blood pressure measurements were conducted according to the recommendations of the 7th Joint National Committee on the diagnosis and management of High Blood Pressure in adults (JNC-7), NI on anemia.	NI
3	Carrillo-Larco, 2017 -	26	5	NI	Age; hypertension.	Age (< 50, 50-69, ≥ 70 years), hypertension (blood pressure ≥ 140/90 mmHg OR previous diagnosis of	Age (information was collected by trained fieldworkers through face-to-	NI

For peer review only - http://bmjopen.bmj.com/site/about/guidelines.xhtml

	CRONICA S-CKD (derivation lab-free)					hypertension and currently under treatment).	face interviews), hypertension (blood pressure measurements were conducted according to the recommendations of the 7th Joint National Committee on the diagnosis and management of High Blood Pressure in adults (JNC-7), NI on anemia.	
3	Carrillo-Larco, 2017 - CRONICA S-CKD (validation complete)	n/a	7	NI	Age; hypertension; anemia.	Age (< 50, 50-69, ≥ 70 years), hypertension (blood pressure ≥ 140/90 mmHg OR previous diagnosis of hypertension and currently under treatment) and anemia (haemoglobin < 13 g/dL if male and < 12 g/dL if female).	Age (information was collected by trained fieldworkers through face-to-face interviews), hypertension (blood pressure measurements were conducted according to the recommendations of the 7th Joint National Committee on the diagnosis and management of High Blood Pressure in adults (JNC-7), NI on anemia.	n/a
3	Carrillo-Larco, 2017 - CRONICA S-CKD (validation lab-free)	n/a	5	NI	Age; hypertension.	Age (< 50, 50-69, ≥ 70 years), hypertension (blood pressure ≥ 140/90 mmHg OR previous diagnosis of hypertension and currently under treatment).	Age (information was collected by trained fieldworkers through face-to-face interviews), hypertension (blood pressure measurements were conducted according to the recommendations of the 7th Joint National Committee on the diagnosis and management of High Blood Pressure in adults (JNC-7), NI on anemia.	n/a
4	Mogueo, 2015 -	n/a	8	NI	Age; sex; diabetes mellitus; hypertension; use	Age (50-59, 60-69, ≥70); Female gender; Hypertension (history of	Participants received a standardized interview (Age	NI

For peer review only - http://bmjopen.bmj.com/site/about/guidelines.xhtml

	Korean model (eGFR validation)				of statins; proteinuria	illness, taking antihyper-tensive drug(s) or had systolic blood pressure ≥140 mmHg or diastolicblood pressure ≥90 mmHg); Diabetes (history of illness, taking oral hypoglycaemicagents or fasting plasma glucose levels≥126 mg/dL); Use of statins; Proteinuria	and sex) and physical examination during which blood pressure was measured according to the World Health Organisation (WHO) guidelines using a semi-automated digital blood pressure monitor (Rossmax PA, USA) on the right arm in the sitting position. Participants with no history of doctor diagnosed diabetes mellitus underwent a 75 g oral glucose tolerance test (OGTT) as recommended by the WHO	
4	Mogueo, 2015 - Thai model (eGFR validation)	n/a	8	NI	Age; diabetes mellitus; hypertension	Age (<40, 40-59, 60-69, >70); Hypertension (history of illness, taking antihyper-tensive drug(s) or had systolic blood pressure ≥140 mmHg or diastolicblood pressure ≥90 mmHg); Diabetes (history of illness, taking oral hypoglycaemicagents or fasting plasma glucose levels≥126 mg/dL)	Participants received a standardized interview (Age) and physical examination during which blood pressure was measured according to the World Health Organisation (WHO) guidelines using a semi-automated digital blood pressure monitor (Rossmax PA, USA) on the right arm in the sitting position. Participants with no history of doctor diagnosed diabetes mellitus underwent a 75 g oral glucose tolerance test (OGTT) as recommended by the WHO	NI
4	Mogueo, 2015 - Korean model (eGFR or	n/a	8	NI	Age; sex; diabetes mellitus; hypertension; use of statins; proteinuria	Age (50-59, 60-69, ≥70); Female gender; Hypertension (history of illness, taking antihyper-tensive drug(s) or had systolic blood pressure ≥140 mmHg or diastolicblood pressure	Participants received a standardized interview (Age and sex) and physical examination during which blood pressure was measured	NI

For peer review only - http://bmjopen.bmj.com/site/about/guidelines.xhtml

For peer review only

	proteinuria validation)					≥90 mmHg); Diabetes (history of illness, taking oral hypoglycaemicagents or fasting plasma glucose levels≥126 mg/dL); Use of statins; Proteinuria	according to the World Health Organisation (WHO) guidelines using a semi-automated digital blood pressure monitor (Rossmax PA, USA) on the right arm in the sitting position. Participants with no history of doctor diagnosed diabetes mellitus underwent a 75 g oral glucose tolerance test (OGTT) as recommended by the WHO	
4	Mogueo, 2015 - Thai model (eGFR or proteinuria validation)	n/a	8	NI	Age; diabetes mellitus; hypertension	Age (<40, 40-59, 60-69, >70); Hypertension (history of illness, taking antihyper-tensive drug(s) or had systolic blood pressure ≥140 mmHg or diastolicblood pressure ≥90 mmHg); Diabetes (history of illness, taking oral hypoglycaemicagents or fasting plasma glucose levels≥126 mg/dL)	Participants received a standardized interview (Age) and physical examination during which blood pressure was measured according to the World Health Organisation (WHO) guidelines using a semi-automated digital blood pressure monitor (Rossmax PA, USA) on the right arm in the sitting position. Participants with no history of doctor diagnosed diabetes mellitus underwent a 75 g oral glucose tolerance test (OGTT) as recommended by the WHO	NI
5	Saranburut, 2017 - Framingham Heart Study (MDRD validation)	n/a	5	NI	Diabetes mellitus; hypertension; eGFR category	Diabetes mellitus (yes); hypertension (yes); eGFR category (60-74, 75-89, 90-119)	Hypertension was defined as systolic blood pressure ≥ 140 mmHg or diastolic blood pressure ≥ 90 mmHg or use of oral antihypertensive medication. Diabetes mellitus was defined as a fasting glucose of ≥126 mg/dl or use	n/a

For peer review only - http://bmjopen.bmj.com/site/about/guidelines.xhtml

							of medications. eGFR was estimated using the Modification of Diet in Renal Disease (MDRD) equation.	
5	Saranburut, 2017 - Framingham Heart Study (CKD-EPI validation)	n/a	16	NI	Age; diabetes mellitus; hypertension; eGFR category	Age (30-34, 35-39, 40-44, 45-49, 50-54, 55-59, 60-64, 65-69, 70-74, 75-79, 80-85); diabetes mellitus (yes); hypertension (yes); eGFR category (60-74, 75-89, 90-119)	Age was obtained by a survey. Hypertension was defined as systolic blood pressure ≥ 140 mmHg or diastolic blood pressure ≥ 90 mmHg or use of oral antihypertensive medication. Diabetes mellitus was defined as a fasting glucose of ≥126 mg/dl or use of medications. eGFR was estimated using the chronic kidney disease–epidemiology collaboration (CKD-EPI) equation	n/a
6	Saranburut, 2017 - Model 1 (derivation Clinical only)	15	15	NI	Age; sex; systolic blood pressure; waist circumference; diabetes mellitus	Age (<45, 45-54, 55-59, ≥55); Sex (male, female); Waist circumference (≤80 for male or ≤90 for male, >80 for female or >90 for male); Diabetes (yes, no); Systolic blood pressure (<120, 120-129, 130-139, 140-149, 150-159, ≥160)	Age (health survey), sex (health survey). Hypertension was defined as systolic blood pressure ≥ 140 mmHg or diastolic blood pressure ≥ 90 mmHg or use of oral antihypertensive medication. Diabetes mellitus was defined as a fasting glucose of ≥126 mg/dl or a positive history of diabetes. Waist circumference was measured midway between the lowest ribs and the iliac crest.	NI
6	Saranburut, 2017 - Model 1 BMI (derivation	15	15	NI	Age; sex; systolic blood pressure; body mass index (BMI); diabetes mellitus	Age (<45, 45-54, 55-59, ≥55); Sex (male, female); BMI (<25, ≥25); Diabetes (yes, no); Systolic blood pressure (<120, 120-129, 130-139, 140-149, 150-159, ≥160)	Age (health survey), sex (health survey). Hypertension was defined as systolic blood pressure ≥ 140 mmHg or diastolic blood pressure ≥ 90 mmHg or use of oral	NI

For peer review only - http://bmjopen.bmj.com/site/about/guidelines.xhtml

	Clinical only)						antihypertensive medication. Diabetes mellitus was defined as a fasting glucose of ≥126 mg/dl or a positive history of diabetes. Body mass index was defined as weight in kilograms divided by the square of height in meters	
6	Saranburut, 2017 - Model 2 (derivation Clinical + Limited laboratory tests)	16	16	NI	Age; sex; systolic blood pressure; diabetes mellitus; glomerular filtration rate at baseline	Age (<45, 45-54, 55-59, ≥55); Sex (male, female); Diabetes (yes, no); Systolic blood pressure (<120, 120-129, 130-139, 140-149, 150-159, ≥160); eGFR (≥90, 75-89, 60-74)	Age (health survey), sex (health survey). Hypertension was defined as systolic blood pressure ≥ 140 mmHg or diastolic blood pressure ≥ 90 mmHg or use of oral antihypertensive medication. Diabetes mellitus was defined as a fasting glucose of ≥126 mg/dl or a positive history of diabetes. Serum creatinine (sCr) was measured by the enzymatic assay on the Vitros 350 analyzer (Ortho-Clinical Diagnostics, USA) using IDMS-Standard Reference Material (SRM) 967 as the standard. Estimate glomerular filtration rate (eGFR) was calculated according to two-level race variable Chronic Kidney Disease–Epidemiology Collaboration (CKDEPI) equation	NI
6	Saranburut, 2017 - Model 3 (derivation Clinical + Full	22	20	NI	Age; sex; systolic blood pressure; diabetes mellitus; glomerular filtration rate at baseline; uric acid; hemoglobin	Age (<45, 45-54, 55-59, ≥55); Sex (male, female); Diabetes (yes, no); Systolic blood pressure (<120, 120-129, 130-139, 140-149, 150-159, ≥160); eGFR (≥90, 75-89, 60-74); Uric acid (>6 for female or >7 for male, ≤6	Age (health survey), sex (health survey). Hypertension was defined as systolic blood pressure ≥ 140 mmHg or diastolic blood pressure ≥ 90 mmHg or use of oral	NI

For peer review only - http://bmjopen.bmj.com/site/about/guidelines.xhtml

	laboratory tests)					for female or ≤7 for male); Hemoglobin (<12 for female or <13 for male, ≥12 for female or ≥13 for male)	antihypertensive medication. Diabetes mellitus was defined as a fasting glucose of ≥126 mg/dl or a positive history of diabetes. Serum creatinine (sCr) was measured by the enzymatic assay on the Vitros 350 analyzer (Ortho-Clinical Diagnostics, USA) using IDMS-Standard Reference Material (SRM) 967 as the standard. Estimate glomerular filtration rate (eGFR) was calculated according to two-level race variable Chronic Kidney Disease–Epidemiology Collaboration (CKDEPI) equation. There is no information about uric acid and hemoglobin	
6	Saranburut, 2017 - Model 1 (validation Clinical only)	n/a	15	NI	Age; sex; systolic blood pressure; waist circumference; diabetes mellitus	Age (<45, 45-54, 55-59, ≥55); Sex (male, female); Waist circumference (≤80 for male or ≤90 for male, >80 for female or >90 for male); Diabetes (yes, no); Systolic blood pressure (<120, 120-129, 130-139, 140-149, 150-159, ≥160)	Age (health survey), sex (health survey). Hypertension was defined as systolic blood pressure ≥ 140 mmHg or diastolic blood pressure ≥ 90 mmHg or use of oral antihypertensive medication. Diabetes mellitus was defined as a fasting glucose of ≥126 mg/dl or a positive history of diabetes. Waist circumference was measured midway between the lowest ribs and the iliac crest.	n/a
6	Saranburut, 2017 - Model 2 (validation	n/a	16	NI	Age; sex; systolic blood pressure; diabetes mellitus; glomerular filtration rate at baseline	Age (<45, 45-54, 55-59, ≥55); Sex (male, female); Diabetes (yes, no); Systolic blood pressure (<120, 120-129, 130-139, 140-149, 150-159,	Age (health survey), sex (health survey). Hypertension was defined as systolic blood pressure ≥ 140 mmHg or	n/a

For peer review only - http://bmjopen.bmj.com/site/about/guidelines.xhtml

	Clinical + Limited laboratory tests)					≥160); eGFR (≥90, 75-89, 60-74)	diastolic blood pressure ≥ 90 mmHg or use of oral antihypertensive medication. Diabetes mellitus was defined as a fasting glucose of ≥126 mg/dl or a positive history of diabetes. Serum creatinine (sCr) was measured by the enzymatic assay on the Vitros 350 analyzer (Ortho-Clinical Diagnostics, USA) using IDMS-Standard Reference Material (SRM) 967 as the standard. Estimate glomerular filtration rate (eGFR) was calculated according to two-level race variable Chronic Kidney Disease–Epidemiology Collaboration (CKDEPI) equation	
7	Thakkinstian, 2011 (derivation)	37	10	NI	Age; history of kidney stones; diabetes mellitus; hypertension	Age (<40, 40-59, 60-69, ≥70); Hypertension (taking antihyper-tensive drug(s) or had systolic blood pressure ≥140 mmHg or diastolicblood pressure ≥90 mmHg); Diabetes (taking oral hypoglycaemicagents or fasting plasma glucose levels ≥126 mg/dL); History of kidney stone was measured by self-reporting kidney stone	Age (survey), diabetes (history of illness, relevant medicines used or laboratory tests/physical examinations), hypertension (history of illness, relevant medicines used or laboratory tests/physical examinations), and history of kidney stones (self-reported in survey).	NI
8	Wen, 2020 - Simple Risk Score (derivation)	NI	15	Time-varying	Waist circumference; systolic blood pressure; sex; education; diabetes	Waist circumference [<80/<75, 80-84.9/75-79.9, 85-89.9/80-84.9, 90-94.9/85-89.9, ≥95/≥90 (for male/female)]; systolic blood pressure (<120, 120-139, 140-159, >160); sex (male, female); education (illiterate, primary school and above); diabetes (no or yes)	During medical examinations, participants took two blood pressure measurements using a non-invasive automatic HEM-907 blood pressure monitor after 5 minutes of rest. Systolic blood pressure was identified as the	NI

For peer review only - http://bmjopen.bmj.com/site/about/guidelines.xhtml

							average values of two independent measurements; Diabetes was defined as: (1) FPG ≥7.0 mmol/L, or (2) self-reported diagnosis of diabetes, or (3) the use of antidiabetic medications; According to the number of years of education, they were divided into four groups (illiterate for 0 years, primary school for 1–6 years, junior high school for 7–9years, and senior high school for ≥10 years); Sex was self-reported; Information about waist circumference was no available	
8	Wen, 2020 - Best-fit Risk Score (derivation)	NI	19	Time-varying	Urinary Albumin-to-creatinine ratio; systolic blood pressure; C-reactive protein; triglycerides; sex; education; diabetes	Urinary Albumin-to-creatinine ratio (<5.0, 5.0-10.0, >10.0); systolic blood pressure (<120, 120-139, 140-159, >160); C-reactive protein (<1.0, 1-3, >3.0); triglycerides (<1.0, 1.0-1.7, >1.7); sex (male, female); education (illiterate, primary school and above); diabetes (no or yes)	Urinary albumin and creatinine were measured from fresh morning spot urine samples; During medical examinations, participants took two blood pressure measurements using a non-invasive automatic HEM-907 blood pressure monitor after 5 minutes of rest. Systolic blood pressure was identified as the average values of two independent measurements; Diabetes was defined as: (1) FPG ≥7.0 mmol/L, or (2) self-reported diagnosis of diabetes, or (3) the use of antidiabetic medications; According to the number of years of education, they were divided into four groups	NI

For peer review only - http://bmjopen.bmj.com/site/about/guidelines.xhtml

							(illiterate for 0 years, primary school for 1–6 years, junior high school for 7–9years, and senior high school for ≥10 years); Sex was self-reported; Information about waist circumference, C-reactive protein and triglycerides were no available	
9	Wu, 2016 (derivation)	NI	10	Baseline	Age, gender and body mass index (BMI) status.	Age (≤ 40, 41−50, 51−60, 61−70, ≥71), gender (male, female) and body mass index (BMI) status (normal, overweight: 23−24.9 kg/m2, obesity: ≥25 kg/m2).	Age (self-reported), gender (self-reported) and body mass index (BMI) status (calculated from participant's measured body weight and height).	NI
9	Wu, 2016 (validation)	n/a	10	Baseline	Age, gender and body mass index (BMI) status.	Age (≤ 40, 41 − 50, 51 − 60, 61 − 70, 71+), gender (male, female) and body mass index (BMI) status (normal, overweight: 23−24.9 kg/m2, obesity: ≥25 kg/m2).	Age (self-reported), gender (self-reported) and body mass index (BMI) status (calculated from participant's measured body weight and height).	n/a

For peer review only

S3.4 Table: Sample size and missing data

		Sample Size			Missing Data		
N°	Study	Baseline sample size	Number of outcome events	Total outcome events per candidate predictors	Missing data	Number of participants with missing data	Missing data per candidate predictors
1	Asgari, 2020 European Risk Assessment tool (6-years validation)	3270	722	n/a	Complete-case	2817	n/a
1	Asgari, 2020 European Risk Assessment tool (9-years validation)	3240	1359	n/a	Complete-case	2847	n/a
2	Bradshaw, 2019 - Model 1 (derivation)	8698	947	31,57	Complete-case	896	29,87
2	Bradshaw, 2019 - Model 2 (derivation)	8698	947	41,17	Complete-case	896	38,96
2	Bradshaw, 2019 - Model 3a (derivation)	8698	947	NI	Complete-case	896	NI
2	Bradshaw, 2019 - Model 3b (derivation)	8698	947	118,38	Complete-case	896	112,00
2	Bradshaw, 2019 - Model 3a (CARRS-I urban validation)	4065	NI	n/a	Complete-case	1300	n/a
2	Bradshaw, 2019 - Model 3a (UDAY rural validation)	4940	NI	n/a	Complete-case	1233	n/a
3	Carrillo-Larco, 2017 - CRONICAS-CKD (derivation complete)	2368	81	2,25	Complete-case	235	6,53
3	Carrillo-Larco, 2017 - CRONICAS-CKD (derivation lab-free)	2368	81	3,12	Complete-case	235	9,04
3	Carrillo-Larco, 2017 - CRONICAS-CKD (validation complete)	1459	79	n/a	Complete-case	79	n/a
3	Carrillo-Larco, 2017 - CRONICAS-CKD (validation lab-free)	1459	79	n/a	Complete-case	79	n/a
4	Mogueo, 2015 - Korean model (eGFR validation)	902	259	n/a	Complete-case	383	n/a
4	Mogueo, 2015 - Thai model (eGFR validation)	902	259	n/a	Complete-case	383	n/a
4	Mogueo, 2015 - Korean model (eGFR or proteinuria validation)	902	268	n/a	Complete-case	383	n/a
4	Mogueo, 2015 - Thai model (eGFR or proteinuria validation)	902	268	n/a	Complete-case	383	n/a
5	Saranburut, 2017 - Framingham Heart Study (MDRD validation)	2141	222	n/a	Complete-case	NI	n/a
5	Saranburut, 2017 - Framingham Heart Study (CKD-EPI validation)	2328	233	n/a	Complete-case	NI	n/a
6	Saranburut, 2017 - Model 1 (derivation Clinical only)	3186	271	18,07	Complete-case	NI	NI
6	Saranburut, 2017 - Model 1 BMI (derivation Clinical only)	3186	271	18,07	Complete-case	NI	NI
6	Saranburut, 2017 - Model 2 (derivation Clinical + Limited laboratory tests)	3186	271	16,94	Complete-case	NI	NI
6	Saranburut, 2017 - Model 3 (derivation Clinical + Full laboratory tests)	3186	271	12,32	Complete-case	NI	NI
6	Saranburut, 2017 - Model 1 (validation Clinical only)	1395	27	n/a	Complete-case	NI	NI

For peer review only - http://bmjopen.bmj.com/site/about/guidelines.xhtml

6	Saranburut, 2017 - Model 2 (validation Clinical + Limited laboratory tests)	1395	27	n/a	Complete-case	NI	NI
7	Thakkinstian, 2011 (derivation)	3459	626	16,92	NI	NI	NI
8	Wen, 2020 - Simple Risk Score (derivation)	3266	590	NI	Complete-case	992	NI
8	Wen, 2020 - Best-fit Risk Score (derivation)	3266	590	NI	Complete-case	992	NI
9	Wu, 2016 (derivation)	14374	294	NI	Complete-case	3135	NI
9	Wu, 2016 (validation)	4371	48	n/a	Complete-case	911	n/a

For peer review only

For peer review only - http://bmjopen.bmj.com/site/about/guidelines.xhtml

S3.5 Table: **Model development**

		Model Development					
N°	Study	Regression method	Were the model assumptions verified?	Predictors selection	If the prediction model was a replication, which was the original model?	If there were pre-selection, describe the method	Was a shrinkage method used?
1	Asgari, 2020 European Risk Assessment tool (6-years validation)	n/a	n/a	n/a	n/a	n/a	n/a
1	Asgari, 2020 European Risk Assessment tool (9-years validation)	n/a	n/a	n/a	n/a	n/a	n/a
2	Bradshaw, 2019 - Model 1 (derivation)	Logistic	NI	Pre-selection	n/a	Step-down selection procedure based on the Akaike information criterion to select the final predictors	No
2	Bradshaw, 2019 - Model 2 (derivation)	Logistic	NI	Pre-selection	n/a	Step-down selection procedure based on the Akaike information criterion to select the final predictors	No
2	Bradshaw, 2019 - Model 3a (derivation)	Logistic	NI	Pre-selection	n/a	Step-down selection procedure based on the Akaike information criterion to select the final predictors	No
2	Bradshaw, 2019 - Model 3b (derivation)	Logistic	NI	Pre-selection	n/a	Step-down selection procedure based on the Akaike information criterion to select the final predictors	No
2	Bradshaw, 2019 - Model 3a (CARRS-I urban validation)	n/a	n/a	n/a	n/a	n/a	n/a
2	Bradshaw, 2019 - Model 3a (UDAY rural validation)	n/a	n/a	n/a	n/a	n/a	n/a
3	Carrillo-Larco, 2017 - CRONICAS-CKD (derivation complete)	Logistic	NI	Pre-selection	n/a	Stepwise backward elimination method	No

For peer review only - http://bmjopen.bmj.com/site/about/guidelines.xhtml

3	Carrillo-Larco, 2017 - CRONICAS-CKD (derivation lab-free)	Logistic	NI	Pre-selection	n/a	Stepwise backward elimination method	No
3	Carrillo-Larco, 2017 - CRONICAS-CKD (validation complete)	n/a	n/a	n/a	n/a	n/a	n/a
3	Carrillo-Larco, 2017 - CRONICAS-CKD (validation lab-free)	n/a	n/a	n/a	n/a	n/a	n/a
4	Mogueo, 2015 - Korean model (eGFR validation)	n/a	n/a	n/a	n/a	n/a	n/a
4	Mogueo, 2015 - Thai model (eGFR validation)	n/a	n/a	n/a	n/a	n/a	n/a
4	Mogueo, 2015 - Korean model (eGFR or proteinuria validation)	n/a	n/a	n/a	n/a	n/a	n/a
4	Mogueo, 2015 - Thai model (eGFR or proteinuria validation)	n/a	n/a	n/a	n/a	n/a	n/a
5	Saranburut, 2017 - Framingham Heart Study (MDRD validation)	n/a	n/a	n/a	n/a	n/a	n/a
5	Saranburut, 2017 - Framingham Heart Study (CKD-EPI validation)	n/a	n/a	n/a	n/a	n/a	n/a
6	Saranburut, 2017 - Model 1 (derivation Clinical only)	Logistic	NI	Pre-selection	n/a	Variables were sequentially added in a pre-specified order and incorporated using a p< 0.05 threshold for entry and retention in the final model	No
6	Saranburut, 2017 - Model 1 BMI (derivation Clinical only)	Logistic	NI	Pre-selection	n/a	Variables were sequentially added in a pre-specified order and incorporated using a p< 0.05 threshold for entry and retention in the final model	No
6	Saranburut, 2017 - Model 2 (derivation Clinical + Limited laboratory tests)	Logistic	NI	Pre-selection	n/a	Variables were sequentially added in a pre-specified order and incorporated using a p< 0.05 threshold for entry and retention in the final model	No
6	Saranburut, 2017 - Model 3 (derivation Clinical + Full laboratory tests)	Logistic	NI	Pre-selection	n/a	Variables were sequentially added in a pre-specified order and incorporated using a p< 0.05 threshold for entry and retention in the final model	No

For peer review only

For peer review only - http://bmjopen.bmj.com/site/about/guidelines.xhtml

6	Saranburut, 2017 - Model 1 (validation Clinical only)	n/a	n/a	n/a	n/a	n/a	n/a
6	Saranburut, 2017 - Model 2 (validation Clinical + Limited laboratory tests)	n/a	n/a	n/a	n/a	n/a	n/a
7	Thakkinstian, 2011 (derivation)	Logistic	NI	Pre-selection	n/a	Factors with p values < 0.15 in a univariate analysis were considered to be simultaneously included in the multivariate logistic equation. Model selection was performed using F-tests, and thus only significant variables were kept in the final model. C statistic of models with and without a particular variable were then compared; if dropping that variable did not significantly reduce the explanation of the CKD, that variable was omitted in the final parsimonious model.	No
8	Wen, 2020 - Simple Risk Score (derivation)	Logistic	NI	Pre-selection	n/a	Risk factors were investigated by forward stepwise logistic regression and only statiscally significant (a two-sided P value <0.05) risk factors were retained.	No
8	Wen, 2020 - Best-fit Risk Score (derivation)	Logistic	NI	Pre-selection	n/a	Risk factors were investigated by forward stepwise logistic regression and only statiscally significant (a two-sided P value <0.05) risk factors were retained.	No
9	Wu, 2016 (derivation)	Logistic	NI	Pre-selection	n/a	Stepwise logistic regression model. Variables with a p value less than 0.1 were kept in the final model.	No
9	Wu, 2016 (validation)	n/a	n/a	n/a	n/a	n/a	n/a

For peer review only

n/a: not applicable; NI: no information

S3.6 Table: Model performance

		Model Performance				
N°	Study	Calibration	Discrimination (%)	Classification measures	Cut-off point	For replication studies, was the cut-off the same?
1	Asgari, 2020 European Risk Assessment tool (6-years validation)	Hosmer-Lemeshow X2 test (for intercept adjusted model): 13.53 with a p-value 0.09 (for male) and 10.1 with a p-value 0.26 (for women)	AUC (95% CI) for final intercept adjusted model = Male: 0.76 (0.72-0.79) and Female: 0.71 (0.69-0.73)	Men: Sensitivity = 72.7%, Specificity = 67.6%. Women: Sensitivity = 66.8%, Specificity = 65.6%.	Men: 25. Women: 19	No
1	Asgari, 2020 European Risk Assessment tool (9-years validation)	Hosmer-Lemeshow X2 test (for intercept adjusted model): 12.54 with a p-value 0.13 (for male) and 8.19 with a p-value 0.41 (for women)	AUC (95% CI) for final intercept adjusted model = Male: 0.71 (0.67-0.74) and Female: 0.70 (0.68-0.73)	Men: Sensitivity = 64.5%, Specificity = 69.5%. Women: Sensitivity = 56.9%, Specificity = 76.6%	Men: 25. Women: 23	No
2	Bradshaw, 2019 - Model 1 (derivation)	Calibration slope: 0.96	C-statistic (95% CI) = 0.79 (0.78-0.81)	Sensitivity = 72%, Specificity = 72%, PPV = 24%, NPV = 96%	0.09	n/a
2	Bradshaw, 2019 - Model 2 (derivation)	Calibration slope: 0.98	C-statistic (95% CI) = 0.73 (0.72-0.75)	Sensitivity = 68%, Specificity = 67%, PPV = 20%, NPV = 95%	0.09	n/a
2	Bradshaw, 2019 - Model 3a (derivation)	Calibration slope: 0.98	C-statistic (95% CI) = 0.77 (0.75-0.79)	Sensitivity = 71%, Specificity = 70%, PPV = 22%, NPV = 95%	0.09	n/a
2	Bradshaw, 2019 - Model 3b (derivation)	Calibration slope: 0.99	C-statistic (95% CI) = 0.77 (0.76-0.79)	Sensitivity = 71%, Specificity = 70%, PPV = 22%, NPV = 95%	0.09	n/a

For peer review only - http://bmjopen.bmj.com/site/about/guidelines.xhtml

2	Bradshaw, 2019 - Model 3a (CARRS-I urban validation)	NI	C-statistic (95% CI) = 0.74 (0.73-0.74)	NI	0.09	Yes
2	Bradshaw, 2019 - Model 3a (UDAY rural validation)	NI	C-statistic (95% CI) = 0.70 (0.69-0.71)	NI	0.09	Yes
3	Carrillo-Larco, 2017 - CRONICAS-CKD (derivation complete)	Hosmer-Lemeshow X2 test: 4.13 with a p-value of 0.53 (for final multivariable model).	AUC = 76.2%	Sensibility = 82.5%, Specificity = 70.0%, PPV = 8.8%, NPV = 99.1%, LHR+ = 2.8, LHR- = 0.3	2	n/a
3	Carrillo-Larco, 2017 - CRONICAS-CKD (derivation lab-free)	Hosmer-Lemeshow X2 test: 4.13 with a p-value of 0.53 (for final multivariable model).	AUC = 76%	Sensibility = 80.0%, Specificity = 72.0%, PPV = 9.1%, NPV = 99.0%, LHR+ = 2.9, LHR- = 0.3	2	n/a
3	Carrillo-Larco, 2017 - CRONICAS-CKD (validation complete)	NI	AUC = 70.0%.	Sensitivity = 70.5%, Specificity = 69.1%, PPV = 11.4%, NPV = 97.6%, LHR+ = 2.3, LHR- = 0.4	2	Yes
3	Carrillo-Larco, 2017 - CRONICAS-CKD (validation lab-free)	NI	AUC = 70.0%.	Sensitivity = 70.5%, Specificity = 69.7%, PPV = 11.6%, NPV = 97.7%, LHR+ = 2.3, LHR- = 0.4	2	Yes
4	Mogueo, 2015 - Korean model (eGFR validation)	Expected/Observed rate (95%) = 0.76 (0.67-0.86); Brier score = 0.164; Yates slope = 0.208	C-statistic (95% CI) = 0.797 (0.765-0.829)	Sensitivity = 82%, Specificity = 67%	0.30	NI
4	Mogueo, 2015 - Thai model (eGFR validation)	Expected/Observed rate (95%) = 0.98 (0.87-1.10); Brier score = 0.165; Yates slope = 0.200	C-statistic (95% CI) = 0.760 (0.726-0.793)	Sensitivity = 73%, Specificity = 72%	0.31	NI
4	Mogueo, 2015 - Korean model (eGFR or proteinuria validation)	Expected/Observed rate (95%) = 0.76 (0.67-0.85); Brier score = 0.161; Yates slope = 0.225	C-statistic (95% CI) = 0.811 (0.780-0.842)	Sensitivity = 84%, Specificity = 68%	0.31	NI

For peer review only - http://bmjopen.bmj.com/site/about/guidelines.xhtml

4	Mogueo, 2015 - Thai model (eGFR or proteinuria validation)	Expected/Observed rate (95%) = 0.97 (0.86-1.09); Brier score = 0.164; Yates slope = 0.211	C-statistic (95% CI) = 0.772 (0.739-0.805)	Sensitivity = 74%, Specificity = 73%	0.32	NI
5	Saranburut, 2017 - Framingham Heart Study (MDRD validation)	Hosmer-Lemeshow X2 test: 30.2 ($p<0.001$)	AUC (95% CI) = 0.69 (0.66-0.73)	NI	NI	NI
5	Saranburut, 2017 - Framingham Heart Study (CKD-EPI validation)	Hosmer-Lemeshow X2 test: 256.5 ($p<0.001$)	AUC (95% CI) = 0.63 (0.57-0.65)	NI	NI	NI
6	Saranburut, 2017 - Model 1 (derivation Clinical only)	Hosmer-Lemeshow X2 test: 9.02 ($p=0.34$)	AUC (95% CI) = 0.72 (0.69-0.75)	NI	NI	n/a
6	Saranburut, 2017 - Model 1 BMI (derivation Clinical only)	Hosmer-Lemeshow X2 test: 8.87 ($p=0.35$)	AUC (95% CI) = 0.72 (0.69-0.75)	NI	NI	n/a
6	Saranburut, 2017 - Model 2 (derivation Clinical + Limited laboratory tests)	Hosmer-Lemeshow X2 test: 10.87 ($p=0.21$)	AUC (95% CI) = 0.79 (0.76-0.82)	NI	NI	n/a
6	Saranburut, 2017 - Model 3 (derivation Clinical + Full laboratory tests)	Hosmer-Lemeshow X2 test: 8.28 ($p=0.41$)	AUC (95% CI) = 0.80 (0.77-0.82)	NI	NI	n/a
6	Saranburut, 2017 - Model 1 (validation Clinical only)	Hosmer-Lemeshow X2 test: 4.31 ($p=0.229$)	AUC (95% CI) = 0.66 (0.55-0.78)	NI	NI	NI
6	Saranburut, 2017 - Model 2 (validation Clinical + Limited laboratory tests)	Hosmer-Lemeshow X2 test: 2.29 ($p=0.514$)	AUC (95% CI) = 0.88 (0.80-0.95)	NI	NI	NI
7	Thakkinstian, 2011 (derivation)	Calibration was assessed by subtracting the two Somer's D correlation coefficients: 0.045 (95% CI: 0.034-0.057)	C-statistic of internal validation = 0.741	Sensitivity = 76%, Specificity = 69%	5	n/a

For peer review only - http://bmjopen.bmj.com/site/about/guidelines.xhtml

For peer review only

8	Wen, 2020 - Simple Risk Score (derivation)	Hosmer-Lemeshow X2 test: 4.89 (p=0.769)	AUC (95% CI) = 0.717 (0.689-0.744)	Sensitivity = 70.49%, Specificity = 65.14%, PPV = 29.8%, NPV = 91.3%, LHR+ = 2.02, LHR- = 0.45	14	n/a
8	Wen, 2020 - Best-fit Risk Score (derivation)	Hosmer-Lemeshow X2 test: 2.52 (p=0.961)	AUC (95% CI) = 0.721 (0.693-0.748)	Sensitivity = 56.83%, Specificity = 76.61%, PPV = 33.8%, NPV = 89.4%, LHR+ = 2.43, LHR- = 0.56	24	n/a
9	Wu, 2016 (derivation)	Internal validation dataset: Hosmer-Lemeshow X2 test P=0.798	AUC (95% CI) of internal validation = 0.894 (0.861-0.926)	Sensitivity = 0.820, Specificity = 0.863	36	n/a
9	Wu, 2016 (validation)	Hosmer-Lemeshow X2 test P=397	AUC = 0.880 (95%CI: 0.829-0.931)	NI	NI	NI

AUC, area under the curve; CI, confident interval; NI, no information.

For peer review only

S3.7 Table: Results

		Results			
N°	Study	Was a simplified model presented?	Were the coefficients of the regression model presented?	Was the baseline risk presented?	Were there alternative results presentation?
1	Asgari, 2020 European Risk Assessment tool (6-years validation)	No	No	Yes	No
1	Asgari, 2020 European Risk Assessment tool (9-years validation)	No	No	Yes	No
2	Bradshaw, 2019 - Model 1 (derivation)	Yes	No	No	No
2	Bradshaw, 2019 - Model 2 (derivation)	Yes	No	No	No
2	Bradshaw, 2019 - Model 3a (derivation)	No	No	No	No
2	Bradshaw, 2019 - Model 3b (derivation)	Yes	No	No	No
2	Bradshaw, 2019 - Model 3a (CARRS-I urban validation)	No	No	No	No
2	Bradshaw, 2019 - Model 3a (UDAY rural validation)	No	No	No	No
3	Carrillo-Larco, 2017 - CRONICAS-CKD (derivation complete)	Yes	Yes	No	No
3	Carrillo-Larco, 2017 - CRONICAS-CKD (derivation lab-free)	No	Yes	No	No
3	Carrillo-Larco, 2017 - CRONICAS-CKD (validation complete)	Yes	No	No	No
3	Carrillo-Larco, 2017 - CRONICAS-CKD (validation lab-free)	No	No	No	No
4	Mogueo, 2015 - Korean model (eGFR validation)	No	No	No	No
4	Mogueo, 2015 - Thai model (eGFR validation)	No	No	No	No
4	Mogueo, 2015 - Korean model (eGFR or proteinuria validation)	No	No	No	No
4	Mogueo, 2015 - Thai model (eGFR or proteinuria validation)	No	No	No	No
5	Saranburut, 2017 - Framingham Heart	No	Yes	No	No

For peer review only - http://bmjopen.bmj.com/site/about/guidelines.xhtml

	Study (MDRD validation)				
5	Saranburut, 2017 - Framingham Heart Study (CKD-EPI validation)	No	Yes	No	No
6	Saranburut, 2017 - Model 1 (derivation Clinical only)	No	Yes	No	Yes
6	Saranburut, 2017 - Model 1 BMI (derivation Clinical only)	No	No	No	Yes
6	Saranburut, 2017 - Model 2 (derivation Clinical + Limited laboratory tests)	Yes	Yes	No	Yes
6	Saranburut, 2017 - Model 3 (derivation Clinical + Full laboratory tests)	Yes	Yes	No	No
6	Saranburut, 2017 - Model 1 (validation Clinical only)	No	No	No	Yes
6	Saranburut, 2017 - Model 2 (validation Clinical + Limited laboratory tests)	Yes	No	No	Yes
7	Thakkinstian, 2011 (derivation)	No	Yes	No	Yes
8	Wen, 2020 - Simple Risk Score (derivation)	No	Yes	Yes	Yes
8	Wen, 2020 - Best-fit Risk Score (derivation)	No	Yes	Yes	Yes
9	Wu, 2016 (derivation)	No	Yes	No	Yes
9	Wu, 2016 (validation)	No	Yes	No	Yes

For peer review only

S3.8 Table: Discussion

		Discussion		
N°	Study	Interpretation of the results	Comparison with other studies in LAC	Generalizability
1	Asgari, 2020 European Risk Assessment tool (6-years validation)	Exploratory	No	Non-generalizability
1	Asgari, 2020 European Risk Assessment tool (9-years validation)	Exploratory	No	Non-generalizability
2	Bradshaw, 2019 - Model 1 (derivation)	NI	No	NI
2	Bradshaw, 2019 - Model 2 (derivation)	NI	No	NI
2	Bradshaw, 2019 - Model 3a (derivation)	Confirmatory	Yes	Non-generalizability
2	Bradshaw, 2019 - Model 3b (derivation)	NI	No	NI
2	Bradshaw, 2019 - Model 3a (CARRS-I urban validation)	Confirmatory	Yes	Non-generalizability
2	Bradshaw, 2019 - Model 3a (UDAY rural validation)	Confirmatory	Yes	Non-generalizability
3	Carrillo-Larco, 2017 - CRONICAS-CKD (derivation complete)	Exploratory	Yes	Generalizable
3	Carrillo-Larco, 2017 - CRONICAS-CKD (derivation lab-free)	Exploratory	Yes	Generalizable
3	Carrillo-Larco, 2017 - CRONICAS-CKD (validation complete)	Exploratory	Yes	Generalizable
3	Carrillo-Larco, 2017 - CRONICAS-CKD (validation lab-free)	Exploratory	Yes	Generalizable
4	Mogueo, 2015 - Korean model (eGFR validation)	Exploratory	Yes	Non-generalizability
4	Mogueo, 2015 - Thai model (eGFR validation)	Exploratory	Yes	Non-generalizability
4	Mogueo, 2015 - Korean model (eGFR or proteinuria validation)	Exploratory	Yes	Non-generalizability
4	Mogueo, 2015 - Thai model (eGFR or proteinuria validation)	Exploratory	Yes	Non-generalizability
5	Saranburut, 2017 - Framingham Heart Study (MDRD validation)	Exploratory	No	Non-generalizability
5	Saranburut, 2017 - Framingham Heart Study (CKD-EPI validation)	Exploratory	No	Non-generalizability
6	Saranburut, 2017 - Model 1 (derivation Clinical only)	Exploratory	No	Non-generalizability
6	Saranburut, 2017 - Model 1 BMI (derivation Clinical only)	Exploratory	No	Non-generalizability
6	Saranburut, 2017 - Model 2 (derivation Clinical + Limited laboratory tests)	Exploratory	No	Non-generalizability
6	Saranburut, 2017 - Model 3 (derivation Clinical + Full laboratory tests)	Exploratory	No	Non-generalizability
6	Saranburut, 2017 - Model 1 (validation Clinical only)	Exploratory	No	Non-generalizability
6	Saranburut, 2017 - Model 2 (validation Clinical + Limited laboratory tests)	Exploratory	No	Non-generalizability
7	Thakkinstian, 2011 (derivation)	Confirmatory	No	Non-generalizability

For peer review only

8	Wen, 2020 - Simple Risk Score (derivation)	Confirmatory	Yes	Non-generalizability
8	Wen, 2020 - Best-fit Risk Score (derivation)	Exploratory	Yes	Non-generalizability
9	Wu, 2016 (derivation)	Exploratory	No	Non-generalizability
9	Wu, 2016 (validation)	Exploratory	No	Non-generalizability

For peer review only

For peer review only - http://bmjopen.bmj.com/site/about/guidelines.xhtml

S4 Table: PROBAST
S4.1 Table: Risk of Bias (RoB)

Study	Participants		Predictors		
	Were appropriate data sources used, e.g., cohort, RCT, or nested case–control study data?	Were all inclusions and exclusions of participants appropriate?	Were predictors defined and assessed in a similar way for all participants?	Were predictor assessments made without knowledge of outcome data?	Are all predictors available at the time the model is intended to be used?
Asgari, 2020 European Risk Assessment tool (6-years validation)	Y	Y	Y	Y	Y
Asgari, 2020 European Risk Assessment tool (9-years validation)	Y	Y	Y	Y	Y
Bradshaw, 2019 - Model 1 (derivation)	Y	Y	Y	Y	PY
Bradshaw, 2019 - Model 2 (derivation)	Y	Y	Y	Y	Y
Bradshaw, 2019 - Model 3a (derivation)	Y	Y	Y	Y	PY
Bradshaw, 2019 - Model 3b (derivation)	Y	Y	Y	Y	PY
Bradshaw, 2019 - Model 3a (CARRS-I urban validation)	Y	Y	Y	Y	PY
Bradshaw, 2019 - Model 3a (UDAY rural validation)	Y	Y	Y	Y	PY
Carrillo-Larco, 2017 - CRONICAS-CKD (derivation complete)	Y	Y	Y	Y	PY
Carrillo-Larco, 2017 - CRONICAS-CKD (derivation lab-free)	Y	Y	Y	Y	Y
Carrillo-Larco, 2017 - CRONICAS-CKD (validation complete)	Y	Y	Y	Y	PY
Carrillo-Larco, 2017 - CRONICAS-CKD (validation lab-free)	Y	Y	Y	Y	Y
Mogueo, 2015 - Korean model (eGFR validation)	Y	Y	Y	Y	PY
Mogueo, 2015 - Thai model (eGFR validation)	Y	Y	Y	Y	Y
Mogueo, 2015 - Korean model (eGFR or proteinuria validation)	Y	Y	Y	Y	PY
Mogueo, 2015 - Thai model (eGFR or proteinuria validation)	Y	Y	Y	Y	Y

For peer review only - http://bmjopen.bmj.com/site/about/guidelines.xhtml

Saranburut, 2017 - Framingham Heart Study (MDRD validation)	Y	Y	Y	Y	PY
Saranburut, 2017 - Framingham Heart Study (CKD-EPI validation)	Y	Y	Y	Y	PY
Saranburut, 2017 - Model 1 (derivation Clinical only)	Y	Y	Y	Y	Y
Saranburut, 2017 - Model 1 BMI (derivation Clinical only)	Y	Y	Y	Y	Y
Saranburut, 2017 - Model 2 (derivation Clinical + Limited laboratory tests)	Y	Y	Y	Y	PY
Saranburut, 2017 - Model 3 (derivation Clinical + Full laboratory tests)	Y	Y	Y	Y	PY
Saranburut, 2017 - Model 1 (validation Clinical only)	Y	Y	Y	Y	Y
Saranburut, 2017 - Model 2 (validation Clinical + Limited laboratory tests)	Y	Y	Y	Y	PY
Thakkinstian, 2011 (derivation)	Y	Y	Y	Y	Y
Wen, 2020 - Simple Risk Score (derivation)	Y	Y	Y	Y	Y
Wen, 2020 - Best-fit Risk Score (derivation)	Y	Y	Y	Y	Y
Wu, 2016 (derivation)	Y	Y	Y	Y	Y
Wu, 2016 (validation)	Y	Y	Y	Y	Y

Answer options: Y (yes), PY (probably yes), N (no), PN (probably no), NI (no information), n/a (not applicable).

For peer review only

Study	Outcome					
	Was the outcome determined appropriately?	Was a prespecified or standard outcome definition used?	Were predictors excluded from the outcome definition?	Was the outcome defined and determined in a similar way for all participants?	Was the outcome determined without knowledge of predictor information?	Was the time interval between predictor assessment and outcome determination appropriate?
Asgari, 2020 European Risk Assessment tool (6-years validation)	Y	Y	Y	Y	NI	Y
Asgari, 2020 European Risk Assessment tool (9-years validation)	Y	Y	Y	Y	NI	Y
Bradshaw, 2019 - Model 1 (derivation)	Y	Y	Y	Y	NI	PY
Bradshaw, 2019 - Model 2 (derivation)	Y	Y	Y	Y	NI	Y
Bradshaw, 2019 - Model 3a (derivation)	NI	Y	Y	Y	NI	PY
Bradshaw, 2019 - Model 3b (derivation)	Y	Y	Y	Y	NI	PY
Bradshaw, 2019 - Model 3a (CARRS-I urban validation)	Y	Y	Y	Y	NI	PY
Bradshaw, 2019 - Model 3a (UDAY rural validation)	Y	Y	Y	Y	NI	PY
Carrillo-Larco, 2017 - CRONICAS-CKD (derivation complete)	Y	Y	Y	Y	PY	PY
Carrillo-Larco, 2017 - CRONICAS-CKD (derivation lab-free)	Y	Y	Y	Y	PY	Y
Carrillo-Larco, 2017 - CRONICAS-CKD (validation complete)	Y	Y	Y	Y	PY	PY

For peer review only

Carrillo-Larco, 2017 - CRONICAS-CKD (validation lab-free)	Y	Y	Y	Y	PY	Y
Mogueo, 2015 - Korean model (eGFR validation)	Y	Y	Y	Y	NI	PY
Mogueo, 2015 - Thai model (eGFR validation)	Y	Y	Y	Y	NI	Y
Mogueo, 2015 - Korean model (eGFR or proteinuria validation)	Y	Y	Y	Y	NI	PY
Mogueo, 2015 - Thai model (eGFR or proteinuria validation)	Y	Y	Y	Y	NI	Y
Saranburut, 2017 - Framingham Heart Study (MDRD validation)	Y	Y	Y	Y	NI	PY
Saranburut, 2017 - Framingham Heart Study (CKD-EPI validation)	Y	Y	Y	Y	NI	PY
Saranburut, 2017 - Model 1 (derivation Clinical only)	Y	Y	Y	Y	NI	Y
Saranburut, 2017 - Model 1 BMI (derivation Clinical only)	Y	Y	Y	Y	NI	Y
Saranburut, 2017 - Model 2 (derivation Clinical + Limited laboratory tests)	Y	Y	Y	Y	NI	PY
Saranburut, 2017 - Model 3 (derivation Clinical + Full laboratory tests)	Y	Y	Y	Y	NI	PY
Saranburut, 2017 - Model 1 (validation Clinical only)	Y	Y	Y	Y	NI	Y
Saranburut, 2017 - Model 2 (validation Clinical + Limited laboratory tests)	Y	Y	Y	Y	NI	PY
Thakkinstian, 2011 (derivation)	Y	Y	Y	Y	NI	Y
Wen, 2020 - Simple Risk Score (derivation)	Y	Y	Y	Y	NI	Y

For peer review only

For peer review only - http://bmjopen.bmj.com/site/about/guidelines.xhtml

Wen, 2020 - Best-fit Risk Score (derivation)	Y	Y	Y	Y	NI	Y
Wu, 2016 (derivation)	Y	Y	Y	Y	NI	Y
Wu, 2016 (validation)	Y	Y	Y	Y	NI	Y

Answer options: Y (yes), PY (probably yes), N (no), PN (probably no), NI (no information), n/a (not applicable).

For peer review only

For peer review only - http://bmjopen.bmj.com/site/about/guidelines.xhtml

Study	Analysis								
	Were there a reasonable number of participants with the outcome?	Were continuous and categorical predictors handled appropriately?	Were all enrolled participants included in the analysis?	Were participants with missing data handled appropriately?	Was selection of predictors based on univariable analysis avoided? [development studies only]	Were complexities in the data (e.g., censoring, competing risks, sampling of control participants) accounted for appropriately?	Were relevant model performance measures evaluated appropriately?	Were model overfitting and optimism in model performance accounted for? [development studies only]	Do predictors and their assigned weights in the final model correspond to the results from the reported multivariable analysis? [development studies only]
Asgari, 2020 European Risk Assessment tool (6-years validation)	Y	Y	N	N	n/a	NI	N	n/a	n/a
Asgari, 2020 European Risk Assessment tool (9-years validation)	Y	Y	N	N	n/a	NI	N	n/a	n/a
Bradshaw, 2019 - Model 1 (derivation)	Y	N	N	N	N	NI	Y	Y	NI
Bradshaw, 2019 - Model 2 (derivation)	Y	N	N	N	N	NI	Y	Y	NI
Bradshaw, 2019 - Model 3a (derivation)	NI	NI	N	N	N	NI	Y	Y	NI
Bradshaw, 2019 - Model 3b (derivation)	Y	N	N	N	N	NI	Y	Y	NI
Bradshaw, 2019 - Model 3a (CARRS-I urban validation)	NI	Y	N	N	n/a	NI	NI	n/a	n/a

For peer review only - http://bmjopen.bmj.com/site/about/guidelines.xhtml

Bradshaw, 2019 - Model 3a (UDAY rural validation)	NI	Y	N	N	n/a	NI	NI	n/a	n/a
Carrillo-Larco, 2017 - CRONICAS-CKD (derivation complete)	N	N	N	N	N	NI	N	Y	Y
Carrillo-Larco, 2017 - CRONICAS-CKD (derivation lab-free)	N	N	N	N	N	NI	N	Y	Y
Carrillo-Larco, 2017 - CRONICAS-CKD (validation complete)	N	Y	N	N	n/a	NI	N	n/a	n/a
Carrillo-Larco, 2017 - CRONICAS-CKD (validation lab-free)	N	Y	N	N	n/a	NI	N	n/a	n/a
Mogueo, 2015 - Korean model (eGFR validation)	Y	Y	N	N	n/a	NI	PY	n/a	n/a
Mogueo, 2015 - Thai model (eGFR validation)	Y	Y	N	N	n/a	NI	PY	n/a	n/a
Mogueo, 2015 - Korean model (eGFR or proteinuria validation)	Y	Y	N	N	n/a	NI	PY	n/a	n/a
Mogueo, 2015 - Thai model (eGFR or proteinuria validation)	Y	Y	N	N	n/a	NI	PY	n/a	n/a
Saranburut, 2017 - Framingham Heart Study (MDRD validation)	Y	Y	N	N	n/a	NI	N	n/a	n/a
Saranburut, 2017 - Framingham Heart Study (CKD-EPI validation)	Y	Y	N	N	n/a	NI	N	n/a	n/a
Saranburut, 2017 - Model 1 (derivation Clinical only)	PY	N	N	N	N	NI	N	Y	Y
Saranburut, 2017 - Model 1 BMI (derivation Clinical only)	PY	N	N	N	N	NI	N	Y	NI

For peer review only - http://bmjopen.bmj.com/site/about/guidelines.xhtml

Saranburut, 2017 - Model 2 (derivation Clinical + Limited laboratory tests)	PY	N	N	N	N	NI	N	Y	Y
Saranburut, 2017 - Model 3 (derivation Clinical + Full laboratory tests)	PN	N	N	N	N	NI	N	Y	Y
Saranburut, 2017 - Model 1 (validation Clinical only)	N	Y	N	N	n/a	NI	N	n/a	n/a
Saranburut, 2017 - Model 2 (validation Clinical + Limited laboratory tests)	N	Y	N	N	n/a	NI	N	n/a	n/a
Thakkinstian, 2011 (derivation)	PY	N	NI	NI	N	NI	N	Y	Y
Wen, 2020 - Simple Risk Score (derivation)	NI	N	N	N	N	NI	N	N	Y
Wen, 2020 - Best-fit Risk Score (derivation)	NI	N	N	N	N	NI	N	N	Y
Wu, 2016 (derivation)	NI	N	N	N	N	NI	N	N	Y
Wu, 2016 (validation)	N	Y	N	N	n/a	NI	N	n/a	Y

Answer options: Y (yes), PY (probably yes), N (no), PN (probably no), NI (no information), n/a (not applicable).

For peer review only

S4.2 Table: Applicability

N°	Study	Participants	Predictors	Outcome
1	Asgari, 2020 European Risk Assessment tool (6-years validation)	Low	Low	Low
1	Asgari, 2020 European Risk Assessment tool (9-years validation)	Low	Low	Low
2	Bradshaw, 2019 - Model 1 (derivation)	Low	Low	Low
2	Bradshaw, 2019 - Model 2 (derivation)	Low	Low	Low
2	Bradshaw, 2019 - Model 3a (derivation)	Low	Low	Low
2	Bradshaw, 2019 - Model 3b (derivation)	Low	Low	Low
2	Bradshaw, 2019 - Model 3a (CARRS-I urban validation)	Low	Low	Low
2	Bradshaw, 2019 - Model 3a (UDAY rural validation)	Low	Low	Low
3	Carrillo-Larco, 2017 - CRONICAS-CKD (derivation complete)	Low	Low	Low
3	Carrillo-Larco, 2017 - CRONICAS-CKD (derivation lab-free)	Low	Low	Low
3	Carrillo-Larco, 2017 - CRONICAS-CKD (validation complete)	Low	Low	Low
3	Carrillo-Larco, 2017 - CRONICAS-CKD (validation lab-free)	Low	Low	Low
4	Mogueo, 2015 - Korean model (eGFR validation)	Low	Low	Low
4	Mogueo, 2015 - Thai model (eGFR validation)	Low	Low	Low
4	Mogueo, 2015 - Korean model (eGFR or proteinuria validation)	Low	Low	Low
4	Mogueo, 2015 - Thai model (eGFR or proteinuria validation)	Low	Low	Low
5	Saranburut, 2017 - Framingham Heart Study (MDRD validation)	Low	Low	Low
5	Saranburut, 2017 - Framingham Heart Study (CKD-EPI validation)	Low	Low	Low
6	Saranburut, 2017 - Model 1 (derivation Clinical only)	Low	Low	Low
6	Saranburut, 2017 - Model 1 BMI (derivation Clinical only)	Low	Low	Low
6	Saranburut, 2017 - Model 2 (derivation Clinical + Limited laboratory tests)	Low	Low	Low
6	Saranburut, 2017 - Model 3 (derivation Clinical + Full laboratory tests)	Low	Low	Low
6	Saranburut, 2017 - Model 1 (validation Clinical only)	Low	Low	Low
6	Saranburut, 2017 - Model 2 (validation Clinical + Limited laboratory tests)	Low	Low	Low
7	Thakkinstian, 2011 (derivation)	Low	Low	Low
8	Wen, 2020 - Simple Risk Score (derivation)	Low	Low	Low
8	Wen, 2020 - Best-fit Risk Score (derivation)	Low	Low	Low
9	Wu, 2016 (derivation)	Low	Low	Low

For peer review only - http://bmjopen.bmj.com/site/about/guidelines.xhtml

9	Wu, 2016 (validation)	Low	Low	Low

Answer options: Low (low concern for applicability), Hig (High concern for applicability) and Unclear (Unclear concern for applicability)

For peer review only

For peer review only - http://bmjopen.bmj.com/site/about/guidelines.xhtml

S1 Figure: Countries where studies were conducted.

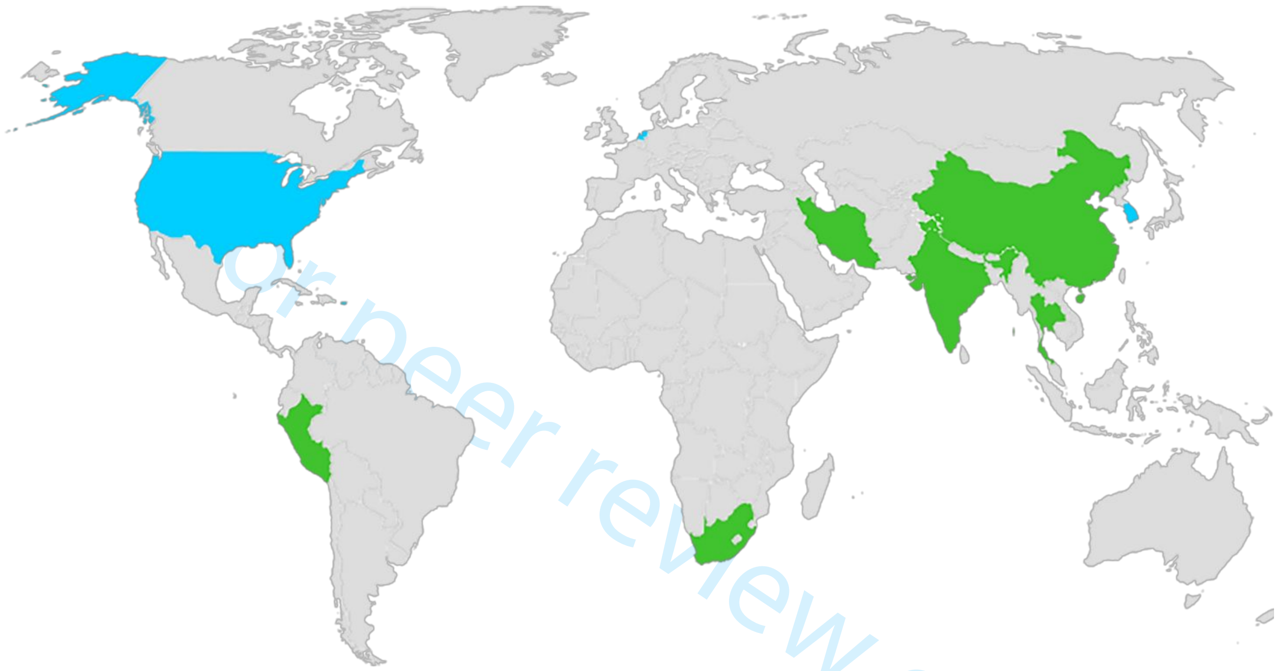

LMIC that developed and/or validated models included in this review (Green). Moreover, Asgari et al, Mogueo et al] and Saranburut et al validated risk models that were originally derivated in the Netherlands, South Korea and the United States, respectively (Blue).

For peer review only

S2 Figure: Predictors included in the final models.

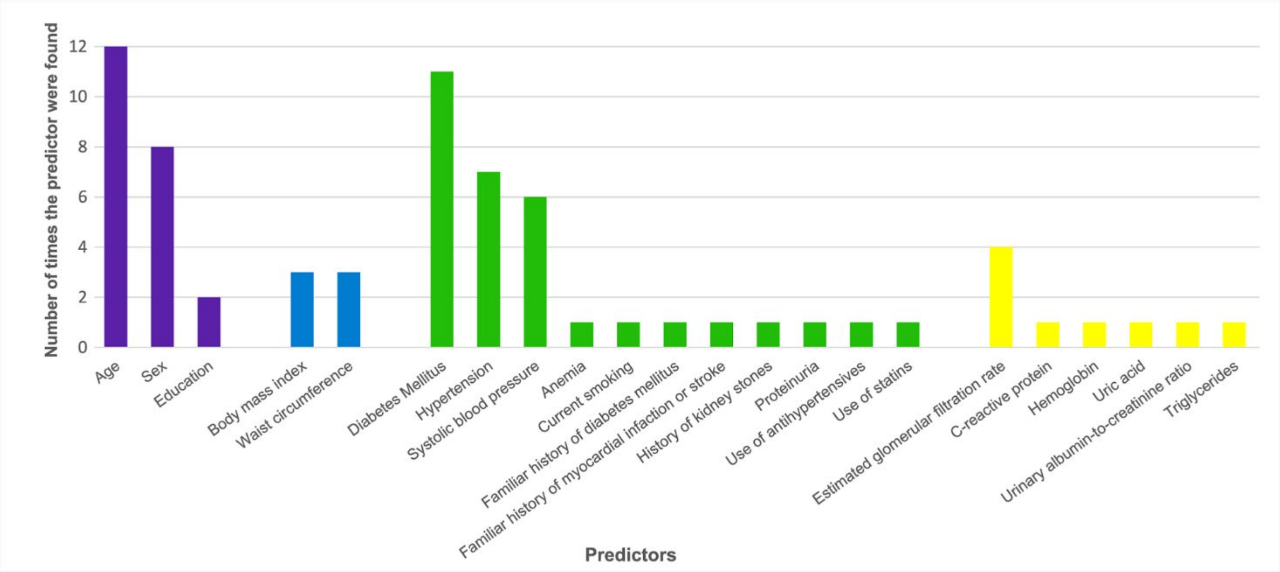

The colours of the bars identify the underlying characteristic of predictors inherent to: the subject (purple), anthropometrics (blue), clinical assessment and history (green), and laboratory measures (yellow).

For peer review only - http://bmjopen.bmj.com/site/about/guidelines.xhtml

BMJ Open

A systematic review of diagnostic and prognostic models of chronic kidney disease in low- and middle- income countries

Journal:	*BMJ Open*
Manuscript ID	bmjopen-2021-058921.R1
Article Type:	Original research
Date Submitted by the Author:	21-Feb-2022
Complete List of Authors:	Aparcana-Granda, Diego J.; School of Medicine 'Alberto Hurtado', Universidad Peruana Cayetano Heredia, Lima, Peru; CRONICAS Centre of Excellence in Chronic Diseases, Universidad Peruana Cayetano Heredia, Lima, Peru<br>Ascencio, Edson J.; School of Medicine 'Alberto Hurtado', Universidad Peruana Cayetano Heredia, Lima, Peru; Health Innovation Laboratory, Institute of Tropical Medicine 'Alexander von Humboldt', Universidad Peruana Cayetano Heredia, Lima, Peru<br>Carrillo Larco, Rodrigo M. ; Imperial College London,
**Primary Subject Heading**:	Epidemiology
Secondary Subject Heading:	Epidemiology, Global health, Renal medicine, Public health
Keywords:	Chronic renal failure < NEPHROLOGY, EPIDEMIOLOGY, PUBLIC HEALTH, NEPHROLOGY

SCHOLARONE™
Manuscripts

I, the Submitting Author has the right to grant and does grant on behalf of all authors of the Work (as defined in the below author licence), an exclusive licence and/or a non-exclusive licence for contributions from authors who are: i) UK Crown employees; ii) where BMJ has agreed a CC-BY licence shall apply, and/or iii) in accordance with the terms applicable for US Federal Government officers or employees acting as part of their official duties; on a worldwide, perpetual, irrevocable, royalty-free basis to BMJ Publishing Group Ltd ("BMJ") its licensees and where the relevant Journal is co-owned by BMJ to the co-owners of the Journal, to publish the Work in this journal and any other BMJ products and to exploit all rights, as set out in our licence.

The Submitting Author accepts and understands that any supply made under these terms is made by BMJ to the Submitting Author unless you are acting as an employee on behalf of your employer or a postgraduate student of an affiliated institution which is paying any applicable article publishing charge ("APC") for Open Access articles. Where the Submitting Author wishes to make the Work available on an Open Access basis (and intends to pay the relevant APC), the terms of reuse of such Open Access shall be governed by a Creative Commons licence – details of these licences and which Creative Commons licence will apply to this Work are set out in our licence referred to above.

Other than as permitted in any relevant BMJ Author's Self Archiving Policies, I confirm this Work has not been accepted for publication elsewhere, is not being considered for publication elsewhere and does not duplicate material already published. I confirm all authors consent to publication of this Work and authorise the granting of this licence.

For peer review only

A systematic review of diagnostic and prognostic models of chronic kidney disease in low- and middle- income countries

Diego J. Aparcana-Granda[1,2¶], Edson J. Ascencio[1,3,4¶], Rodrigo M. Carrillo-Larco[2,5¶*]

[1] School of Medicine 'Alberto Hurtado', Universidad Peruana Cayetano Heredia, Lima, Peru

[2] CRONICAS Centre of Excellence in Chronic Diseases, Universidad Peruana Cayetano Heredia, Lima, Peru

[3] Emerge, Emerging Diseases and Climate Change Research Unit, School of Public Health and Administration, Universidad Peruana Cayetano Heredia, Lima, Peru

[4] Health Innovation Laboratory, Institute of Tropical Medicine 'Alexander von Humboldt', Universidad Peruana Cayetano Heredia, Lima, Peru

[5] Department of Epidemiology and Biostatistics, School of Public Health, Imperial College London, London, UK

*Corresponding author (RMCL)

Email: rcarrill@ic.ac.uk

¶ These authors contributed equally to this work.

For peer review only

For peer review only - http://bmjopen.bmj.com/site/about/guidelines.xhtml

ABSTRACT

Objective: To summarize available chronic kidney disease (CKD) diagnostic and prognostic models in Low- and Middle-Income countries (LMIC)

Method: Systematic review (PRISMA guidelines). We searched Medline, EMBASE, Global Health (these three through OVID), Scopus and Web of Science from inception to April 9th, 2021, April 17th, 2021 and April 18th, 2021, respectively . We first screened titles and abstracts, and then studied in detail the selected reports; both phases were conducted by two reviewers independently. We followed the CHARMS recommendations and used the PROBAST for risk of bias assessment.

Results: The search retrieved 14,845 results, 11 reports were studied in detail and nine (n= 61,134) were included in the qualitative analysis. The proportion of women in the study population varied between 24.5%-76.6%, and the mean age ranged between 41.8-57.7 years. Prevalence of undiagnosed chronic kidney disease ranged between 1.1%-29.7%. Age, diabetes mellitus and sex were the most common predictors in the diagnostic and prognostic models. Outcome definition varied greatly, mostly consisting of urinary albumin-to-creatinine ratio and estimated glomerular filtration rate. The highest performance metric was the negative predictive value. All studies exhibited high risk of bias, and some had methodological limitations.

Conclusion: There is no strong evidence to support the use of a CKD diagnostic or prognostic model throughout LMIC. The development, validation and implementation of risk scores must be a research and public health priority in LMIC to enhance CKD screening to improve timely diagnosis.

Keywords: population health; prognosis research; non-communicable diseases

For peer review only

For peer review only - http://bmjopen.bmj.com/site/about/guidelines.xhtml

Strengths and limitations of this study

Strengths

- An extensive search was conducted, involving five major databases (Medline, Embase, Global Health, Scopus and Web of Science).
- A comprehensive list of available CKD diagnostic and prognostic models and their limitations is provided, which were not previously accounted for in the LMIC population.
- This study adhered to PRISMA, CHARMS and PROBAST guidelines.

Limitations

- Meta-analysis was not possible due to the heterogeneity in the measurement of outcomes.
- Additional data sources such as grey literature were not retrieved.

For peer review only

For peer review only - http://bmjopen.bmj.com/site/about/guidelines.xhtml

59 **INTRODUCTION**

Chronic kidney disease (CKD) is a condition with a large burden globally. Between 1990 and 2017, the health metrics of CKD showed a bleak profile: mortality, incidence and kidney transplantation rates increased by 3%, 29% and 34%, respectively.[1] CKD led to 1.2 million deaths in 2017 and in the best-case scenario, CKD mortality will increase to 2.2 million deaths and become the 5th cause of years of life lost (YLL) by 2040.[2] CKD reveals disparities between low- and middle-income countries (LMIC) and high-income countries (HIC). In the period 1990-2016, the age-standardised disability-adjusted life-years (DALY) due to CKD was the highest in LMIC,[3] where they need to optimize CKD early diagnosis.

Risk scores are a cost-effective alternative for CKD screening and early diagnosis.[4] These equations require less resources and contribute to decision making,[5] and allow screening of large populations.[4] Many of the available CKD risk scores have been developed in HIC,[6-8] and they may not be used in LMIC without recalibration to secure accurate predictions. How many CKD risk scores there are for LMIC, and what their strengths and limitations are, remains largely unknown.[9 10] This limits our knowledge of what tools there are to enhance CKD screening in LMIC. Similarly, this lack of evidence prevents planning research to overcome the limitations of available models. To fill these gaps and to inform CKD screening strategies in LMIC, we summarized available CKD diagnostic and prognostic models in LMIC.

METHODS

Protocol and registration

This systematic review and critical appraisal of the scientific literature was conducted following the Preferred Reporting Items for Systematic Reviews and Meta-Analysis guidelines (PRISMA) statement[11] (S1 Table). Protocol is available elsewhere[12] and in the S1 Text. We followed the CHecklist for critical Appraisal and data extraction for systematic Reviews of prediction Modelling Studies (CHARMS) guidelines.[13 14]

Information sources

We searched Medline, EMBASE, Global Health (these three through OVID), Scopus and Web of Science from inception to April 9th, 2021, April 17th, 2021 and April 18th, 2021, respectively. The

For peer review only - http://bmjopen.bmj.com/site/about/guidelines.xhtml

search strategy is available in S2 Table. We also screened the references of relevant systemic reviews[10] and of the selected studies.

Eligibility criteria

We sought models which assessed the current CKD status (i.e., diagnostic) or future CKD risk (i.e., prognostic), aiming to inform physicians, researchers, and the general population (Table 1). Reports could include model derivation, external validation, or both. The target population was adults (≥18 years) in LMIC according to The World Bank.[15]

Study selection

Reports were selected if the study population included people who were from and currently living in LMIC. Cross-sectional (diagnostic models) and longitudinal studies (prognostic models) with a random sample of the general population were included. The outcome was CKD based on a laboratory or imaging test (isolated or in combination with self-reported diagnosis): urine albumin-creatinine ratio, urine protein-creatinine ratio, albumin excretion ratio, urine sediment, kidney images, kidney biopsy or the estimated glomerular filtration rate (eGFR).[12]

Reports had to present the development and/or validation of a multivariable model. On the other hand, reports with LMIC populations outside LMIC, or those including foreigners living in LMIC, were excluded. Reports that only studied people with underlying conditions (e.g., patients with diabetes), people with a specific risk factor (e.g., alcohol consumption), or a hospital-based population, were excluded. We also excluded models that were developed using machine learning techniques due to their usually poor report of performance metrics, as noted from previous reviews.[16,17] To overcome this limitation, CHARMS and PROBAST tools are currently being adapted to machine learning methodology but are yet to be published.[18]

Data collation

We used EndNote20 and Rayyan[19] to remove duplicates from the search results. We used Rayyan[19] to screen titles and abstracts by two reviewers independently (DJA-G and EJA); discrepancies were solved by consensus. Two reviewers independently (DJA-G and EJA) studied the full length of the reports selected in the screening phase; discrepancies were solved by consensus. If consensus was not reached, a third party was consulted (RMC-L). A data extraction form based on the CHARMS guidelines[14] was developed and not modified during data collation. Data was extracted as presented

For peer review only - http://bmjopen.bmj.com/site/about/guidelines.xhtml

in the original reports by two reviewers independently (DJA-G and EJA); discrepancies were solved by consensus.

Risk of bias of individual studies

We used the PROBAST (Prediction model Risk of Bias ASsessment Tool) to assess the risk of bias of diagnostic and prognostic models.[20 21] Two reviewers (EJA and DJA-G) independently ascertained the risk of bias of individual reports; discrepancies were solved by consensus or a third party (RMC-L).

Synthesis of results

A qualitative synthesis was conducted whereby the characteristics of the selected models was comprehensively described.[12] Quantitative analysis (meta-analysis) was not conducted because the selected models used different predictors and they had different outcome definitions.

Ethics

This review was deemed as a low risk because human subjects were not directly involved. The funder did not have any role in the conception, conduction, results interpretation, and drafting of this work. Results and opinions expressed in the article are entirely the authors.

Patient and public involvement

No patient involved.

RESULTS

Reports selection

The search yielded 14,845 reports. After removing duplicates (1,462 articles), we screened 13,383 titles and abstracts. Then, 11 reports were selected, one of them was not available as full-text,[22] and the rest (10 articles) were studied in detail. We excluded one report because the study population was not randomly selected,[23] and another report because it was conducted in a HIC.[24] Additionally, one report was identified by reference searching.[25] Finally, nine reports (n=61,134) were included in the qualitative synthesis (Figure 1).

General characteristics of the selected reports

Original reports were from Iran,[26] India,[27] Peru, [28] South Africa, [25] two from China[29 30] and three from Thailand[31-33] (S1 Figure). All studies were developed on community-based populations with random sampling (S3 Table).

For peer review only

For peer review only - http://bmjopen.bmj.com/site/about/guidelines.xhtml

Overall, Wu and colleagues studied the largest sample size (n=14,374) which was a population of workers who underwent health checks;[30] conversely, the smallest sample was studied by Mogueo *et al* (n=902).[25] The oldest data was collected in 1999[26] whereas the most recent study was published in 2018.[26]

The sample size analysed to derive the diagnostic models ranged from 2,368[28] to 14,374 people,[30] and from 902[25] to 4,940[27] for the validation models. The mean age of participants in the derivation models varied from 44.9 to 57.7 years, and the proportion of male subjects ranged from 46.8% to 70.5%.[27-30 32 33] The mean age of participants in the validation models varied from 41.8 to 57.1 years, and the proportion of male subjects ranged from 23.4% to 75.5%[25-28 30-32] (Table 2; S3 Table).

The number of CKD cases varied greatly in the derivation models, from 81[28] to 947;[27] the corresponding numbers in the validation models were 27[32] and 1,359[26]. Of note, number of CKD cases could not be extracted from the validation work by Bradshaw *et al*[27]. The ratio of outcome events per number of candidate predictors in the derivation models ranged from 2.3[28] to 135.3[27]. This ratio could not be calculated for the derivation models by Wen *et al*[29] and Wu *et al*[30]. Across all reports, missing data were handled by conducting a complete-case analysis;[25-32] this information was not available in the study by Thakkinstian's *et al*[33] (Table 2; S3 Table).

What has been done?

In 2011, Thakkinstian *et al* derived one model using cross-sectional data.[33] In 2015, Mogueo *et al* used cross-sectional data to validate two models that were previously developed in South Korea and Thailand using two different outcome definitions for each model, i.e., they provided estimates for four model validations.[25] In 2016, Wu *et al* used cross-sectional data to derive and validate one model, i.e., they provided estimates for two models (one derivation and one validation).[30] In 2017, Carrillo-Larco *et al* used cross-sectional data to derive and validate two models, i.e., they provided estimates for four models (two derivations and two validations).[28] In 2017, Saranburut *et al* prospectively validated the Framingham Heart Study risk score on a cohort using two different outcome definitions, i.e., they provided estimates for two model validations.[31] In 2017, Saranburut *et al* prospectively developed four models and validated two of them using cohort data, i.e., they provided estimates for six models (four derivations and two validations).[32] In 2019, Bradshaw *et al* used cross-sectional data to derive four models, one of them was validated on two populations (rural and urban), i.e. they provided estimates

For peer review only

For peer review only - http://bmjopen.bmj.com/site/about/guidelines.xhtml

for six models (four derivations and two validations).[27] In 2020, Asgari and colleagues prospectively validated a model from the Netherlands for 6- and 9-years CKD prediction, i.e. they provided estimates for two model validations.[26] In 2020, Wen *et al* prospectively derived two models.[29] Overall, fourteen models were derived and fifteen underwent validation (hence the 29 rows in Table 4).

Outcome ascertainment

Across all reports, CKD was defined as eGFR <60 mL/min/$1.73m^2$ [25-33] assessed by either the Modification of Diet Renal Disease (MDRD) formula[25 26 28 29 31 33] or the Chronic Kidney Disease Epidemiology Collaboration (CKD-EPI) formula.[27 30-32] In addition to the eGFR assessment, Bradshaw *et al*[27] and Wen *et al*[29] defined CKD as a urinary albumin-to-creatinine ratio (UACR) ≥30 mg/g. Mogueo *et al* validations also considered CKD as any nephropathy including stages I to V of the "Kidney Disease: Improving Global Outcomes (KDIGO)" classification.[25] Thakkinstian *et al*, also considered CKD as eGFR ≥60 mL/min/$1.73m^2$ if it had haematuria or UACR ≥30 mg/g [33] (Table 2).

Predictors and modelling

Logistic regression analysis was conducted in all derivation models.[27-30 32 33] Selection of the final predictors was based on modelling techniques: backward[27 28] and forward selection[29 30 32 33] (S3 Table). All studies categorized numerical variables. The most frequent predictors included in the models were: age, diabetes mellitus and sex (S2 Figure).

Model performance

All studies reported calibration and discrimination metrics, except for the validations by Bradshaw *et al*[27] and Carrillo-Larco *et al*[28] (S3 Table). Regarding discrimination metrics, the area under the Receiver Operating Characteristic (ROC) curve and C-statistic were over 63%[31] and 70%,[27] respectively. Among all studies, sensitivity ranged from 56.8%[29] to 84.0%,[25] specificity ranged from 65.1%[29] to 86.3%,[30] positive predictive value (PPV) ranged from 8.8%[28] to 33.8%,[29] and negative predictive value (NPV) ranged from 89.4%[29] to 99.1%.[28] The NPV was the best metric, consistently above 89.4% (Table 3).

For peer review only - http://bmjopen.bmj.com/site/about/guidelines.xhtml

Risk of bias

All studies showed a high risk of bias due to insufficient or inadequate analytical reporting. The flaw regarding the analysis criteria can be explained by how original reports handled missing data and predictors categorization. The participants and predictors criteria had low risk of bias in most of the reports. Most of the individual reports demonstrated an inappropriate evaluation of performance metrics.[26 28-33] Low applicability concern was noted (Table 4; S4Table).

DISCUSSION

Main findings

This systematic review summarized all available risk scores for CKD in LMIC. In so doing, we provided the most comprehensive list of CKD risk scores to enhance primary prevention and early diagnosis of CKD in LMIC. Although the available models had acceptable discrimination metrics and, when available, acceptable calibration metrics, these models had serious methodological limitations such as a reduced number of outcome events. The best performance metric across risk scores was the negative predictive value. Overall, CKD risk prediction tools in LMIC need rigorous development and validation so that they can be incorporated into clinical practice and interventions. The available evidence would not support using any of the available CKD risk scores across LMIC.

Limitations of the review

We did not search grey literature. We argue that this limitation would not substantially change our results because these sources are most likely not to have included a random sample of the general population and are likely to have included a small sample size with few outcome events. That is, we would not expect to find a report in the grey literature with a much better methodology than that of the studies herein summarised.

Limitations of the selected reports

Several LMIC do not have a CKD risk score, particularly countries in Central America and Oceania. This should encourage public health officers and researchers to develop CKD prediction models. They could conduct new epidemiological studies or leverage on available health surveys with kidney biomarkers. These models could have pragmatic and direct applications in clinical medicine, by providing a tool for early identification of CKD cases. Similarly, these models could inform public

For peer review only - http://bmjopen.bmj.com/site/about/guidelines.xhtml

health interventions and planning, by providing a tool to quantify the size of the population likely to have or to develop CKD.

Clinical guidelines state that CKD is defined as a sustained structural or functional kidney damage for ≥3 months.[34] In the studies herein summarised, CKD was defined at one point in time. Future work could expand the definition of CKD to also incorporate the lapse during which the patient had kidney damage. In addition, different procedures were used to define CKD including eGFR, proteinuria, and UACR. Even amongst those studies in which CKD was defined with eGFR, they used different equations to compute the eGFR. Researchers and practitioners in LMIC could agree on the best and most pragmatic as well as cost-effective definition of CKD, so that future models could use this definition. This would improve the comparability and extrapolability of the models.

All reports in which a new CKD risk score was developed selected the predictors through univariate analyses,[27-30,32,33] which is not be the best approach to choose predictors.[35-37] Ideally, predictors should be selected based on expert knowledge, or amongst those with the strongest association evidence with CKD. In a similar vein, predictors selection should be guided by the target population. For example, CKD prediction models for populations in LMIC should prioritize simple biomarkers or inexpensive clinical evaluations (e.g., blood pressure). In this way, the risk score is likely to be used in clinical practice in resource-limited settings. Another relevant methodological limitation was how the original reports handled missing data. To the extent possible, multiple imputation should be implemented to maximize available data and to avoid potential bias by studying only observations with complete information.

Calibration assesses the degree of agreement between actual outcomes and model prediction, whereas discrimination is the ability of the model to differentiate people with and without the outcome. Calibration metrics need to be consistently reported and should inform the direction of the miscalibration. Most of the studies used the Hosmer-Lemeshow X^2 test as the calibration metric. Unfortunately, this test does not inform on whether the model prediction is overestimating or underestimating the observed risk; calibration plots are a useful alternative. Therefore, it was not always possible to reach strong conclusions about the performance of the available models. Prognostic models should be updated before they can be applied in a new target population. This process is known as recalibration. Because we found a handful of prognostic models in some

For peer review only

countries, it is debatable whether these can be successfully used in other populations. Available prognostic models for CKD would need to be recalibrated and independently validated in new target populations.

Clinical and public health relevance

The Latin American Society of Nephrology and Hypertension (Sociedad Latinoamericana de Nefrología e Hipertensión - SLANH) recommends to annually screen for CKD with several markers: blood pressure, serum creatinine, proteinuria and urinalysis.[38] The South African Renal Society (SARS) guidelines also recommend CKD screening annually, yet they focus on high-risk populations: people with diabetes, hypertension, or HIV.[39] This recommendation is endorsed by the Asian Forum for Chronic Kidney Disease Initiatives (AFCKDI), extending it to individuals ≥65 years, people consuming nephrotoxic substances, and those with family history of CKD and past history of acute kidney injury.[40] Although it seems reasonable to screen people with risk factors such as hypertension and diabetes, this approach may miss a large proportion of the high-risk population because they could be unaware of their condition.[41 42] In this case, risk scores could be useful because they can be applied to large populations regardless of whether they are aware of their hypertension or diabetes status. Unfortunately, our work would not support nor encourage the inclusion of available risk scores for CKD in clinical guidelines in LMIC. Instead, our results urgently call to improve risk prediction research in LMIC. Therefore, CKD risk scores could be included into clinical practice to identify high-risk individuals and to inform the patient's management plan as is the case in other fields such as cardiovascular primary prevention.

Conclusions

This systematic review of diagnostic and prognostic models of CKD did not find conclusive evidence to recommend the use of a single CKD score across LMIC. Nonetheless, we identified relevant efforts in Iran, India, Peru, South Africa, China and Thailand; these models would require further external validation before they can be applied in other LMIC. We encourage researchers and practitioners to develop and validate CKD risk scores, which are cost-efficient tools to early identify CKD prevalent and incident cases so that they can receive timely treatment.

For peer review only

For peer review only - http://bmjopen.bmj.com/site/about/guidelines.xhtml

Contributors:

RMC-L, DJA-G and EJA conceived the idea. RMC-L, DJA-G and EJA conducted the search. DJA-G and EJA wrote the manuscript. All authors approved the submitted version.

Carrillo Larco, Rodrigo M. (proxy) (contact); Aparcana-Granda, Diego J.; Ascencio, Edson J.

Funding: RMC-L is supported by a Wellcome Trust International Training Fellowship (214185/Z/18/Z).

Competing interests: None declared.

Patient consent for publication: Not required.

Data availability statement: Data sharing not applicable as no data sets generated for this study. Given the nature of systematic reviews, the data set generated and analysed for the current study is already available. All studies analysed for the present review are referenced for readers.

For peer review only

For peer review only - http://bmjopen.bmj.com/site/about/guidelines.xhtml

REFERENCES

1. Bikbov B, Purcell CA, Levey AS, et al. Global, regional, and national burden of chronic kidney disease, 1990–2017: a systematic analysis for the Global Burden of Disease Study 2017. *The Lancet* 2020;395(10225):709-33. doi: 10.1016/s0140-6736(20)30045-3
2. Foreman KJ, Marquez N, Dolgert A, et al. Forecasting life expectancy, years of life lost, and all-cause and cause-specific mortality for 250 causes of death: reference and alternative scenarios for 2016–40 for 195 countries and territories. *The Lancet* 2018;392(10159):2052-90. doi: 10.1016/s0140-6736(18)31694-5 [published Online First: 2018/10/21]
3. Xie Y, Bowe B, Mokdad AH, et al. Analysis of the Global Burden of Disease study highlights the global, regional, and national trends of chronic kidney disease epidemiology from 1990 to 2016. *Kidney International* 2018;94(3):567-81. doi: 10.1016/j.kint.2018.04.011
4. Yarnoff BO, Hoerger TJ, Simpson SK, et al. The cost-effectiveness of using chronic kidney disease risk scores to screen for early-stage chronic kidney disease. *BMC Nephrology* 2017;18(1):85. doi: 10.1186/s12882-017-0497-6 [published Online First: 2017/03/16]
5. Samanta R, Misir R, Mitra M. A reduced set of features for chronic kidney disease prediction. *Journal of Pathology Informatics* 2017;8(1):24. doi: 10.4103/jpi.jpi_88_16 [published Online First: 2017/07/15]
6. Chang H-L, Wu C-C, Lee S-P, et al. A predictive model for progression of CKD. *Medicine* 2019;98(26):e16186. doi: 10.1097/md.0000000000016186 [published Online First: 2019/07/03]
7. Lerner B, Desrochers S, Tangri N. Risk Prediction Models in CKD. *Seminars in Nephrology* 2017;37(2):144-50. doi: 10.1016/j.semnephrol.2016.12.004 [published Online First: 2017/04/16]
8. Tangri N, Inker LA, Hiebert B, et al. A Dynamic Predictive Model for Progression of CKD. *American Journal of Kidney Diseases* 2017;69(4):514-20. doi: 10.1053/j.ajkd.2016.07.030 [published Online First: 2016/10/04]
9. Ramspek CL, de Jong Y, Dekker FW, et al. Towards the best kidney failure prediction tool: a systematic review and selection aid. *Nephrology Dialysis Transplantation* 2020;35(9):1527-38. doi: 10.1093/ndt/gfz018
10. Remuzzi G, Echouffo-Tcheugui JB, Kengne AP. Risk Models to Predict Chronic Kidney Disease and Its Progression: A Systematic Review. *PLoS Medicine* 2012;9(11):e1001344. doi: 10.1371/journal.pmed.1001344 [published Online First: 2012/11/28]
11. Page MJ, McKenzie JE, Bossuyt PM, et al. The PRISMA 2020 statement: An updated guideline for reporting systematic reviews. *PLOS Medicine* 2021;18(3):e1003583. doi: 10.1371/journal.pmed.1003583
12. Ascencio EJ, Aparcana-Granda DJ, Carrillo-Larco RM. Chronic kidney disease in Low- and Middle-Income Countries: Protocol for a systematic review of diagnostic and prognostic models. *medRxiv* 2021:2021.04.24.21256041. doi: 10.1101/2021.04.24.21256041
13. Debray TPA, Damen JAAG, Snell KIE, et al. A guide to systematic review and meta-analysis of prediction model performance. *BMJ (Clinical research ed)* 2017;356:i6460. doi: 10.1136/bmj.i6460 [published Online First: 2017/01/07]
14. Moons KGM, de Groot JAH, Bouwmeester W, et al. Critical Appraisal and Data Extraction for Systematic Reviews of Prediction Modelling Studies: The CHARMS Checklist. *PLoS Medicine* 2014;11(10):e1001744. doi: 10.1371/journal.pmed.1001744 [published Online First: 2014/10/15]
15. The World Bank. World Bank Country and Lending Groups [Available from: https://datahelpdesk.worldbank.org/knowledgebase/articles/906519-world-bank-country-and-lending-groupsfiles/235/906519-world-bank-country-and-lending-groups.html.
16. Christodoulou E, Ma J, Collins GS, et al. A systematic review shows no performance benefit of machine learning over logistic regression for clinical prediction models. *Journal of clinical epidemiology* 2019;110:12-22. doi: 10.1016/j.jclinepi.2019.02.004
17. Shillan D, Sterne JAC, Champneys A, et al. Use of machine learning to analyse routinely collected intensive care unit data: a systematic review. *Critical Care* 2019;23(1):284. doi: 10.1186/s13054-019-2564-9
18. Collins GS, Dhiman P, Andaur Navarro CL, et al. Protocol for development of a reporting guideline (TRIPOD-AI) and risk of bias tool (PROBAST-AI) for diagnostic and prognostic prediction model studies based on artificial intelligence. *BMJ Open* 2021;11(7):e048008. doi: 10.1136/bmjopen-2020-048008

For peer review only - http://bmjopen.bmj.com/site/about/guidelines.xhtml

19. Ouzzani M, Hammady H, Fedorowicz Z, et al. Rayyan—a web and mobile app for systematic reviews. *Systematic Reviews* 2016;5(1):210. doi: 10.1186/s13643-016-0384-4 [published Online First: 2016/12/07]
20. Moons KGM, Wolff RF, Riley RD, et al. PROBAST: A Tool to Assess Risk of Bias and Applicability of Prediction Model Studies: Explanation and Elaboration. *Annals of internal medicine* 2019;170(1):W1-W33. doi: 10.7326/m18-1377 [published Online First: 2019/01/01]
21. Wolff RF, Moons KGM, Riley RD, et al. PROBAST: A Tool to Assess the Risk of Bias and Applicability of Prediction Model Studies. *Annals of internal medicine* 2019;170(1):51-58. doi: 10.7326/m18-1376 [published Online First: 2019/01/01]
22. Zhou JH, Wei Y, Lyu YB, et al. Prediction of 6-year incidence risk of chronic kidney disease in the elderly aged 65 years and older in 8 longevity areas in China. *Zhonghua liu xing bing xue za zhi = Zhonghua liuxingbingxue zazhi* 2020;41(1):42-47. doi: 10.3760/cma.j.issn.0254-6450.2020.01.009 [published Online First: 2020/02/18]
23. Mahapatra H, Gupta Y, Sharma N, et al. Identification of high-risk population and prevalence of kidney damage among asymptomatic central government employees in Delhi, India. *Saudi Journal of Kidney Diseases and Transplantation* 2016;27(2):362-70. doi: 10.4103/1319-2442.178564
24. Alvarez-Gregori JA, Robles NR, Mena C, et al. The value of a formula including haematocrit, blood urea and gender (HUGE) as a screening test for chronic renal insufficiency. *The journal of nutrition, health & aging* 2011;15(6):480-84. doi: 10.1007/s12603-011-0001-0 [published Online First: 2011/05/31]
25. Mogueo A, Echouffo-Tcheugui JB, Matsha TE, et al. Validation of two prediction models of undiagnosed chronic kidney disease in mixed-ancestry South Africans. *BMC Nephrology* 2015;16(1):94. doi: 10.1186/s12882-015-0093-6 [published Online First: 2015/07/05]
26. Asgari S, Moosaie F, Khalili D, et al. External validation of the European risk assessment tool for chronic cardio-metabolic disorders in a Middle Eastern population. *Journal of Translational Medicine* 2020;18(1):267. doi: 10.1186/s12967-020-02434-5 [published Online First: 2020/07/04]
27. Bradshaw C, Kondal D, Montez-Rath ME, et al. Early detection of chronic kidney disease in low-income and middle-income countries: development and validation of a point-of-care screening strategy for India. *BMJ Global Health* 2019;4(5):e001644. doi: 10.1136/bmjgh-2019-001644 [published Online First: 2019/09/24]
28. Carrillo-Larco RM, Miranda JJ, Gilman RH, et al. Risk score for first-screening of prevalent undiagnosed chronic kidney disease in Peru: the CRONICAS-CKD risk score. *BMC Nephrology* 2017;18(1):343. doi: 10.1186/s12882-017-0758-4 [published Online First: 2017/12/01]
29. Wen J, Hao J, Zhang Y, et al. Risk scores for predicting incident chronic kidney disease among rural Chinese people: a village-based cohort study. 2020;21(1):120.
30. Wu L, Guo VY, Wong CKH, et al. Innovative non-invasive model for screening reduced estimated glomerular filtration rate in a working population. *Nephrology* 2017;22(11):892-98. doi: 10.1111/nep.12921 [published Online First: 2016/09/16]
31. Saranburut K, Vathesatogkit P, Chittamma A, et al. Evaluation of the Framingham Heart Study risk factors and risk score for incident chronic kidney disease at 10 years in a Thai general population. 2017;49(5):851-57.
32. Saranburut K, Vathesatogkit P, Thongmung N, et al. Risk scores to predict decreased glomerular filtration rate at 10 years in an Asian general population. 2017;18(1):240.
33. Thakkinstian A, Ingsathit A, Chaiprasert A, et al. A simplified clinical prediction score of chronic kidney disease: A cross-sectional-survey study. *BMC Nephrology* 2011;12(1):45. doi: 10.1186/1471-2369-12-45 [published Online First: 2011/09/29]
34. Kidney Disease: Improving Global Outcomes (KDIGO) CKD Work Group. KDIGO 2012 clinical practice guideline for the evaluation and management of chronic kidney disease. *Kidney Int Suppl* 2013;3:1-150.
35. Harrell FE. Regression Modeling Strategies, With Applications to Linear Models, Logistic Regression, and Survival Analysis. New York: Springer 2001.
36. Steyerberg EW. Clinical Prediction Models: A Practical Approach to Development, Validation, and Updating. New York: Springer 2009.
37. Sun G-W, Shook TL, Kay GL. Inappropriate use of bivariable analysis to screen risk factors for use in multivariable analysis. *Journal of clinical epidemiology* 1996;49(8):907-16. doi: 10.1016/0895-4356(96)00025-x [published Online First: 1996/08/01]

For peer review only - http://bmjopen.bmj.com/site/about/guidelines.xhtml

38. Sociedad Latinoamericana de Nefrología e H. Guías Latinoamericanas de Práctica Clínica sobre la Prevención, Diagnóstico y Tratamiento de los Estadios 1-5 de la Enfermedad Renal Crónica. *1* 2012
39. South African Renal Society. Guideline for the optimal care of patients on chronic dialysis in South Africa. 2015
40. Li PK-T. Asian chronic kidney disease best practice recommendations: Positional statements for early detection of chronic kidney disease from Asian Forum for Chronic Kidney Disease Initiatives (AFCKDI). *Nephrology* 2011;16:633-41.
41. Wareham NJ, Manne-Goehler J, Geldsetzer P, et al. Health system performance for people with diabetes in 28 low- and middle-income countries: A cross-sectional study of nationally representative surveys. *PLOS Medicine* 2019;16(3):e1002751. doi: 10.1371/journal.pmed.1002751
42. Zhou B, Carrillo-Larco RM, Danaei G, et al. Worldwide trends in hypertension prevalence and progress in treatment and control from 1990 to 2019: a pooled analysis of 1201 population-representative studies with 104 million participants. *The Lancet* 2021 doi: 10.1016/s0140-6736(21)01330-1 [published Online First: 2021/08/28]

For peer review only

For peer review only - http://bmjopen.bmj.com/site/about/guidelines.xhtml

428 **TABLES:**

429 **Table 1. CHARMS criteria to define research question and strategy.**

Concept	Criteria
Prognostic or diagnostic?	Both - this review focused on diagnostic and prognostic risk scores for chronic kidney disease (CKD)
Scope	Diagnostic/prognostic models to inform physicians, researchers and the general population whether they are likely to have CKD (i.e., diagnostic) or will be likely to have CKD (i.e., prognostic)
Type of prediction modelling studies	• Diagnostic/prognostic models with external validation<br>• Diagnostic/prognostic models without external validation<br>• Diagnostic/prognostic models validation
Target population to whom the prediction model applies	General adult population in Low- and Middle-Income Countries (LMIC). No age or gender restrictions
Outcome to be predicted	CKD (diagnostic or prognostic)
Time span of prediction	Any, prognostic models will not be included/excluded based on the prediction time span
Intended moment of using the model	Diagnostic/prognostic models to be used in asymptomatic adults of LMIC to ascertain current CKD status or future risk of developing CKD. These models could be used for screening, treatment allocation in primary prevention, or research purposes

430 Based on the CHARMS checklist.[14]

For peer review only

Table 2. General characteristics.

Nº of report	Study	Country	Outcome prevalence (%)	Mean age (years)	Men<br><br>(%)	Outcome details	Baseline sample size	Number of outcome events	Outcome events per candidate predictors
1	Asgari *et al*, 2020	Iran	**6-years validation:** 22.08<br><br>**9-years validation:** 41.94	**6-years validation:** 46.02<br><br>**9-years validation:** NI	**6-years validation:** 40.1<br><br>**9-years validation:** 40.6	CKD was defined as eGFR <60 mL/min/1.73 m^2, provided by the MDRD formula	**6-years validation:** 3,270<br><br>**9-years validation:** 3,240	**6-years validation:** 722<br><br>**9-years validation:** 1,359	**For every model validation:** n/a
2	Bradshaw *et al*, 2019	India	**For every model derivation:** 10.89<br><br>**For every model validation:** NI	**For every model derivation:** 44.9<br><br>**For every model validation:** NI	**For every model derivation:** 46.8<br><br>**For every model validation:** NI	CKD was defined as an eGFR rate <60 mL/min/1.73 m^2 (estimated with the CKD-EPI equation) or UACR ≥30 mg/g	**For every model derivation:** 8,698<br><br>**Urban model validation:** 4,065<br><br>**Rural model validation:** 4,940	**For every model derivation:** 947<br><br>**For every model validation:** NI	**Model 1 derivation:** 31.6<br><br>**Model 2 derivation:** 41.2<br><br>**Model 3a derivation:** 135.3<br><br>**Model 3b derivation:** 118.4<br><br>**For every model validation:** n/a
3	Carrillo-Larco *et al*, 2017	Peru	**For every model derivation:** 3.42<br><br>**For every model validation:** 5.41	**For every model derivation:** 57.7<br><br>**For every model validation:** 57.1	**For every model derivation:** 49.4<br><br>**For every model validation:** 47.7	CKD was defined as eGFR <60 mL/min/1.73 m^2, provided by the MDRD formula	**For every model derivation:** 2,368<br><br>**For every model validation:** 1,459	**For every model derivation:** 81<br><br>**For every model validation:** 79	**Complete model derivation:** 2.25<br><br>**Lab-free model derivation:** 3.1<br><br>**For every model validation:** n/a
4	Mogueo *et al*, 2015	South Africa	**For every eGFR model validation:** 28.71<br><br>**For every eGFR or proteinuria model validation:** 29.71	**For every model validation:** 55	**For every model validation:** 23.4	CKD was defined as eGFR <60 mL/min/1.73 m^2, provided by the 4-variable MDRD formula	**For every model validation:** 902	**For every eGFR model validation:** 259<br><br>**For every eGFR or proteinuria model validation:** 268	**For every model validation:** n/a

For peer review only

5	Saranburut *et al*, 2017 - Framingham Heart Study	Thailand	**MDRD model validation:** 10.37<br><br>**CKD-EPI model validation:** 10.01	**MDRD model validation:** 54.6<br><br>**CKD-EPI model validation:** 54.7	**MDRD model validation:** 70.8<br><br>**CKD-EPI model validation:** 71.5	**MDRD model validation:** CKD was defined as eGFR <60 mL/min/1.73 m^2, provided by the MDRD formula<br><br>**CKD-EPI model validation:** CKD was defined as eGFR <60 mL/min/1.73 m^2, provided by the CKD-EPI equation	**MDRD model validation:** 2,141<br><br>**CKD-EPI model validation:** 2,328	**MDRD model validation:** 222<br><br>**CKD-EPI model validation:** 233	**For every model validation:** n/a
6	Saranburut *et al*, 2017	Thailand	**For every model derivation:** 8.51<br><br>**For every model validation:** 1.94	**For every model derivation:** 51.3<br><br>**For every model validation:** 45.6	**For every model derivation:** 70.5<br><br>**For every model validation:** 70.5	CKD was defined as a preserved GFR (eGFR ≥60 mL/min/1.73m^2) at baseline and subsequently developed decreased GFR (eGFR <60 mL/min/1.73m^2) at the 10-year follow-up, provided by the Two-level Race Variable CKD-EPI equation (using the non-black coefficient)	**For every model derivation:** 3,186<br><br>**For every model validation:** 1,395	**For every model derivation:** 271<br><br>**For every model validation:** 27	**Model 1 derivation:** 18.1<br><br>**Model 1 BMI derivation:** 18.1<br><br>**Model 2 derivation:** 16.9<br><br>**Model 3 derivation:** 12.3<br><br>**For every validation model:** n/a
7	Thakkinstian *et al*, 2011	Thailand	18.10	45.2	45.5	CKD was defined as a combination of stages I to V. CKD stage I & II was defined as eGFR ≥90 and eGFR 60-89 ml/min/1.73 m^2, respectively; with haematuria or UACR ≥30 mg/g. CKD stage III, IV, and V was defined as eGFR 30-59, 15-29, and <15 ml/min/1.73 m^2, respectively; regardless of kidney damage (eGFR was calculated using the MDRD formula)	3,459	626	16.9
8	Wen *et al*, 2020	China	**For every derivation model:** 18.06	**For every derivation model:** 50	**For every derivation model:** 44.7	CKD was defined as an eGFR rate <60 mL/min/1.73 m^2 (assessed with the modified Chinese MDRD equation) or UACR ≥30 mg/g	**For every derivation model:** 3,266	**For every derivation model:** 590	**For every derivation model:** NI
9	Wu *et al*, 2016	China	**Model derivation:** 2.05<br><br>**Model validation:** 1.10	**Model derivation:** 45.3<br><br>**Model validation:** 41.8	**Model derivation:** 56.7<br><br>**Model validation:** 63.7	CKD was defined as eGFR <60 mL/min/1.73 m^2, provided by the CKD-EPI equation	**Model derivation:** 14,374<br><br>**Model validation:** 4,371	**Model derivation:** 294<br><br>**Model validation:** 48	**Model derivation:** NI<br><br>**Model validation:** n/a

CKD, chronic kidney disease; CKD-EPI, chronic kidney disease epidemiology collaboration; eGFR, estimated glomerular filtration rate; GFR, glomerular filtration rate; KDIGO, MDRD, modification of diet renal disease; n/a, not applicable; NI, no information; UACR, urinary albumin-to-creatinine ratio.

For peer review only - http://bmjopen.bmj.com/site/about/guidelines.xhtml

434 **Table 3. Performance metrics.**

Nº	Study	Discrimination (%)	Classification measures
1	Asgari *et al*, 2020	**6-years validation:** AUC (95% CI) for final intercept adjusted model = Male: 76 (72-79) and Female: 71 (69-73)<br><br>**9-years validation:** AUC (95% CI) for final intercept adjusted model = Male: 71 (67-74) and Female: 70 (68-73)	**6-years validation:** For men at a cut-off of 25: sensitivity=72.7%; specificity=67.6%. For women at a cut-off of 19: sensitivity=66.8%; specificity=65.6%<br><br>**9-years validation:** For men at a cut-off of 25: sensitivity=64.5%; specificity=69.5%. For women at a cut-off of 23: sensitivity=56.9%; specificity=76.6%
2	Bradshaw *et al*, 2019	**Model 1 derivation:** C-statistic (95% CI) = 79 (78-81)<br><br>**Model 2 derivation:** C-statistic (95% CI) = 73 (72-75)<br><br>**Model 3a derivation:** C-statistic (95% CI) = 77 (75-79)<br><br>**Model 3b derivation:** C-statistic (95% CI) = 77 (76-79)<br><br>**Urban validation:** C-statistic (95% CI) = 74 (73-74)<br><br>**Rural validation:** C-statistic (95% CI) = 70 (69-71)	**Model 1 derivation:** At a cut-off of 0.09: sensitivity=72%; specificity=72%; positive predictive value=24%; negative predictive value=96%<br><br>**Model 2 derivation:** At a cut-off of 0.09: sensitivity=68%; specificity=67%; positive predictive value=20%; negative predictive value=95%<br><br>**Model 3a derivation:** At a cut-off of 0.09: sensitivity=71%; specificity=70%; positive predictive value=22%; negative predictive value=95%<br><br>**Model 3b derivation:** At a cut-off of 0.09: sensitivity=71%; specificity=70%; positive predictive value=22%; negative predictive value=95%<br><br>**Urban model validation:** NI<br><br>**Rural model validation:** NI
3	Carrillo-Larco *et al*, 2017	**Complete model derivation:** AUC = 76.2<br><br>**Lab-free model derivation:** AUC = 76<br><br>**Complete model validation:** AUC = 70<br><br>**Lab-free model validation:** AUC = 70	**Complete model derivation:** At a cut-off of 2: sensitivity=82.5%; specificity=70.0%; positive predictive value=8.8%; negative predictive value=99.1%; likelihood ratio positive=2.8; likelihood ratio negative=0.3<br><br>**Lab-free model derivation:** At a cut-off of 2: sensitivity=80%; specificity=72%; positive predictive value=9.1%; negative predictive value=99%; likelihood ratio positive=2.9; likelihood ratio negative=0.3<br><br>**Complete model validation:** At a cut-off of 2: sensitivity=70.5%; specificity=69.1%; positive predictive value=11.4%; negative predictive value=97.6%; likelihood ratio positive=2.3; likelihood ratio negative=0.4<br><br>**Lab-free model validation:** At a cut-off of 2: sensitivity=70.5%; specificity=69.7%; positive predictive value=11.6%; negative predictive value=97.7%; likelihood ratio positive=2.3; likelihood ratio negative=0.4

For peer review only

For peer review only - http://bmjopen.bmj.com/site/about/guidelines.xhtml

4	Mogueo *et al*, 2015	**South Korean eGFR model validation:** C-statistic (95% CI) = 79.7 (76.5-82.9)<br><br>**Thai eGFR model validation:** C-statistic (95% CI) = 76 (72.6-79.3)<br><br>**South Korean eGFR or proteinuria model validation:** C-statistic (95% CI) = 81.1 (78.0-84.2)<br><br>**Thai eGFR or proteinuria model validation:** C-statistic (95% CI) = 77.2 (73.9-80.5)	**South Korean eGFR model validation:** At a cut-off of 0.30: sensitivity=82%; specificity=67%<br><br>**Thai eGFR model validation:** At a cut-off of 0.31: sensitivity=73%; specificity=72%<br><br>**South Korean eGFR or proteinuria model validation:** At a cut-off of 0.31: sensitivity=84%; specificity=68%<br><br>**Thai eGFR or proteinuria model validation:** At a cut-off of 0.32: sensitivity=74%; specificity=73%
5	Saranburut *et al*, 2017 - Framingham Heart Study	**MDRD model validation:** AUC (95% CI) = 69 (66-73)<br><br>**CKD-EPI model validation:** AUC (95% CI) = 63 (57-65)	**MDRD model validation:** NI<br><br>**CKD-EPI model validation:** NI
6	Saranburut *et al*, 2017 - Model 1 (derivation Clinical only)	**Model 1 derivation:** AUC (95% CI) = 72 (69-75)<br><br>**Model 1 BMI derivation:** AUC (95% CI) = 72 (69-75)<br><br>**Model 2 derivation:** AUC (95% CI) = 79 (76-82)<br><br>**Model 3 derivation:** AUC (95% CI) = 80 (77-82)<br><br>**Model 1 validation:** AUC (95% CI) = 66 (55-78)<br><br>**Model 2 validation:** AUC (95% CI) = 88 (80-95)	**Model 1 derivation:** NI<br><br>**Model 1 BMI derivation:** NI<br><br>**Model 2 derivation:** NI<br><br>**Model 3 derivation:** NI<br><br>**Model 1 validation:** NI<br><br>**Model 2 validation:** NI
7	Thakkinstian *et al*, 2011 (derivation)	C-statistic of internal validation = 74.1	At a cut-off of 5: sensitivity=76%; specificity=69%
8	Wen *et al*, 2020 - Simple Risk Score (derivation)	**Simple model derivation:** AUC (95% CI) = 71.7 (68.9-74.4)<br><br>**Best-fit model derivation:** AUC (95% CI) = 72.1 (69.3-74.8)	**Simple model derivation:** At a cut-off of 14: sensitivity=70.5%; specificity=65.1%; positive predictive value=29.8%; negative predictive value=91.3%; likelihood ratio positive=2.0; likelihood ratio negative=0.5<br><br>**Best-fit model derivation:** At a cut-off of 24: sensitivity=56.8%; specificity=76.6%; positive predictive value=33.8%; negative predictive value=89.4%; likelihood ratio positive=2.4 likelihood ratio negative=0.6
9	Wu *et al*, 2016 (derivation)	**Model derivation:** AUC (95% CI) = 89.4 (86.1-92.6)<br><br>**Model validation:** AUC (95% CI) = 88.0 (82.9-93.1)	**Model derivation:** At a cut-off of 36: sensitivity=82%; specificity=86.3%<br><br>**Model validation:** NI

AUC, area under the curve; CI, confident interval; NI, no information.

For peer review only - http://bmjopen.bmj.com/site/about/guidelines.xhtml

For peer review only

436 **Table 4: Risk of bias assessment of individual diagnostic/prediction models**

Study	Objective	Risk of Bias (RoB)				Applicability			Overall	
		Participants	Predictors	Outcome	Analysis	Participants	Predictors	Outcome	RoB	Applicability
Asgari *et al*, 2020 European Risk Assessment tool (6-years)	Validation	+	+	?	-	+	+	+	-	+
Asgari *et al*, 2020 European Risk Assessment tool (9-years)	Validation	+	+	?	-	+	+	+	-	+
Bradshaw *et al*, 2019 - Model 1	Derivation	+	+	?	-	+	+	+	-	+
Bradshaw *et al*, 2019 - Model 2	Derivation	+	+	?	-	+	+	+	-	+
Bradshaw *et al*, 2019 - Model 3a	Derivation	+	+	?	-	+	+	+	-	+
Bradshaw *et al*, 2019 - Model 3b	Derivation	+	+	?	-	+	+	+	-	+
Bradshaw *et al*, 2019 - Model 3a (CARRS-I urban)	Validation	+	+	?	-	+	+	+	-	+
Bradshaw *et al*, 2019 - Model 3a (UDAY rural)	Validation	+	+	?	-	+	+	+	-	+
Carrillo-Larco *et al*, 2017 - CRONICAS-CKD (complete)	Derivation	+	+	+	-	+	+	+	-	+
Carrillo-Larco *et al*, 2017 - CRONICAS-CKD (lab-free)	Derivation	+	+	+	-	+	+	+	-	+
Carrillo-Larco *et al*, 2017 - CRONICAS-CKD (complete)	Validation	+	+	+	-	+	+	+	-	+
Carrillo-Larco *et al*, 2017 - CRONICAS-CKD (lab-free)	Validation	+	+	+	-	+	+	+	-	+
Mogueo *et al*, 2015 – South Korean model (eGFR)	Validation	+	+	?	-	+	+	+	-	+
Mogueo *et al*, 2015 - Thai model (eGFR)	Validation	+	+	?	-	+	+	+	-	+
Mogueo *et al*, 2015 – South Korean model (eGFR or	Validation	+	+	?	-	+	+	+	-	+

For peer review only - http://bmjopen.bmj.com/site/about/guidelines.xhtml

For peer review only

proteinuria)										
Mogueo *et al*, 2015 - Thai model (eGFR or proteinuria)	Validation	+	+	?	-	+	+	+	-	+
Saranburut *et al*, 2017 - Framingham Heart Study (MDRD)	Validation	+	+	?	-	+	+	+	-	+
Saranburut *et al*, 2017 - Framingham Heart Study (CKD-EPI)	Validation	+	+	?	-	+	+	+	-	+
Saranburut *et al*, 2017 - Model 1 (Clinical only)	Derivation	+	+	?	-	+	+	+	-	+
Saranburut *et al*, 2017 - Model 1 BMI (Clinical only)	Derivation	+	+	?	-	+	+	+	-	+
Saranburut *et al*, 2017 - Model 2 (Clinical + Limited laboratory tests)	Derivation	+	+	?	-	+	+	+	-	+
Saranburut *et al*, 2017 - Model 3 (Clinical + Full laboratory tests)	Derivation	+	+	?	-	+	+	+	-	+
Saranburut *et al*, 2017 - Model 1 (Clinical only)	Validation	+	+	?	-	+	+	+	-	+
Saranburut *et al*, 2017 - Model 2 (Clinical + Limited laboratory tests)	Validation	+	+	?	-	+	+	+	-	+
Thakkinstian *et al*, 2011	Derivation	+	+	?	-	+	+	+	-	+
Wen *et al*, 2020 - Simple Risk Score	Derivation	+	+	?	-	+	+	+	-	+
Wen *et al*, 2020 - Best-fit Risk Score	Derivation	+	+	?	-	+	+	+	-	+
Wu *et al*, 2016	Derivation	+	+	?	-	+	+	+	-	+
Wu *et al*, 2016	Validation	+	+	?	-	+	+	+	-	+

PROBAST = Prediction model Risk of Bias ASsessment Tool;[20 21] RoB = risk of bias. + indicates low RoB/low concern regarding applicability; − indicates high RoB/high concern regarding applicability; and ? indicates unclear RoB/unclear concern regarding applicability.

For peer review only

For peer review only - http://bmjopen.bmj.com/site/about/guidelines.xhtml

FIGURES

Figure 1. PRISMA 2020 flow diagram.

SUPPLEMENTARY MATERIAL

S1 Text. Protocol.

S1 Table. PRISMA 2020 checklist.

S2 Table. Search terms.

S3 Table. Data extraction form.

S4 Table. Risk of bias and applicability

S1 Figure. Countries where studies were conducted. LMIC that developed and/or validated models included in this review (Green). Moreover, Asgari *et al*,[26] Mogueo *et al*[25] and Saranburut *et al*[31] validated risk models that were originally derivated in the Netherlands, South Korea and the United States, respectively (Blue).

S2 Figure. Predictors included in the final models. The colours of the bars identify the underlying characteristic of predictors inherent to: the subject (purple), anthropometrics (blue), clinical assessment and history (green), and laboratory measures (yellow).

For peer review only

For peer review only - http://bmjopen.bmj.com/site/about/guidelines.xhtml

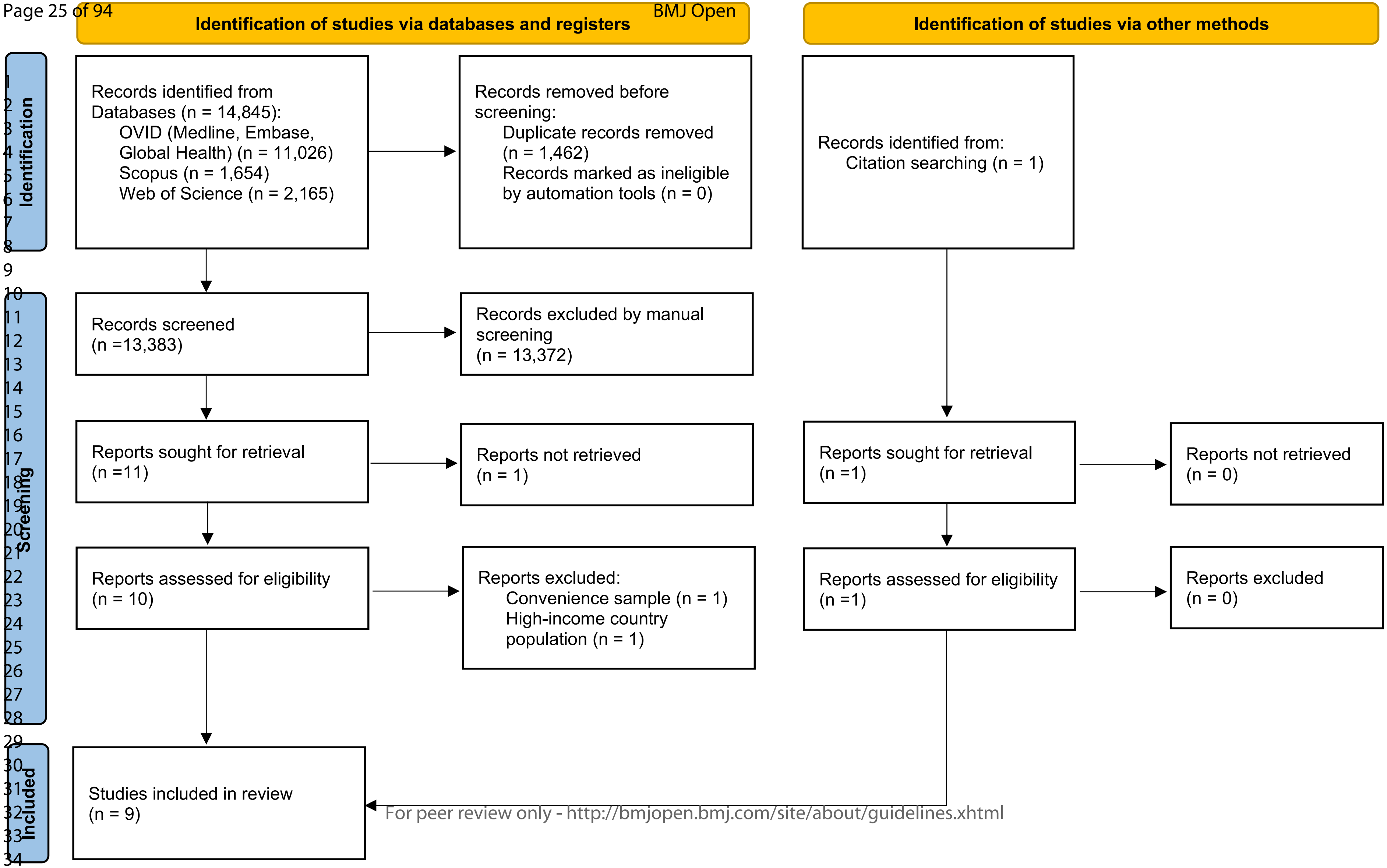
Identification of studies via databases and registers
Identification of studies via other methods
Identification
Screening
Included
Records identified from
Databases (n = 14,845):
OVID (Medline, Embase, Global Health) (n = 11,026)
Scopus (n = 1,654)
Web of Science (n = 2,165)
Records removed before screening:
Duplicate records removed (n = 1,462)
Records marked as ineligible by automation tools (n = 0)
Records identified from:
Citation searching (n = 1)
Records screened
(n =13,383)
Records excluded by manual screening
(n = 13,372)
Reports sought for retrieval
(n =11)
Reports not retrieved
(n = 1)
Reports sought for retrieval
(n =1)
Reports not retrieved
(n = 0)
Reports assessed for eligibility
(n = 10)
Reports excluded:
Convenience sample (n = 1)
High-income country population (n = 1)
Reports assessed for eligibility
(n =1)
Reports excluded
(n = 0)
Studies included in review
(n = 9)

Supplementary Material

. A systematic review of diagnostic and prognostic models of Chronic kidney disease in Low- and Middle- Income Countries

For peer review only

For peer review only - http://bmjopen.bmj.com/site/about/guidelines.xhtml

S1 Text: Protocol (also available at https://doi.org/10.1101/2021.04.24.21256041) 1
S1 Table: PRISMA Checklist 6
S2 Table: Search terms 11
S2.1 Table: Embase, Medline and Global Health (OVID) 11
S2.2 Table: SCOPUS 12
S2.3 Table: WEB OF SCIENCE 14
S3 Table: Data extraction form (by chapters) 15
S3.1 Table: Source of data and participants 15
S3.2 Table:: Outcome 26
S3.3 Table: Candidate predictors 31
S3.4 Table: Sample size and missing data 44
S3.5 Table: Model development 46
S3.6 Table: Model performance 49
S3.7 Table: Results 53
S3.8 Table: Discussion 55
S4 Table: PROBAST 57
S4.1 Table: Risk of Bias (RoB) 57
S4.2 Table: Applicability 65
S1 Figure: Countries where studies were conducted. 67
S2 Figure: Predictors included in the final models. 68

For peer review only

For peer review only - http://bmjopen.bmj.com/site/about/guidelines.xhtml

S1 Text: Protocol (also available at https://doi.org/10.1101/2021.04.24.21256041)

Chronic Kidney Disease in Low- and Middle- Income Countries: Protocol for a systematic review of diagnostic and prognostic models

Edson J Ascencio[1,2]

Diego J Aparcana-Granda[1,3]

Rodrigo M Carrillo-Larco[3,4]

1. Facultad de Medicina Alberto Hurtado, Universidad Peruana Cayetano Heredia, Lima, Perú.
2. Emerge, Emerging Diseases and Climate Change Research Unit, School of Public Health and Administration, Universidad Peruana Cayetano Heredia, Lima, Peru
3. CRONICAS Centre of Excellence in Chronic Diseases, Universidad Peruana Cayetano Heredia, Lima, Peru
4. Department of Epidemiology and Biostatistics, School of Public Health, Imperial College London, London, UK

Corresponding author

Rodrigo M Carrillo-Larco, MD

Department of Epidemiology and Biostatistics

School of Public Health, Imperial College London, London W2 1PG, UK

E-mail: rcarrill@ic.ac.uk

Phone: +44 0 7578240395

Competing interests: The authors declare no conflict of interests.

Support: RMC-L is supported by a Wellcome Trust International Training Fellowship (214185/Z/18/Z).

ABSTRACT

Background: Chronic Kidney Disease (CKD) is a highly prevalent condition with a large disease burden globally. In low- and middle-income countries (LMIC) the CKD screening challenges the health system. This systematic and comprehensive search of all CKD diagnostic and prognostic models in LMIC will inform screening strategies in LMIC following a risk-based approach.

Objective: To summarize all multivariate diagnostic and prognostic models for CKD in adults in LMIC.

Methods: Systematic review. Without date or language restrictions we will search Embase, Medline, Global Health (these three through Ovid), SCOPUS and Web of Science. We seek multivariable diagnostic or prognostic models which included a random sample of the general population. We will screen titles and abstracts; we will then study the selected reports. Both phases will be done by two reviewers independently. Data extraction will be performed by two researchers independently using a pre-specified Excel form (CHARMS model). We will evaluate the risk of bias with the PROBAST tool.

Conclusion: This systematic review will provide the most comprehensive list and critical appraisal of diagnostic and prognostic models for CKD available for the general population in LMIC. This evidence could

For peer review only

inform policies and interventions to improve CKD screening in LMIC following a risk-based approach, maximizing limited resources and reaching populations with limited access to CKD screening tests. This systematic review will also reveal methodological limitations and research needs to improve CKD diagnostic and prognostic models in LMIC.

Keywords: Chronic Kidney Disease; Diagnostic Models; Prognostic Models; Low- and Middle-income countries.

INTRODUCTION

Chronic kidney disease (CKD) is a highly prevalent condition that contributes to a large part of disease burden globally. Between 1990 and 2017, the health metrics of CKD showed a bleak profile: mortality rate, incidence and kidney transplantation rate increased by 2.8%, 29.3% and 34.4%, respectively.[1] CKD led to 1.2 million deaths in 2017 and in the best-case scenario, mortality is projected to increase to 2.2 million deaths[2] and become the 5th cause of years of life lost (YLL) by 2040.[3] Currently, 2.5 million of patients receive kidney transplantation therapy and it is projected to increase to 5.4 million by 2030.[1] CKD also reveals disparities between low- and middle-income countries (LMIC) and high income countries (HIC); for example, the age-standardised disability-adjusted life-year (DALY) rate due to CKD was the highest in LMIC between 1990-2017.[4] In LMIC, that remain as resource-constrained settings, there is a need for optimization of the CKD screening strategies which usually challenge the health system.[5]

Risk equations or risk scores are a cost-effective alternative for CKD screening.[6] These equations are less invasive and accepted by the general population;[7] also, they require less resources like laboratory tests.[8] Many scores were developed in high-income countries,[9-11] and they may not be used in LMIC because their accuracy is better where they have been developed.[12] Current strategies for CKD screening suggest studying people with risk factors (e.g. diabetes, hypertension).[13-15] These recommendations rely on studies where albuminuria and proteinuria were used as screening tools for identifying CKD patients.[16] Nevertheless, a systematic review found that using risk scores allows screening of a larger population and therefore can be useful for detecting more CKD cases.[6]

To date, there are no systematic reviews of diagnostic or prognostic models for CKD with a focus on LMIC.[17, 18] This limits our knowledge of what tools we have to enhance CKD screening in LMIC; similarly, this dearth of evidence prevents from planning future research to overcome the limitations of available models. This will be the first systematic review to fill these knowledge gaps in LMIC to improve and complement the CKD screening programmes in LMIC.

METHODS

Objective

To synthesise CKD diagnostic and prognostic models for the adult population of LMIC.

Study design

This systematic review and meta-analysis will be conducted following the preferred reporting items for systematic reviews and meta-analyses (PRISMA) 2020 guidelines.[19] We will also adhere to the recommendations for systematic reviews of diagnostic and prognostic models following the CHARMS guidelines[20] and the PROBAST tool to assess risk of bias.[21]

Eligibility criteria

Participants/population: We will include the general adult population (18 years and above) of LMIC with no gender restrictions. Studies following a population-based random sampling approach will be included. We will only include populations from LMIC according to The World Bank.[22] Conversely, studies with a study population of only patients (e.g., people with hypertension) or high-risk individuals (e.g., smokers) will be excluded. We will exclude studies with LMIC populations outside a LMIC.

Intervention, exposure: None (this review is looking at CKD diagnostic and prognostic models in LMIC).

For peer review only - http://bmjopen.bmj.com/site/about/guidelines.xhtml

Comparator, control: None (this review is looking at CKD diagnostic and prognostic models in LMIC).

Outcome: Diagnostic and prognostic models for CKD. The CKD diagnosis should have been based on a laboratory or imaging test including: urine albumin- creatinine ratio, urine protein-creatinine ratio, albumin excretion ratio, urine sediment, kidney images, kidney biopsy or the estimated glomerular filtration rate (eGFR). In other words, research in which CKD diagnosis was based on self-reported information only will not be considered. However, if a study combined both self-reported information and a laboratory or imaging tests, this will be included.

Types of studies: Studies with an observational design will be included, which encompasses crosssectional (for diagnostic models) and prospective longitudinal studies (for prognostic models). If we retrieve any systematic review on this subject, we will revise its reference list to identify relevant original sources.

Literature Search and Data collation

The search will be conducted in five search engines: Embase, Medline, Global Health (these three through Ovid), SCOPUS and Web of Science. No date or language restrictions will be set. The complete search strategy can be found in Supplementary Material.

Titles and abstracts will be screened by two researchers independently (DJA-G and EJA), looking for studies that meet the selection criteria above detailed. Full-text reports of the selected publications will be studied by two researchers independently (DJA-G and EJA). Discrepancies at any stage will be solved by consensus or by a third party (RMC-L).

During the full-text phase, if there are any original reports in which the population, methodology or results are not clear enough to assess the inclusion/exclusion criteria, we will contact the corresponding author by email. We will wait for two weeks, if we receive no answer and cannot solve our doubts through other means, this report will be excluded based on the lack of clarity to assess inclusion/exclusion criteria.

We will record the reasons for exclusion in the full-text phase and summarize the number of included/excluded reports following the PRISMA flow diagram.

Data extraction

We will develop a data extraction form following the CHARMS recommendations.[20] Data extraction will be conducted by two researchers independently; discrepancies will be solved by consensus or by a third party (RMC-L).

Risk of bias of individual studies

The risk of bias assessment of individual reports will be conducted using the Prediction model Risk Of

Bias ASsessment Tool (PROBAST) tool.[21]

Statistical Analysis

A qualitative synthesis is planned, whereby we will narratively synthesise the findings from the selected studies. We will summarize the key elements from each report such as study design, study population and characteristics of the study population. Also, we will summarize the key features of the risk scores as provided by each report, including discrimination, calibration, sensitivity, specificity, and predictive values. A quantitative synthesis will be carried out if the included studies are found to be sufficiently homogenous and we have at least four original reports.

Ethics

This review did not directly include human subjects. We considered this work as ‘low risk’ and did not request approval by an Ethics Committee. Results and opinions included in this protocol, and those included in the final report, are the author’s alone and do not represent those of the institutions to which they belong.

For peer review only - http://bmjopen.bmj.com/site/about/guidelines.xhtml

CONCLUSIONS

This systematic review will provide a comprehensive list of diagnostic and prognostic models for CKD for people in LMIC, along with their accuracy metrics. Currently, information lacks in LMIC where diagnostic and prognostic models could inform CKD screening strategies. Similarly, this work will elucidate the limitations of available diagnostic and prognostic models for CKD in LMIC, so that future research can be planned accordingly to overcome these caveats and deliver robust models to advance

CKD screening strategies in LMIC.

REFERENCES

1. Collaboration GBDCKD. Global, regional, and national burden of chronic kidney disease, 19902017: a systematic analysis for the Global Burden of Disease Study 2017. Lancet. 2020;395(10225):709-33.
2. Agudelo-Botero M, Valdez-Ortiz R, Giraldo-Rodriguez L, Gonzalez-Robledo MC, Mino-Leon D, Rosales-Herrera MF, et al. Overview of the burden of chronic kidney disease in Mexico: secondary data analysis based on the Global Burden of Disease Study 2017. BMJ Open. 2020;10(3):e035285.
3. Foreman KJ, Marquez N, Dolgert A, Fukutaki K, Fullman N, McGaughey M, et al. Forecasting life expectancy, years of life lost, and all-cause and cause-specific mortality for 250 causes of death: reference and alternative scenarios for 2016-40 for 195 countries and territories. Lancet. 2018;392(10159):2052-90.
4. Bikbov B, Purcell CA, Levey AS, Smith M, Abdoli A, Abebe M, et al. Global, regional, and national burden of chronic kidney disease, 1990–2017: a systematic analysis for the Global Burden of Disease Study 2017. The Lancet. 2020;395(10225):709-33.
5. Jafar TH, Ramakrishnan C, John O, Tewari A, Cobb B, Legido-Quigley H, et al. Access to CKD Care in Rural Communities of India: a qualitative study exploring the barriers and potential facilitators. BMC Nephrol. 2020;21(1):26.
6. Yarnoff BO, Hoerger TJ, Simpson SK, Leib A, Burrows NR, Shrestha SS, et al. The costeffectiveness of using chronic kidney disease risk scores to screen for early-stage chronic kidney disease. BMC Nephrol. 2017;18(1):85.
7. Bradshaw C, Kondal D, Montez-Rath ME, Han J, Zheng Y, Shivashankar R, et al. Early detection of chronic kidney disease in low-income and middle-income countries: development and validation of a point-of-care screening strategy for India. BMJ Glob Health. 2019;4(5):e001644.
8. Misir R, Mitra M, Samanta RK. A Reduced Set of Features for Chronic Kidney Disease Prediction. J Pathol Inform. 2017;8:24.
9. Chang HL, Wu CC, Lee SP, Chen YK, Su W, Su SL. A predictive model for progression of CKD.
10. Medicine (Baltimore). 2019;98(26):e16186.
11. Lerner B, Desrochers S, Tangri N. Risk Prediction Models in CKD. Semin Nephrol. 2017;37(2):144-50.
12. Tangri N, Inker LA, Hiebert B, Wong J, Naimark D, Kent D, et al. A Dynamic Predictive Model for Progression of CKD. American journal of kidney diseases : the official journal of the National Kidney Foundation. 2017;69(4):514-20.
13. Grant SW, Collins GS, Nashef SAM. Statistical Primer: developing and validating a risk prediction model. Eur J Cardiothorac Surg. 2018;54(2):203-8.
14. Boulware LE, Jaar BG, Tarver-Carr ME, Brancati FL, Powe NR. Screening for Proteinuria in US
15. AdultsA Cost-effectiveness Analysis. JAMA. 2003;290(23):3101-14.
16. Hoerger TJ, Wittenborn JS, Segel JE, Burrows NR, Imai K, Eggers P, et al. A health policy model of CKD: 2. The cost-effectiveness of microalbuminuria screening. American journal of kidney diseases : the official journal of the National Kidney Foundation. 2010;55(3):463-73.
17. Manns B, Hemmelgarn B, Tonelli M, Au F, Chiasson TC, Dong J, et al. Population based screening for chronic kidney disease: cost effectiveness study. BMJ. 2010;341:c5869.
18. Komenda P, Ferguson TW, Macdonald K, Rigatto C, Koolage C, Sood MM, et al. Costeffectiveness of primary screening for CKD: a systematic review. American journal of kidney diseases : the official journal of the National Kidney Foundation. 2014;63(5):789-97.
19. Echouffo-Tcheugui JB, Kengne AP. Risk models to predict chronic kidney disease and its progression: a systematic review. PLoS medicine. 2012;9(11):e1001344.
20. Ramspek CL, de Jong Y, Dekker FW, van Diepen M. Towards the best kidney failure prediction tool: a systematic review and selection aid. Nephrology Dialysis Transplantation. 2019;35(9):1527-38.

For peer review only - http://bmjopen.bmj.com/site/about/guidelines.xhtml

21. Page MJ, McKenzie JE, Bossuyt PM, Boutron I, Hoffmann TC, Mulrow CD, et al. The PRISMA 2020 statement: an updated guideline for reporting systematic reviews. BMJ. 2021;372:n71.
22. Moons KG, de Groot JA, Bouwmeester W, Vergouwe Y, Mallett S, Altman DG, et al. Critical appraisal and data extraction for systematic reviews of prediction modelling studies: the CHARMS checklist. PLoS medicine. 2014;11(10):e1001744.
23. Moons KGM, Wolff RF, Riley RD, Whiting PF, Westwood M, Collins GS, et al. PROBAST: A Tool to Assess Risk of Bias and Applicability of Prediction Model Studies: Explanation and Elaboration. Annals of internal medicine. 2019;170(1):W1-W33.
24. Bank TW. World Bank Country and Lending Groups 2021 [Available from: https://datahelpdesk.worldbank.org/knowledgebase/articles/906519-world-bank-countryand-lending-groups.

For peer review only

For peer review only - http://bmjopen.bmj.com/site/about/guidelines.xhtml

S1 Table: PRISMA Checklist

Section and Topic	Item #	Checklist item	Location where item is reported
**TITLE**			
Title	1	Identify the report as a systematic review.	page 01
**ABSTRACT**			
Abstract	2	See the PRISMA 2020 for Abstracts checklist.	page 02
**INTRODUCTION**			
Rationale	3	Describe the rationale for the review in the context of existing knowledge.	page 03
Objectives	4	Provide an explicit statement of the objective(s) or question(s) the review addresses.	page 04
**METHODS**			
Eligibility criteria	5	Specify the inclusion and exclusion criteria for the review and how studies were grouped for the syntheses.	page 04-05
Information sources	6	Specify all databases, registers, websites, organisations, reference lists and other sources searched or consulted to identify studies. Specify the date when each source was last searched or consulted.	page 04

For peer review only - http://bmjopen.bmj.com/site/about/guidelines.xhtml

Search strategy	7	Present the full search strategies for all databases, registers and websites, including any filters and limits used.	supplementary page 03-07
Selection process	8	Specify the methods used to decide whether a study met the inclusion criteria of the review, including how many reviewers screened each record and each report retrieved, whether they worked independently, and if applicable, details of automation tools used in the process.	page 05
Data collection process	9	Specify the methods used to collect data from reports, including how many reviewers collected data from each report, whether they worked independently, any processes for obtaining or confirming data from study investigators, and if applicable, details of automation tools used in the process.	page 05-06
Data items	10a	List and define all outcomes for which data were sought. Specify whether all results that were compatible with each outcome domain in each study were sought (e.g. for all measures, time points, analyses), and if not, the methods used to decide which results to collect.	page 04-05, table 1
	10b	List and define all other variables for which data were sought (e.g. participant and intervention characteristics, funding sources). Describe any assumptions made about any missing or unclear information.	page 04-05, table 1
Study risk of bias assessment	11	Specify the methods used to assess risk of bias in the included studies, including details of the tool(s) used, how many reviewers assessed each study and whether they worked independently, and if applicable, details of automation tools used in the process.	page 06
Effect measures	12	Specify for each outcome the effect measure(s) (e.g. risk ratio, mean difference) used in the synthesis or presentation of results.	NA
Synthesis methods	13a	Describe the processes used to decide which studies were eligible for each synthesis (e.g. tabulating the study intervention characteristics and comparing against the planned groups for each synthesis (item #5)).	page 06
	13b	Describe any methods required to prepare the data for presentation or synthesis, such as handling of missing summary statistics, or data conversions.	NA
	13c	Describe any methods used to tabulate or visually display results of individual studies and syntheses.	page 06

	13d	Describe any methods used to synthesize results and provide a rationale for the choice(s). If meta-analysis was performed, describe the model(s), method(s) to identify the presence and extent of statistical heterogeneity, and software package(s) used.	page 06
	13e	Describe any methods used to explore possible causes of heterogeneity among study results (e.g. subgroup analysis, meta-regression).	NA
	13f	Describe any sensitivity analyses conducted to assess robustness of the synthesized results.	NA
Reporting bias assessment	14	Describe any methods used to assess risk of bias due to missing results in a synthesis (arising from reporting biases).	NA
Certainty assessment	15	Describe any methods used to assess certainty (or confidence) in the body of evidence for an outcome.	page 11
**RESULTS**			
Study selection	16a	Describe the results of the search and selection process, from the number of records identified in the search to the number of studies included in the review, ideally using a flow diagram.	page 06-07
	16b	Cite studies that might appear to meet the inclusion criteria, but which were excluded, and explain why they were excluded.	page 06-07
Study characteristics	17	Cite each included study and present its characteristics.	page 08-09
Risk of bias in studies	18	Present assessments of risk of bias for each included study.	page 11, supplementary page 39-45

For peer review only - http://bmjopen.bmj.com/site/about/guidelines.xhtml

Results of individual studies	19	For all outcomes, present, for each study: (a) summary statistics for each group (where appropriate) and (b) an effect estimate and its precision (e.g. confidence/credible interval), ideally using structured tables or plots.	page 9-11
Results of syntheses	20a	For each synthesis, briefly summarise the characteristics and risk of bias among contributing studies.	page 9-11
	20b	Present results of all statistical syntheses conducted. If meta-analysis was done, present for each the summary estimate and its precision (e.g. confidence/credible interval) and measures of statistical heterogeneity. If comparing groups, describe the direction of the effect.	table 3
	20c	Present results of all investigations of possible causes of heterogeneity among study results.	NA
	20d	Present results of all sensitivity analyses conducted to assess the robustness of the synthesized results.	NA
Reporting biases	21	Present assessments of risk of bias due to missing results (arising from reporting biases) for each synthesis assessed.	NA
Certainty of evidence	22	Present assessments of certainty (or confidence) in the body of evidence for each outcome assessed.	page 11
**DISCUSSION**			
Discussion	23a	Provide a general interpretation of the results in the context of other evidence.	page 11
	23b	Discuss any limitations of the evidence included in the review.	page 11-13
	23c	Discuss any limitations of the review processes used.	page 11-13

For peer review only - http://bmjopen.bmj.com/site/about/guidelines.xhtml

	23d	Discuss implications of the results for practice, policy, and future research.	page 14-15
**OTHER INFORMATION**			
Registration and protocol	24a	Provide registration information for the review, including register name and registration number, or state that the review was not registered.	page 04
	24b	Indicate where the review protocol can be accessed, or state that a protocol was not prepared.	page 04
	24c	Describe and explain any amendments to information provided at registration or in the protocol.	NA
Support	25	Describe sources of financial or non-financial support for the review, and the role of the funders or sponsors in the review.	page 01
Competing interests	26	Declare any competing interests of review authors.	page 01
Availability of data, code and other materials	27	Report which of the following are publicly available and where they can be found: template data collection forms; data extracted from included studies; data used for all analyses; analytic code; any other materials used in the review.	page 15

NA: Not applicable

For peer review only

S2 Table: Search terms

S2.1 Table: Embase, Medline and Global Health (OVID)

01	chronic renal insufficiency.mp.
02	chronic kidney disease.mp.
03	chronic kidney failure.mp.
04	CKD.mp.
05	exp Renal Insufficiency, Chronic/
06	(chronic adj2 kidney adj2 disease).mp.
07	(chronic adj2 kidney adj2 failure).mp.
08	chronic renal failure.mp.
09	chronic renal disease.mp.
10	chronic kidney insufficiency.mp.
11	end stage renal disease.mp.
12	ESRD.mp.
13	kidney function.mp.
14	renal function.mp.
15	kidney dysfunction.mp.
16	renal dysfunction.mp.
17	01 or 02 or 03 or 04 or 05 or 06 or 07 or 08 or 09 or 10 or 11 or 12 or 13 or 14 or 15 or 16
18	(("Afghanistan") or ("Benin") or ("Burkina Faso") or ("Burundi") or ("Central African Republic") or ("Chad") or ("Comoros") or ("Democratic Republic of the Congo") or ("Eritrea") or ("Ethiopia") or ("Gambia") or ("Guinea") or ("Guinea-Bissau") or ("Haiti") or ("Democratic People's Republic of Korea") or ("Liberia") or ("Madagascar") or ("Malawi") or ("Mali") or ("Mozambique") or ("Nepal") or ("Niger") or ("Rwanda") or ("Senegal") or ("Sierra Leone") or ("Somalia") or ("South Sudan") or ("Tanzania") or ("Togo") or ("Uganda") or ("Zimbabwe") or ("Armenia") or ("Bangladesh") or ("Bhutan") or ("Bolivia") or ("Cape Verde") or ("Cambodia") or ("Cameroon") or ("Congo") or ("Cote d'Ivoire") or ("Djibouti") or ("Egypt") or ("El Salvador") or ("Ghana") or ("Guatemala") or ("Honduras") or ("India") or ("Indonesia") or ("Kenya") or ("Micronesia") or ("Kosovo") or ("Kyrgyzstan") or ("Laos") or ("Lesotho") or ("Mauritania") or ("Moldova") or ("Mongolia") or ("Morocco") or ("Myanmar") or ("Nicaragua") or ("Nigeria") or ("Pakistan") or ("Papua New Guinea") or ("Philippines") or ("Samoa") or ("Atlantic Islands") or ("Melanesia") or ("Sri Lanka") or ("Sudan") or ("Swaziland") or ("Syria") or ("Tajikistan") or ("Timor-Leste") or ("Tonga") or ("Tunisia") or ("Ukraine") or ("Uzbekistan") or ("Vanuatu") or ("Vietnam") or ("Middle East") or ("Yemen") or ("Zambia") or ("Albania") or ("Algeria") or ("American Samoa") or ("Angola") or ("Argentina") or ("Azerbaijan") or ("Republic of Belarus") or ("Belize") or ("Bosnia and Herzegovina") or ("Botswana") or ("Brazil") or ("Bulgaria") or ("China") or ("Colombia") or ("Costa Rica") or ("Cuba") or ("Dominica") or ("Dominican Republic") or ("Equatorial Guinea") or ("Ecuador") or ("Fiji") or ("Gabon") or ("Georgia") or ("Grenada") or ("Guyana") or ("Iran") or ("Iraq") or ("Jamaica") or ("Jordan") or ("Kazakhstan") or ("Lebanon") or ("Libya") or ("Macedonia") or ("Malaysia") or ("Indian Ocean Islands") or ("Mexico") or ("Montenegro") or ("Namibia") or ("Palau") or ("Panama") or ("Paraguay") or ("Peru") or ("Russia") or ("Serbia") or ("South Africa") or ("Saint Lucia") or ("Saint Vincent and the Grenadines") or ("Suriname") or ("Thailand") or ("Turkey") or ("Turkmenistan") or ("Venezuela") or (developing countr*) or (lowincome countr*) or (middle-income countr*) or (low-middle income countr*) or (upper-middle income countr*))
19	risk assessment.mp.
20	risk functions.mp.
21	Risk Assessment/mt
22	risk equation$.mp.
23	risk chart?.mp.
24	(risk adj3 tool$).mp.
25	risk assessment function?.mp.
26	risk assessor.mp.
27	risk appraisal$.mp.
28	risk calculation$.mp.
29	risk calculator$.mp.

For peer review only - http://bmjopen.bmj.com/site/about/guidelines.xhtml

30	risk factor$ calculator$.mp.
31	risk factor$ calculation$.mp.
32	risk engine$.mp.
33	risk equation$.mp.
34	risk table$.mp.
35	risk threshold$.mp.
36	risk disc?.mp.
37	risk disk?.mp.
38	risk scoring method?.mp.
39	scoring scheme?.mp.
40	risk scoring system?.mp.
41	risk scal$.mp.
42	risk prediction?.mp.
43	risk algorith$.mp.
44	prediction model$.mp.
45	predictive instrument?.mp.
46	project$ risk?.mp.
47	predictive model?.mp.
48	scoring method$.mp.
49	(prediction$ adj3 method$).mp.
50	exp Risk Assessment/
51	(risk? adj1 assess$).mp.
52	screening.mp.
53	diagnostic test.mp.
54	19 or 20 or 21 or 22 or 23 or 24 or 25 or 26 or 27 or 28 or 29 or 30 or 31 or 32 or 33 or 34 or 35 or 36 or 37 or 38 or 39 or 40 or 41 or 42 or 43 or 44 or 45 or 46 or 47 or 48 or 49 or 50 or 51 or 52 or 53
55	17 and 18 and 54
56	exp animals/ not humans.sh.
57	55 not 56
58	Remove duplicates from 57

S2.2 Table: SCOPUS

((TITLE-ABS-KEY("Afghanistan") OR TITLE-ABS-KEY("Benin") OR TITLE-ABS-KEY("Burkina Faso") OR TITLE-ABS-KEY("Burundi") OR TITLE-ABS-KEY("Central African Republic") OR TITLE-ABS-KEY("Chad") OR TITLE-ABS-KEY("Comoros") OR TITLE-ABS-KEY("Democratic Republic of the Congo") OR TITLE ABSKEY("Eritrea") OR TITLE-ABS-KEY("Ethiopia") OR TITLE-ABS-KEY("Gambia") OR TITLE ABSKEY("Guinea") OR TITLE-ABS-KEY("Guinea-Bissau") OR TITLE-ABS-KEY("Haiti") OR TITLE ABSKEY("Democratic People's Republic of Korea") OR TITLE-ABS-KEY("Liberia") OR TITLE ABSKEY("Madagascar") OR TITLE-ABS-KEY("Malawi") OR TITLE-ABS-KEY("Mali") OR TITLE ABSKEY("Mozambique") OR TITLE-ABS-KEY("Nepal") OR TITLE-ABS-KEY("Niger") OR TITLE-ABS-KEY("Rwanda") OR TITLE-ABS-KEY("Senegal") OR TITLE-ABS-KEY("Sierra Leone") OR TITLE-ABSKEY("Somalia") OR TITLE-ABS-KEY("South Sudan") OR TITLE-ABS-KEY("Tanzania") OR TITLE-ABSKEY("Togo") OR TITLE-ABS-KEY("Uganda") OR TITLE-ABS-KEY("Zimbabwe") OR TITLE-ABS-KEY("Armenia") OR TITLE-ABS-KEY("Bangladesh") OR TITLE-ABS-KEY("Bhutan") OR TITLE-ABSKEY("Bolivia") OR TITLE-ABS-KEY("Cape Verde") OR TITLE-ABS-KEY("Cambodia") OR TITLE-ABSKEY("Cameroon") OR TITLE-ABS-KEY("Congo") OR TITLE-ABS-KEY("Cote d'Ivoire") OR TITLE-ABSKEY("Djibouti") OR TITLE-ABS-KEY("Bolivia") OR TITLE-ABS-KEY("Cape Verde") OR TITLE-ABS-KEY("Cambodia") OR TITLE-ABS-KEY("Cameroon") OR TITLE-ABS-KEY("Congo") OR TITLE-ABSKEY("Cote d'Ivoire") OR TITLE-ABS-KEY("Djibouti") OR TITLE-ABS-KEY("Egypt") OR TITLE-ABS-KEY("El Salvador") OR TITLE-ABS-KEY("Ghana") OR TITLE-ABS-KEY("Guatemala") OR TITLE-ABSKEY("Honduras") OR TITLE-ABS-KEY("India") OR TITLE-ABS-KEY("Indonesia") OR TITLE-ABSKEY("Kenya") OR TITLE-ABS-KEY("Micronesia") OR TITLE-ABS-KEY("Kosovo") OR TITLE-ABSKEY("Kyrgyzstan") OR TITLE-ABS-KEY("Laos") OR TITLE-ABS-KEY("Lesotho") OR TITLE-ABS-KEY("Mauritania") OR TITLE-ABS-KEY("Moldova") OR TITLE-ABS-KEY("Mongolia") OR TITLE-ABSKEY("Morocco") OR TITLE-

For peer review only - http://bmjopen.bmj.com/site/about/guidelines.xhtml

ABS-KEY("Myanmar") OR TITLE-ABS-KEY("Nicaragua") OR TITLE-ABS-KEY("Nigeria") OR TITLE-ABS-KEY("Pakistan") OR TITLE-ABS-KEY("Papua New Guinea") OR TITLE-ABSKEY("Philippines") OR TITLE-ABS-KEY("Samoa") OR TITLE-ABS-KEY("Atlantic Islands") OR TITLE-ABSKEY("Melanesia") OR TITLE-ABS-KEY("Sri Lanka") OR TITLE-ABS-KEY("Sudan") OR TITLE-ABSKEY("Swaziland") OR TITLE-ABS-KEY("Syria") OR TITLE-ABS-KEY("Tajikistan") OR TITLE-ABSKEY("Timor-Leste") OR TITLE-ABS-KEY("Tonga") OR TITLE-ABS-KEY("Tunisia") OR TITLE-ABSKEY("Ukraine") OR TITLE-ABS-KEY("Uzbekistan") OR TITLE-ABS-KEY("Vanuatu") OR TITLE-ABSKEY("Vietnam") OR TITLE-ABS-KEY("Middle East") OR TITLE-ABS-KEY("Yemen") OR TITLE-ABS-KEY("Zambia") OR TITLE-ABS-KEY("Albania") OR TITLE-ABS-KEY("Algeria") OR TITLE-ABSKEY("American Samoa") OR TITLE-ABS-KEY("Angola") OR TITLE-ABS-KEY("Argentina") OR TITLE-ABSKEY("Azerbaijan") OR TITLE-ABS-KEY("Republic of Belarus") OR TITLE-ABS-KEY("Belize") OR TITLE-ABSKEY("Bosnia and Herzegovina") OR TITLE-ABS-KEY("Botswana") OR TITLE-ABS-KEY("Brazil") OR TITLEABS-KEY("Bulgaria") OR TITLE-ABS-KEY("China") OR TITLE-ABS-KEY("Colombia") OR TITLE-ABS-KEY("Costa Rica") OR TITLE-ABS-KEY("Cuba") OR TITLE-ABS-KEY("Dominica") OR TITLE-ABSKEY("Dominican Republic") OR TITLE-ABS-KEY("Equatorial Guinea") OR TITLE-ABS-KEY("Ecuador") OR TITLE-ABS-KEY("Fiji") OR TITLE-ABS-KEY("Gabon") OR TITLE-ABS-KEY("Georgia") OR TITLE-ABSKEY("Grenada") OR TITLE-ABS-KEY("Guyana") OR TITLE-ABS-KEY("Iran") OR TITLE-ABS-KEY("Iraq") OR TITLE-ABS-KEY("Jamaica") OR TITLE-ABS-KEY("Jordan") OR TITLE-ABS-KEY("Kazakhstan") OR TITLEABS-KEY("Lebanon") OR TITLE-ABS-KEY("Libya") OR TITLE-ABS-KEY("Macedonia (Republic)") OR TITLEABS-KEY("Malaysia") OR TITLE-ABS-KEY("Indian Ocean Islands") OR TITLE-ABS-KEY("Mexico") OR TITLE-ABS-KEY("Montenegro") OR TITLE-ABS-KEY("Namibia") OR TITLE-ABS-KEY("Palau") OR TITLEABS-KEY("Panama") OR TITLE-ABS-KEY("Paraguay") OR TITLE-ABS-KEY("Peru") OR TITLE-ABSKEY("Russia") OR TITLE-ABS-KEY("Serbia") OR TITLE-ABS-KEY("South Africa") OR TITLE-ABS-KEY("Saint Lucia") OR TITLE-ABS-KEY("Saint Vincent and the Grenadines") OR TITLE-ABS-KEY("Suriname") OR TITLE-ABS-KEY("Thailand") OR TITLE-ABS-KEY("Turkey") OR TITLE-ABS-KEY("Turkmenistan") OR TITLEABS-KEY("Venezuela") OR TITLE-ABS-KEY(developing countr*) OR TITLE-ABS-KEY(lowincome countr*) OR TITLE-ABS-KEY(middle-income countr*) OR TITLE-ABS-KEY(low-middle income countr*) OR TITLEABS-KEY(upper-middle income countr*) OR TITLE-ABS-KEY(“low resource") OR TITLE-ABS-KEY ("underresourced") OR TITLE-ABS-KEY("resource poor") OR TITLE-ABS-KEY("under-developed") OR TITLE-ABSKEY("underdeveloped") OR TITLE-ABS-KEY("developing world") OR TITLE-ABS-KEY(“third world”) OR TITLE-ABS-KEY(lmic) OR TITLE-ABS-KEY(low AND middle AND income)) AND (TITLE-ABS-KEY(Risk Assessment) OR TITLE-ABS-KEY(risk? adj1 assess*) OR TITLE-ABS-KEY(risk function) OR TITLE-ABS-KEY(Risk Assessment) OR TITLE-ABS-KEY(risk functions) OR TITLE-ABS-KEY(risk equation*) OR TITLEABS-KEY(risk chart?) OR TITLE-ABS-KEY(risk adj3 tool*) OR TITLE-ABS-KEY(risk assessment function?) OR TITLE-ABS-KEY(risk assessor) OR TITLE-ABS-KEY(risk appraisal*) OR TITLE-ABS-KEY(risk calculation*) OR TITLE-ABS-KEY(risk calculator*) OR TITLE-ABS-KEY(risk factor* calculator*) OR TITLEABS-KEY(risk factor* calculation*) OR TITLE-ABS-KEY(risk engine*) OR TITLE-ABS-KEY(risk equation*) OR TITLE-ABS-KEY(risk table*) OR TITLE-ABS-KEY(risk threshold*) OR TITLE-ABS-KEY(risk disc?) OR TITLE-ABS-KEY(risk disk?) OR TITLE-ABS-KEY(risk scoring method?) OR TITLE-ABS-KEY(scoring scheme?) OR TITLE-ABS-KEY(risk scoring system?) OR TITLE-ABS-KEY(risk prediction?) OR TITLE-ABSKEY(risk algorith*) OR TITLE-ABS-KEY(prediction model*) OR TITLE-ABS-KEY(predictive instrument?) OR TITLE-ABS-KEY(project* risk?) OR TITLE-ABS-KEY(predictive model?) OR TITLE-ABS-KEY(scoring method*) OR TITLE-ABS-KEY(prediction* adj3 method*) OR TITLE-ABS-KEY(screening) OR TITLE-ABSKEY(risk scal*) OR TITLE-ABS-KEY(diagnostic test)) AND (TITLE-ABS-KEY(chronic renal insufficiency) OR TITLE-ABS-KEY(chronic kidney disease) OR TITLE-ABS-KEY(chronic kidney failure) OR TITLE-ABS-KEY(CKD) OR TITLE-ABS-KEY(chronic renal failure) OR TITLE-ABS-KEY(chronic renal disease) OR TITLE-ABS-KEY(chronic kidney insufficiency) OR TITLE-ABS-KEY(end stage renal disease) OR TITLE-ABSKEY(ESRD) OR TITLE-ABS-KEY(kidney function) OR TITLE-ABS-KEY(renal function) OR TITLE-ABSKEY(kidney dysfunction) OR TITLE-ABS-KEY(renal dysfunction) OR TITLE-ABS-KEY(chronic W/2 kidney W/2 disease) OR TITLE- ABS-KEY(chronic W/2 kidney W/2 failure) AND NOT DBCOLL(medl))

For peer review only - http://bmjopen.bmj.com/site/about/guidelines.xhtml

S2.3 Table: WEB OF SCIENCE

(((chronic renal insufficiency) OR (chronic kidney disease) OR (chronic kidney failure) OR (CKD) OR (Renal Insufficiency, Chronic) OR (chronic NEAR/2 kidney NEAR/2 disease) OR (chronic NEAR/2 kidney NEAR/2 failure) OR (chronic renal failure) OR (chronic renal disease) OR (chronic kidney insufficiency) OR (end stage renal disease) OR (ESRD) OR (kidney function) OR (renal function) OR (kidney dysfunction) OR (renal dysfunction)) AND (("Afghanistan") OR ("Benin") OR ("Burkina Faso") OR ("Burundi") OR ("Central African Republic") OR ("Chad") OR ("Comoros") OR ("Democratic Republic of the Congo") OR ("Eritrea") OR ("Ethiopia") OR ("Gambia") OR ("Guinea") OR ("Guinea-Bissau") OR ("Haiti") OR ("Democratic People's Republic of Korea") OR ("Liberia") OR ("Madagascar") OR ("Malawi") OR ("Mali") OR ("Mozambique") OR ("Nepal") OR ("Niger") OR ("Rwanda") OR ("Senegal") OR ("Sierra Leone") OR ("Somalia") OR ("South Sudan") OR ("Tanzania") OR ("Togo") OR ("Uganda") OR ("Zimbabwe") OR ("Armenia") OR ("Bangladesh") OR ("Bhutan") OR ("Bolivia") OR ("Cape Verde") OR ("Cambodia") OR ("Cameroon") OR ("Congo") OR ("Cote d'Ivoire") OR ("Djibouti") OR ("Egypt") OR ("El Salvador") OR ("Ghana") OR ("Guatemala") OR ("Honduras") OR ("India") OR ("Indonesia") OR ("Kenya") OR ("Micronesia") OR ("Kosovo") OR ("Kyrgyzstan") OR ("Laos") OR ("Lesotho") OR ("Mauritania") OR ("Moldova") OR ("Mongolia") OR ("Morocco") OR ("Myanmar") OR ("Nicaragua") OR ("Nigeria") OR ("Pakistan") OR ("Papua New Guinea") OR ("Philippines") OR ("Samoa") OR ("Atlantic Islands") OR ("Melanesia") OR ("Sri Lanka") OR ("Sudan") OR ("Swaziland") OR ("Syria") OR ("Tajikistan") OR ("Timor-Leste") OR ("Tonga") OR ("Tunisia") OR ("Ukraine") OR ("Uzbekistan") OR ("Vanuatu") OR ("Vietnam") OR ("Middle East") OR ("Yemen") OR ("Zambia") OR ("Albania") OR ("Algeria") OR ("American Samoa") OR ("Angola") OR ("Argentina") OR ("Azerbaijan") OR ("Republic of Belarus") OR ("Belize") OR ("Bosnia and Herzegovina") OR ("Botswana") OR ("Brazil") OR ("Bulgaria") OR ("China") OR ("Colombia") OR ("Costa Rica") OR ("Cuba") OR ("Dominica") OR ("Dominican Republic") OR ("Equatorial Guinea") OR ("Ecuador") OR ("Fiji") OR ("Gabon") OR ("Georgia") OR ("Grenada") OR ("Guyana") OR ("Iran") OR ("Iraq") OR ("Jamaica") OR ("Jordan") OR ("Kazakhstan") OR ("Lebanon") OR ("Libya") OR ("Macedonia (Republic) ") OR ("Malaysia") OR ("Indian Ocean Islands") OR ("Mexico") OR ("Montenegro") OR ("Namibia") OR ("Palau") OR ("Panama") OR ("Paraguay") OR ("Peru") OR ("Russia") OR ("Serbia") OR ("South Africa") OR ("Saint Lucia") OR ("Saint Vincent and the Grenadines") OR ("Suriname") OR ("Thailand") OR ("Turkey") OR ("Turkmenistan") OR ("Venezuela") OR (developing countr) OR (lowincome countr*) OR (middle-income countr*) OR (low-middle income countr*) OR (upper-middle income countr*)) AND ((risk assessment) OR (risk equation$) OR (risk chart?) OR (risk NEAR/3 tool$) OR (risk assessment function?) OR (risk assessor) OR (risk appraisal$) OR (risk calculation$) OR (risk calculator$) OR (risk factor$ calculation$) OR (risk engine$) OR (risk equation$) OR (risk table$) OR (risk threshold$) OR (risk disc?) OR (risk disk?) OR (risk scoring method?) OR (scoring scheme?) OR (risk scoring system?) OR (risk scal$) OR (risk prediction?) OR (risk algorith$) OR (prediction model$) OR (predictive instrument?) OR (project$ risk?) OR (predictive model?) OR (scoring method$) OR (prediction$ NEAR/3 method$) OR (risk? NEAR/1 assess$) OR (screening) OR (diagnostic test))) NOT ((animal*) OR ("not humans"))

For peer review only

S3 Table: **Data extraction form (by chapters)**

S3.1 Table: **Source of data and participants**

		Source of data	Participants									
N°	Study	Source of data	Participant location	Baseline year	End year (cohorts)	Sampling	Inclusion criteria	Exclusion criteria	Outcome prevalence (%)	Outcome incidence (for cohorts)	Baseline mean age	Baseline % men
1	Asgari, 2020 European Risk Assessment tool (6-years validation)	Cohort	Community	1999-2005	2011	Random	Tehran lipids and glucose study (TLGS) cohort participants.	Persons with prevalent Cardiovascular Disease (CVD), Type 2 Diabetes Mellitus or End-stage Renal Disease with (eGFR) <15 mL/min/1.73 m2. Also excluded those with missing data at baseline for creatinine (Cr), fasting plasma glucose (FPG), 2-hour postchallenge plasma	46.02 (11.95)	40.1%	58.34	29.53

For peer review only - http://bmjopen.bmj.com/site/about/guidelines.xhtml

								glucose (2 h-PCG), body mass index (BMI), waist circumference (WC) and smoking status as well as participants with missing data during follow-up on Cr, FPG, 2 h-PCG and CVD status				
1	Asgari, 2020 European Risk Assessment tool (9-years validation)	Cohort	Community	1999-2005	2009-2018	Random	Tehran lipids and glucose study (TLGS) cohort participants.	Persons with prevalent Cardiovascular Disease (CVD), Type 2 Diabetes Mellitus or End-stage Renal Disease with (eGFR) <15 mL/min/1.73 m2. Also excluded those with missing data at baseline for creatinine (Cr), fasting plasma glucose (FPG), 2-hour postchallenge plasma glucose (2 h-PCG), body mass index (BMI), waist circumference (WC) and smoking status as well as participants with missing data during follow-up on Cr, FPG, 2 h-PCG and CVD status	NI	40.6%	48.20	49.70
2	Bradshaw, 2019 - Model 1 (derivation)	Cross-sectional	Community	2015	n/a	Random	Any individual aged ≥20 years and permanently residingin at Delhi and Chennai (CARRS-II). A permanent resident was defined as a person living in the selected household, was related to the household head and ate at least 3 meals in a week with the family.	Beddriden individuals, pregnant women, participants with missing both or either serum creatinine or urine albumin-to- creatinine ratio data and participants on dialysis.	44.9 (13.5)	46.8%	48.20	49.70

For peer review only - http://bmjopen.bmj.com/site/about/guidelines.xhtml

							Households were defined as "a group of people wholive together, usually pool their income and eat atleast one meal together a day when they are at home. This does not include people who have migratedpermanently or are considered visitors"					
2	Bradshaw, 2019 - Model 2 (derivation)	Cross-sectional	Community	2015	n/a	Random	Any individual aged ≥20 years and permanently residingin at Delhi and Chennai (CARRS-II). A permanent resident was defined as a person living in the selected household, was related to the household head and ate at least 3 meals in a week with the family. Households were defined as "a group of people wholive together, usually pool their income and eat atleast one meal together a day when they are at home. This does not include people who have migratedpermanently or are considered visitors"	Beddriden individuals, pregnant women, participants with missing both or either serum creatinine or urine albumin-to- creatinine ratio data and participants on dialysis.	44.9 (13.5)	46.8%	48.20	49.70
2	Bradshaw, 2019 - Model 3a (derivation)	Cross-sectional	Community	2015	n/a	Random	Any individual aged ≥20 years and permanently residingin at Delhi and Chennai (CARRS-II). A permanent resident was defined as a person living in the selected household,	Beddriden individuals, pregnant women, participants with missing both or either serum creatinine or urine albumin-to- creatinine ratio data and participants on dialysis.	44.9 (13.5)	46.8%	39.90	46.97

For peer review only - http://bmjopen.bmj.com/site/about/guidelines.xhtml

							was related to the household head and ate at least 3 meals in a week with the family. Households were defined as “a group of people wholive together, usually pool their income and eat atleast one meal together a day when they are at home. This does not include people who have migratedpermanently or are considered visitors”					
2	Bradshaw, 2019 - Model 3b (derivation)	Cross-sectional	Community	2015	n/a	Random	Any individual aged ≥20 years and permanently residingin at Delhi and Chennai (CARRS-II). A permanent resident was defined as a person living in the selected household, was related to the household head and ate at least 3 meals in a week with the family. Households were defined as “a group of people wholive together, usually pool their income and eat atleast one meal together a day when they are at home. This does not include people who have migratedpermanently or are considered visitors”	Beddriden individuals, pregnant women, participants with missing both or either serum creatinine or urine albumin-to- creatinine ratio data and participants on dialysis.	44.9 (13.5)	46.8%	39.90	46.97
2	Bradshaw, 2019 - Model	Cross-sectional	Community	2010-2012	n/a	Random	Any individual aged ≥20 years and permanently residingin at Delhi	Beddriden individuals, pregnant women, participants with missing	NI	NI	47.20	38.00

For peer review only - http://bmjopen.bmj.com/site/about/guidelines.xhtml

	3a (CARRS -I urban validatio n)						(CARRS-I). A permanent resident was defined as a person living in the selected household, was related to the household head and ate at least 3 meals in a week with the family. Households were defined as “a group of people wholive together, usually pool their income and eat atleast one meal together a day when they are at home. This does not include people who have migratedpermanently or are considered visitors”	both or either serum creatinine or urine albumin-to- creatinine ratio data and participants on dialysis.				
2	Bradsha w, 2019 - Model 3a (UDAY rural validatio n)	Cross-sectional	Communit y	2014	n/a	Random	UDAY cohort participants ((a) adults aged ≥30 years residing in the sampled urban and rural areas of Sonipat and Vizag, respectively; and (b) willing to participate and provide informed consent).	Participants with missing both or either serum creatinine or urine albumin-to- creatinine ratio data, unwilling to provide informed consent, with serious chronic illnesses [such as that of the liver (cirrhosis), kidneys (renal failure) or malignancies], and pregnant women.	NI	NI	47.20	38.00
3	Carrillo-Larco, 2017 - CRONI CAS-CKD (derivati on	Cross-sectional	Communit y	2013-2014	n/a	Random	Full time resident, capable of giving informed consent, one subject per household.	Being pregnant, having active pulmonary tuberculosis, and having any disability preventing from undergoing anthropometric assessments, having CKD, missing values in the prediction variables, missing	57.7 (12.4)	49.4%		

For peer review only - http://bmjopen.bmj.com/site/about/guidelines.xhtml

	complete)							values in key variables to calculate eGFR, subjects with BMI >40 kg/m2 or BMI <18.5 kg/m2.				
3	Carrillo-Larco, 2017 - CRONICAS-CKD (derivation lab-free)	Cross-sectional	Community	2013-2014	n/a	Random	Full time resident, capable of giving informed consent, one subject per household.	Being pregnant, having active pulmonary tuberculosis, and having any disability preventing from undergoing anthropometric assessments, having CKD, missing values in the prediction variables, missing values in key variables to calculate eGFR, subjects with BMI >40 kg/m2 or BMI <18.5 kg/m2.	57.7 (12.4)	49.4%		
3	Carrillo-Larco, 2017 - CRONICAS-CKD (validation complete)	Cross-sectional	Community	2004-2006	n/a	Random	PREVENCION cohort participants.	Report having CKD, missing values in key variables to calculate eGFR, subjects with BMI >40 kg/m2 or BMI <18.5 kg/m2, age < 35 years, missing values in prediction variables.	57.1 (12.6)	47.7%		
3	Carrillo-Larco, 2017 - CRONICAS-CKD (validation lab-free)	Cross-sectional	Community	2004-2006	n/a	Random	PREVENCION cohort participants.	Report having CKD, missing values in key variables to calculate eGFR, subjects with BMI >40 kg/m2 or BMI <18.5 kg/m2, age < 35 years, missing values in prediction variables.	57.1 (12.6)	47.7%		
4	Mogueo, 2015 -	Cross-sectional	Community	2008-2011	n/a	Random	Cape Town Bellville-South study cohort participants.	Participants with missing data on all variables, except	55 (15)	23.4%		

For peer review only - http://bmjopen.bmj.com/site/about/guidelines.xhtml

	Korean model (eGFR validation)							anaemia				
4	Mogueo, 2015 - Thai model (eGFR validation)	Cross-sectional	Community	2008-2011	n/a	Random	Cape Town Bellville-South study cohort participants.	Participants with missing data on all variables, except kidney stones	55 (15)	23.4%		
4	Mogueo, 2015 - Korean model (eGFR or proteinuria validation)	Cross-sectional	Community	2008-2011	n/a	Random	Cape Town Bellville-South study cohort participants.	Participants with missing data on all variables, except anaemia	55 (15)	23.4%		
4	Mogueo, 2015 - Thai model (eGFR or proteinuria validation)	Cross-sectional	Community	2008-2011	n/a	Random	Cape Town Bellville-South study cohort participants.	Participants with missing data on all variables, except kidney stones	55 (15)	23.4%		
5	Saranburut, 2017 - Framingham Heart	Cohort	Community	2002	2012	Random	Employees of the Electric Generating Authority of Thailand (EGAT) who participated in a health survey in 2002	Subjects who had CKD at baseline or did not have serum creatinine at baseline or at follow-up.	54.6 (5.6)	70.8%		

For peer review only

	Study (MDRD validatio n)											
5	Saranbu rut, 2017 - Framing ham Heart Study (CKD-EPI validatio n)	Cohort	Communit y	2002	2012	Random	Employees of the Electric Generating Authority of Thailand (EGAT) who participated in a health survey in 2003	Subjects who had CKD at baseline or did not have serum creatinine at baseline or at follow-up.	54.7 (5.7)	71.5%		
6	Saranbu rut, 2017 - Model 1 (derivati on Clinical only)	Cohort	Communit y	2002-2003	2012-2013	Random	EGAT 1-2 cohort participants with preserved GFR (estimate glomerular filtration rate (eGFR) ≥ 60 mL/min/1.73m2) at baseline who attended both the examinations (EGAT 1 5rd examination and EGAT 2 4nd examination).	Patients who died, retired, moved, did not want to participate o had with missing baseline serum creatinine data. Also, patients with eGFR<60 at baseline in 2002-2003 were excluded	51.3 (7.4)	70.5%		
6	Saranbu rut, 2017 - Model 1 BMI (derivati on Clinical only)	Cohort	Communit y	2002-2003	2012-2013	Random	EGAT 1-2 cohort participants with preserved GFR (estimate glomerular filtration rate (eGFR) ≥ 60 mL/min/1.73m2) at baseline who attended both the examinations (EGAT 1 5rd examination and EGAT 2 4nd examination).	Patients who died, retired, moved, did not want to participate o had with missing baseline serum creatinine data. Also, patients with eGFR<60 at baseline in 2002-2003 were excluded	51.3 (7.4)	70.5%		

For peer review only - http://bmjopen.bmj.com/site/about/guidelines.xhtml

6	Saranburut, 2017 - Model 2 (derivation Clinical + Limited laboratory tests)	Cohort	Community	2002-2003	2012-2013	Random	EGAT 1-2 cohort participants with preserved GFR (estimate glomerular filtration rate (eGFR) ≥ 60 mL/min/1.73m2) at baseline who attended both the examinations (EGAT 1 5rd examination and EGAT 2 4nd examination).	Patients who died, retired, moved, did not want to participate o had with missing baseline serum creatinine data. Also, patients with eGFR<60 at baseline in 2002-2003 were excluded	51.3 (7.4)	70.5%		
6	Saranburut, 2017 - Model 3 (derivation Clinical + Full laboratory tests)	Cohort	Community	2002-2003	2012-2013	Random	EGAT 1-2 cohort participants with preserved GFR (estimate glomerular filtration rate (eGFR) ≥ 60 mL/min/1.73m2) at baseline who attended both the examinations (EGAT 1 5rd examination and EGAT 2 4nd examination).	Patients who died, retired, moved, did not want to participate o had with missing baseline serum creatinine data. Also, patients with eGFR<60 at baseline in 2002-2003 were excluded	51.3 (7.4)	70.5%		
6	Saranburut, 2017 - Model 1 (validation Clinical only)	Cohort	Community	2009	2014	Random	EGAT 3 cohort participants with preserved GFR (eGFR ≥ 60) at baseline in 2009 (EGAT 3 1st examination) who were followed up 5 years later in 2014 (EGAT 3 2nd examination).	Participants younger than 40 years old at baseline, with missing serum creatinine values, parrients who died, retired and moved, unwilling to participate and with an eGFR <60 at baseline.	45.6 (4.2)	75.5%		
6	Saranburut, 2017 - Model 2 (validation Clinical +	Cohort	Community	2009	2014	Random	EGAT 3 cohort participants with preserved GFR (eGFR ≥ 60) at baseline in 2009 (EGAT 3 1st examination) who were followed up 5 years later in 2014 (EGAT 3 2nd examination).	Participants younger than 40 years old at baseline, with missing serum creatinine values, parrients who died, retired and moved, unwilling to participate and with an eGFR <60 at baseline.	45.6 (4.2)	75.5%		

For peer review only - http://bmjopen.bmj.com/site/about/guidelines.xhtml

	Limited laboratory tests)											
7	Thakkinstian, 2011 (derivation)	Cross-sectional	Community	2007-2008	n/a	Random	Global Screening and Early Evaluation of Kidney Disease (SEEK) study subjects: being 18 years or older, had no menstruation period for at least a week prior to the examination date if women, and whom were willing participants of the study and provided signed consent forms.	Subjects without blood or urine specimens.	45.2 (0.79)	45.5%		
8	Wen, 2020 - Simple Risk Score (derivation)	Cohort	Community	2006-2007	2012-2013	Random	Handan Eye Study (HES) participants (rural residents aged ≥30 years old living in Yongnian County).	Subjects who were diagnosed with CKD, unwilling to participate, missing follow up data (eGFR or UACR).	50 (10)	44.7%		
8	Wen, 2020 - Best-fit Risk Score (derivation)	Cohort	Community	2006-2007	2012-2013	Random	Handan Eye Study (HES) participants (rural residents aged ≥30 years old living in Yongnian County).	Subjects who were diagnosed with CKD, unwilling to participate, missing follow up data (eGFR or UACR).	50 (10)	44.7%		
9	Wu, 2016 (derivation)	Cross-sectional	Community	2012	n/a	Random	Adults older than 18 years and having given consent to this study.	Participants without: age information; body mass index (BMI) information; blood pressure (BP) measurement; serum creatinine test.	45.3 (14.3)	56.7%		
9	Wu, 2016	Cross-sectional	Community	2012	n/a	Random	Adults older than 18 years and having given consent to this study.	Participants without: age information; body mass index (BMI) information;	41.8 (11.7)	63.7%		

For peer review only

	(validati on)							blood pressure (BP) measurement; serum creatinine test.				

For peer review only

S3.2 Table:: Outcome

		Outcome					
N°	Study	Outcome	Outcome details	Same outcome definition for all patients?	Blinded outcome	Predictors part of the outcome	Mean follow-up (years) (cohorts)
1	Asgari, 2020 European Risk Assessment tool (6-years validation)	CKD composite	CKD was defined as eGFR < 60 mL/min/1.73 m2, provided by the Modification of Diet in Renal Disease (MDRD).	Yes	NI	No	6.2
1	Asgari, 2020 European Risk Assessment tool (9-years validation)	CKD composite	CKD was defined as eGFR < 60 mL/min/1.73 m2, provided by the Modification of Diet in Renal Disease (MDRD).	Yes	NI	No	9.2
2	Bradshaw, 2019 - Model 1 (derivation)	CKD composite	CKD was defined as an eGFR rate <60 mL/min/1.73 m2 (estimated by the CKD-EPI equation) or UACR ≥30 mg/g	Yes	NI	No	0
2	Bradshaw, 2019 - Model 2 (derivation)	CKD composite	CKD was defined as an eGFR rate <60 mL/min/1.73 m2 (estimated by the CKD-EPI equation) or UACR ≥30 mg/g	Yes	NI	No	0
2	Bradshaw, 2019 - Model 3a (derivation)	CKD composite	CKD was defined as an eGFR rate <60 mL/min/1.73 m2 (estimated by the CKD-EPI equation) or UACR ≥30 mg/g	Yes	NI	No	0
2	Bradshaw, 2019 - Model 3b (derivation)	CKD composite	CKD was defined as an eGFR rate <60 mL/min/1.73 m2 (estimated by the CKD-EPI equation) or UACR ≥30 mg/g	Yes	NI	No	0
2	Bradshaw, 2019 - Model 3a	CKD composite	CKD was defined as an eGFR rate <60 mL/min/1.73 m2 (estimated by the CKD-EPI equation) or UACR ≥30 mg/g	Yes	NI	No	0

For peer review only - http://bmjopen.bmj.com/site/about/guidelines.xhtml

	(CARRS-I urban validation)						
2	Bradshaw, 2019 - Model 3a (UDAY rural validation)	CKD composite	CKD was defined as an eGFR rate <60 mL/min/1.73 m2 (estimated by the CKD-EPI equation) or UACR ≥30 mg/g	Yes	NI	No	0
3	Carrillo-Larco, 2017 - CRONICAS-CKD (derivation complete)	CKD composite	CKD defined as an eGFR <60 mL/min/1.73m2, using the MDRD (Modification of Diet in Renal Disease) formula, also known as CKD stage III	Yes	Yes	No	0
3	Carrillo-Larco, 2017 - CRONICAS-CKD (derivation lab-free)	CKD composite	CKD defined as an eGFR <60 mL/min/1.73m2, using the MDRD (Modification of Diet in Renal Disease) formula, also known as CKD stage III	Yes	Yes	No	0
3	Carrillo-Larco, 2017 - CRONICAS-CKD (validation complete)	CKD composite	CKD defined as an eGFR <60 mL/min/1.73m2, using the MDRD (Modification of Diet in Renal Disease) formula, also known as CKD stage III	Yes	Yes	No	0
3	Carrillo-Larco, 2017 - CRONICAS-CKD (validation lab-free)	CKD composite	CKD defined as an eGFR <60 mL/min/1.73m2, using the MDRD (Modification of Diet in Renal Disease) formula, also known as CKD stage III	Yes	Yes	No	0
4	Mogueo, 2015 - Korean model (eGFR validation)	CKD composite	eGFR <60 ml/min/1.73 m2 based on the 4-variable Modification of Diet in Renal Disease (MDRD) formula	Yes	NI	No	0
4	Mogueo, 2015 - Thai model (eGFR validation)	CKD composite	eGFR <60 ml/min/1.73 m2 based on the 4-variable Modification of Diet in Renal Disease (MDRD) formula	Yes	NI	No	0
4	Mogueo, 2015 - Korean model (eGFR or	CKD composite	eGFR <60 ml/min/1.73 m2 based on the 4-variable Modification of Diet in Renal Disease (MDRD) formula and 'any nephropathy' including any of the stages I to V of the Kidney Disease: Improving Global Outcomes Chronic	Yes	NI	No	0

For peer review only - http://bmjopen.bmj.com/site/about/guidelines.xhtml

	proteinuria validation)		Kidney Disease (KDIGO) classification				
4	Mogueo, 2015 - Thai model (eGFR or proteinuria validation)	CKD composite	eGFR <60 ml/min/1.73 m2 based on the 4-variable Modification of Diet in Renal Disease (MDRD) formula and ‘any nephropathy’ including any of the stages I to V of the Kidney Disease: Improving Global Outcomes Chronic Kidney Disease (KDIGO) classification	Yes	NI	No	0
5	Saranburut, 2017 - Framingham Heart Study (MDRD validation)	CKD composite	CKD was defined as estimate glomerular filtration rate (eGFR) <60 mL/min/1.73 m2 using the Modification of Diet in Renal Disease (MDRD)	Yes	NI	No	10
5	Saranburut, 2017 - Framingham Heart Study (CKD-EPI validation)	CKD composite	CKD defined as (eGFR) <60 mL/min/1.73 m2 using the CKD-EPI equation.	Yes	NI	No	10
6	Saranburut, 2017 - Model 1 (derivation Clinical only)	CKD composite	Preserved GFR (eGFR ≥60) at baseline and subsequently developed decreased GFR (eGFR < 60 mL/min/1.73m2) at the 10 year follow-up calculated according to two-level race variable Chronic Kidney Disease–Epidemiology Collaboration (CKDEPI) equation using the non-black coefficient. The outcome is a modification from the KDIGO definition of CKD stage 3-5	Yes	NI	No	10
6	Saranburut, 2017 - Model 1 BMI (derivation Clinical only)	CKD composite	Preserved GFR (eGFR ≥60) at baseline and subsequently developed decreased GFR (eGFR < 60 mL/min/1.73m2) at the 10 year follow-up calculated according to two-level race variable Chronic Kidney Disease–Epidemiology Collaboration (CKDEPI) equation using the non-black coefficient. The outcome is a modification from the KDIGO definition of CKD stage 3-5	Yes	NI	No	10
6	Saranburut, 2017 - Model 2 (derivation Clinical + Limited laboratory tests)	CKD composite	Preserved GFR (eGFR ≥60) at baseline and subsequently developed decreased GFR (eGFR < 60 mL/min/1.73m2) at the 10 year follow-up calculated according to two-level race variable Chronic Kidney Disease–Epidemiology Collaboration (CKDEPI) equation using the non-black	Yes	NI	No	10

For peer review only - http://bmjopen.bmj.com/site/about/guidelines.xhtml

For peer review only

			coefficient. The outcome is a modification from the KDIGO definition of CKD stage 3-5				
6	Saranburut, 2017 - Model 3 (derivation Clinical + Full laboratory tests)	CKD composite	Preserved GFR (eGFR ≥60) at baseline and subsequently developed decreased GFR (eGFR < 60 mL/min/1.73m2) at the 10 year follow-up calculated according to two-level race variable Chronic Kidney Disease–Epidemiology Collaboration (CKDEPI) equation using the non-black coefficient. The outcome is a modification from the KDIGO definition of CKD stage 3-5	Yes	NI	No	10
6	Saranburut, 2017 - Model 1 (validation Clinical only)	CKD composite	Preserved GFR (eGFR ≥60) at baseline and subsequently developed decreased GFR (eGFR < 60 mL/min/1.73m2) at the 10 year follow-up calculated according to two-level race variable Chronic Kidney Disease–Epidemiology Collaboration (CKDEPI) equation using the non-black coefficient. The outcome is a modification from the KDIGO definition of CKD stage 3-5	Yes	NI	No	5
6	Saranburut, 2017 - Model 2 (validation Clinical + Limited laboratory tests)	CKD composite	Preserved GFR (eGFR ≥60) at baseline and subsequently developed decreased GFR (eGFR < 60 mL/min/1.73m2) at the 10 year follow-up calculated according to two-level race variable Chronic Kidney Disease–Epidemiology Collaboration (CKDEPI) equation using the non-black coefficient. The outcome is a modification from the KDIGO definition of CKD stage 3-5	Yes	NI	No	5
7	Thakkinstian, 2011 (derivation)	CKD composite	CKD was defined as stage I & II if GFR ≥ 90 and GFR 60-89 ml/min/1.73 m2 with haematuria and/or albumin-creatinine ratio 30 mg/g or greater, stage III, IV, and V if the GFR of 30-59, 15-29, and < 15 ml/min/1.73 m2 respectively, regardless of kidney damage. eGFR was calculated using the MDRD equation for IDMS traceable serum creatinine values.	Yes	NI	No	0
8	Wen, 2020 - Simple Risk Score (derivation)	CKD composite	CKD was defined as an eGFR rate <60 mL/min/1.73 m2 ((assessed by the modified Chinese MDRD equation) or UACR ≥30 mg/g	Yes	NI	No	5.6
8	Wen, 2020 - Best-fit Risk Score (derivation)	CKD composite	CKD was defined as an eGFR rate <60 mL/min/1.73 m2 ((assessed by the modified Chinese MDRD equation) or UACR ≥30 mg/g	Yes	NI	No	5.6

For peer review only - http://bmjopen.bmj.com/site/about/guidelines.xhtml

9	Wu, 2016 (derivation)	CKD composite	Reduced eGFR was defined as eGFR<60 mL/min/1.73 m2 using the CKD-EPI equation.	Yes	NI	No	0
9	Wu, 2016 (validation)	CKD composite	Reduced eGFR was defined as eGFR<60 mL/min/1.73 m2 using the CKD-EPI equation.	Yes	NI	No	0

CKD, chronic kidney disease; CKD-EPI, chronic kidney disease epidemiology collaboration; eGFR, estimated glomerular filtration rate; GFR, glomerular filtration rate; KDIGO, MDRD, modification of diet renal disease; n/a, not applicable; NI, no information; UACR, urinary albumin-to-creatinine ratio.

For peer review only

For peer review only - http://bmjopen.bmj.com/site/about/guidelines.xhtml

S3.3 Table: Candidate predictors

		Candidate Predictors						
N°	Study	Number of candidate predictors	Number of predictors in the final model	Predictors timing	List of predictors in the final model	Predictors definition	Predictors ascertainment	Predictors modelling
1	Asgari, 2020 European Risk Assessment tool (6-years validation)	n/a	18	NI	Age; BMI (body mass index); waist circumference; use of antihypertensives; current smoking, parent and/or sibling with myocardial infarction or stroke; parent and/or sibling with diabetes.	Age (<45, ≥45 to <50, ≥50 to <55, ≥55 to <60, ≥60 to <65, ≥65 to <70, ≥70 to <75, ≥75 to <85); Body mass index (<25, ≥25 to <30, ≥30); Waist circumference [<94, ≥94 to <102, ≥102 (for men) and <80, ≥80 to <88, ≥88 (for women)]; use of antihypertensive medications; current smoking ('who smokes cigarettes daily or occasionally'); family history of cardiovascular disease (CVD) and/or diabetes (previously diagnosed CVD in first-degree male and female relatives aged < 55 and < 65 years, respectively)	BMI was calculated as weight (kg) divided by height (m2). Data collected by trained interviewer using a standard questionnaire	n/a
1	Asgari, 2020 European Risk Assessment tool (9-years validation)	n/a	18	NI	Age; BMI (body mass index); waist circumference; use of antihypertensives; current smoking, parent and/or sibling with myocardial infarction or stroke; parent and/or sibling with diabetes.	Age (<45, ≥45 to <50, ≥50 to <55, ≥55 to <60, ≥60 to <65, ≥65 to <70, ≥70 to <75, ≥75 to <85); Body mass index (<25, ≥25 to <30, ≥30); Waist circumference [<94, ≥94 to <102, ≥102 (for men) and <80, ≥80 to <88, ≥88 (for women)]; use of antihypertensive medications; current smoking ('who smokes cigarettes daily	BMI was calculated as weight (kg) divided by height (m2). Data collected by trained interviewer using a standard questionnaire	n/a

For peer review only - http://bmjopen.bmj.com/site/about/guidelines.xhtml

						or occasionally'); family history of cardiovascular disease (CVD) and/or diabetes (previously diagnosed CVD in first-degree male and female relatives aged < 55 and < 65 years, respectively)		
2	Bradshaw, 2019 - Model 1 (derivation)	30	NI	NI	NI	NI	NI	All continuous variables used cubic spline terms with knots placed at fixed quantiles of the predictor's marginal distribution, categorical variables were summarized using percentages and counts.
2	Bradshaw, 2019 - Model 2 (derivation)	23	NI	NI	NI	NI	NI	All continuous variables used cubic spline terms with knots placed at fixed quantiles of the predictor's marginal

For peer review only - http://bmjopen.bmj.com/site/about/guidelines.xhtml

								distribution, categorical variables were summarized using percentages and counts.
2	Bradshaw, 2019 - Model 3a (derivation)	NI	NI	NI	NI	NI	NI	All continuous variables used cubic spline terms with knots placed at fixed quantiles of the predictor's marginal distribution, categorical variables were summarized using percentages and counts.
2	Bradshaw, 2019 - Model 3b (derivation)	8	NI	NI	NI	NI	NI	All continuous variables used cubic spline terms with knots placed at fixed quantiles of the

For peer review only - http://bmjopen.bmj.com/site/about/guidelines.xhtml

For peer review only

								predictor's marginal distribution, categorical variables were summarized using percentages and counts.
2	Bradshaw, 2019 - Model 3a (CARRS-I urban validation)	n/a	NI	NI	NI	NI	NI	n/a
2	Bradshaw, 2019 - Model 3a (UDAY rural validation)	n/a	NI	NI	NI	NI	NI	n/a
3	Carrillo-Larco, 2017 - CRONICAS-CKD (derivation complete)	36	7	NI	Age; hypertension; anemia.	Age (< 50, 50-69, ≥ 70 years), hypertension (blood pressure ≥ 140/90 mmHg OR previous diagnosis of hypertension and currently under treatment) and anemia (haemoglobin < 13 g/dL if male and < 12 g/dL if female).	Age (information was collected by trained fieldworkers through face-to-face interviews), hypertension (blood pressure measurements were conducted according to the recommendations of the 7th Joint National Committee on the diagnosis and management of High Blood Pressure in adults (JNC-7), NI on anemia.	NI
3	Carrillo-Larco, 2017 -	26	5	NI	Age; hypertension.	Age (< 50, 50-69, ≥ 70 years), hypertension (blood pressure ≥ 140/90 mmHg OR previous diagnosis of	Age (information was collected by trained fieldworkers through face-to-	NI

For peer review only

	CRONICAS-CKD (derivation lab-free)					hypertension and currently under treatment).	face interviews), hypertension (blood pressure measurements were conducted according to the recommendations of the 7th Joint National Committee on the diagnosis and management of High Blood Pressure in adults (JNC-7), NI on anemia.	
3	Carrillo-Larco, 2017 - CRONICAS-CKD (validation complete)	n/a	7	NI	Age; hypertension; anemia.	Age (< 50, 50-69, ≥ 70 years), hypertension (blood pressure ≥ 140/90 mmHg OR previous diagnosis of hypertension and currently under treatment) and anemia (haemoglobin < 13 g/dL if male and < 12 g/dL if female).	Age (information was collected by trained fieldworkers through face-to-face interviews), hypertension (blood pressure measurements were conducted according to the recommendations of the 7th Joint National Committee on the diagnosis and management of High Blood Pressure in adults (JNC-7), NI on anemia.	n/a
3	Carrillo-Larco, 2017 - CRONICAS-CKD (validation lab-free)	n/a	5	NI	Age; hypertension.	Age (< 50, 50-69, ≥ 70 years), hypertension (blood pressure ≥ 140/90 mmHg OR previous diagnosis of hypertension and currently under treatment).	Age (information was collected by trained fieldworkers through face-to-face interviews), hypertension (blood pressure measurements were conducted according to the recommendations of the 7th Joint National Committee on the diagnosis and management of High Blood Pressure in adults (JNC-7), NI on anemia.	n/a
4	Mogueo, 2015 -	n/a	8	NI	Age; sex; diabetes mellitus; hypertension; use	Age (50-59, 60-69, ≥70); Female gender; Hypertension (history of	Participants received a standardized interview (Age	NI

For peer review only - http://bmjopen.bmj.com/site/about/guidelines.xhtml

	Korean model (eGFR validation)				of statins; proteinuria	illness, taking antihyper-tensive drug(s) or had systolic blood pressure ≥140 mmHg or diastolicblood pressure ≥90 mmHg); Diabetes (history of illness, taking oral hypoglycaemicagents or fasting plasma glucose levels≥126 mg/dL); Use of statins; Proteinuria	and sex) and physical examination during which blood pressure was measured according to the World Health Organisation (WHO) guidelines using a semi-automated digital blood pressure monitor (Rossmax PA, USA) on the right arm in the sitting position. Participants with no history of doctor diagnosed diabetes mellitus underwent a 75 g oral glucose tolerance test (OGTT) as recommended by the WHO	
4	Mogueo, 2015 - Thai model (eGFR validation)	n/a	8	NI	Age; diabetes mellitus; hypertension	Age (<40, 40-59, 60-69, >70); Hypertension (history of illness, taking antihyper-tensive drug(s) or had systolic blood pressure ≥140 mmHg or diastolicblood pressure ≥90 mmHg); Diabetes (history of illness, taking oral hypoglycaemicagents or fasting plasma glucose levels≥126 mg/dL)	Participants received a standardized interview (Age) and physical examination during which blood pressure was measured according to the World Health Organisation (WHO) guidelines using a semi-automated digital blood pressure monitor (Rossmax PA, USA) on the right arm in the sitting position. Participants with no history of doctor diagnosed diabetes mellitus underwent a 75 g oral glucose tolerance test (OGTT) as recommended by the WHO	NI
4	Mogueo, 2015 - Korean model (eGFR or	n/a	8	NI	Age; sex; diabetes mellitus; hypertension; use of statins; proteinuria	Age (50-59, 60-69, ≥70); Female gender; Hypertension (history of illness, taking antihyper-tensive drug(s) or had systolic blood pressure ≥140 mmHg or diastolicblood pressure	Participants received a standardized interview (Age and sex) and physical examination during which blood pressure was measured	NI

For peer review only

	proteinuria validation)					≥90 mmHg); Diabetes (history of illness, taking oral hypoglycaemicagents or fasting plasma glucose levels≥126 mg/dL); Use of statins; Proteinuria	according to the World Health Organisation (WHO) guidelines using a semi-automated digital blood pressure monitor (Rossmax PA, USA) on the right arm in the sitting position. Participants with no history of doctor diagnosed diabetes mellitus underwent a 75 g oral glucose tolerance test (OGTT) as recommended by the WHO	
4	Mogueo, 2015 - Thai model (eGFR or proteinuria validation)	n/a	8	NI	Age; diabetes mellitus; hypertension	Age (<40, 40-59, 60-69, >70); Hypertension (history of illness, taking antihyper-tensive drug(s) or had systolic blood pressure ≥140 mmHg or diastolicblood pressure ≥90 mmHg); Diabetes (history of illness, taking oral hypoglycaemicagents or fasting plasma glucose levels≥126 mg/dL)	Participants received a standardized interview (Age) and physical examination during which blood pressure was measured according to the World Health Organisation (WHO) guidelines using a semi-automated digital blood pressure monitor (Rossmax PA, USA) on the right arm in the sitting position. Participants with no history of doctor diagnosed diabetes mellitus underwent a 75 g oral glucose tolerance test (OGTT) as recommended by the WHO	NI
5	Saranburut, 2017 - Framingham Heart Study (MDRD validation)	n/a	5	NI	Diabetes mellitus; hypertension; eGFR category	Diabetes mellitus (yes); hypertension (yes); eGFR category (60-74, 75-89, 90-119)	Hypertension was defined as systolic blood pressure ≥ 140 mmHg or diastolic blood pressure ≥ 90 mmHg or use of oral antihypertensive medication. Diabetes mellitus was defined as a fasting glucose of ≥126 mg/dl or use	n/a

For peer review only - http://bmjopen.bmj.com/site/about/guidelines.xhtml

							of medications. eGFR was estimated using the Modification of Diet in Renal Disease (MDRD) equation.	
5	Saranburut, 2017 - Framingham Heart Study (CKD-EPI validation)	n/a	16	NI	Age; diabetes mellitus; hypertension; eGFR category	Age (30-34, 35-39, 40-44, 45-49, 50-54, 55-59, 60-64, 65-69, 70-74, 75-79, 80-85); diabetes mellitus (yes); hypertension (yes); eGFR category (60-74, 75-89, 90-119)	Age was obtained by a survey. Hypertension was defined as systolic blood pressure ≥ 140 mmHg or diastolic blood pressure ≥ 90 mmHg or use of oral antihypertensive medication. Diabetes mellitus was defined as a fasting glucose of ≥126 mg/dl or use of medications. eGFR was estimated using the chronic kidney disease–epidemiology collaboration (CKD-EPI) equation	n/a
6	Saranburut, 2017 - Model 1 (derivation Clinical only)	15	15	NI	Age; sex; systolic blood pressure; waist circumference; diabetes mellitus	Age (<45, 45-54, 55-59, ≥55); Sex (male, female); Waist circumference (≤80 for male or ≤90 for male, >80 for female or >90 for male); Diabetes (yes, no); Systolic blood pressure (<120, 120-129, 130-139, 140-149, 150-159, ≥160)	Age (health survey), sex (health survey). Hypertension was defined as systolic blood pressure ≥ 140 mmHg or diastolic blood pressure ≥ 90 mmHg or use of oral antihypertensive medication. Diabetes mellitus was defined as a fasting glucose of ≥126 mg/dl or a positive history of diabetes. Waist circumference was measured midway between the lowest ribs and the iliac crest.	NI
6	Saranburut, 2017 - Model 1 BMI (derivation	15	15	NI	Age; sex; systolic blood pressure; body mass index (BMI); diabetes mellitus	Age (<45, 45-54, 55-59, ≥55); Sex (male, female); BMI (<25, ≥25); Diabetes (yes, no); Systolic blood pressure (<120, 120-129, 130-139, 140-149, 150-159, ≥160)	Age (health survey), sex (health survey). Hypertension was defined as systolic blood pressure ≥ 140 mmHg or diastolic blood pressure ≥ 90 mmHg or use of oral	NI

For peer review only - http://bmjopen.bmj.com/site/about/guidelines.xhtml

	Clinical only)						antihypertensive medication. Diabetes mellitus was defined as a fasting glucose of ≥126 mg/dl or a positive history of diabetes. Body mass index was defined as weight in kilograms divided by the square of height in meters	
6	Saranburut, 2017 - Model 2 (derivation Clinical + Limited laboratory tests)	16	16	NI	Age; sex; systolic blood pressure; diabetes mellitus; glomerular filtration rate at baseline	Age (<45, 45-54, 55-59, ≥55); Sex (male, female); Diabetes (yes, no); Systolic blood pressure (<120, 120-129, 130-139, 140-149, 150-159, ≥160); eGFR (≥90, 75-89, 60-74)	Age (health survey), sex (health survey). Hypertension was defined as systolic blood pressure ≥ 140 mmHg or diastolic blood pressure ≥ 90 mmHg or use of oral antihypertensive medication. Diabetes mellitus was defined as a fasting glucose of ≥126 mg/dl or a positive history of diabetes. Serum creatinine (sCr) was measured by the enzymatic assay on the Vitros 350 analyzer (Ortho-Clinical Diagnostics, USA) using IDMS-Standard Reference Material (SRM) 967 as the standard. Estimate glomerular filtration rate (eGFR) was calculated according to two-level race variable Chronic Kidney Disease–Epidemiology Collaboration (CKDEPI) equation	NI
6	Saranburut, 2017 - Model 3 (derivation Clinical + Full	22	20	NI	Age; sex; systolic blood pressure; diabetes mellitus; glomerular filtration rate at baseline; uric acid; hemoglobin	Age (<45, 45-54, 55-59, ≥55); Sex (male, female); Diabetes (yes, no); Systolic blood pressure (<120, 120-129, 130-139, 140-149, 150-159, ≥160); eGFR (≥90, 75-89, 60-74); Uric acid (>6 for female or >7 for male, ≤6	Age (health survey), sex (health survey). Hypertension was defined as systolic blood pressure ≥ 140 mmHg or diastolic blood pressure ≥ 90 mmHg or use of oral	NI

For peer review only

For peer review only - http://bmjopen.bmj.com/site/about/guidelines.xhtml

	laboratory tests)					for female or ≤7 for male); Hemoglobin (<12 for female or <13 for male, ≥12 for female or ≥13 for male)	antihypertensive medication. Diabetes mellitus was defined as a fasting glucose of ≥126 mg/dl or a positive history of diabetes. Serum creatinine (sCr) was measured by the enzymatic assay on the Vitros 350 analyzer (Ortho-Clinical Diagnostics, USA) using IDMS-Standard Reference Material (SRM) 967 as the standard. Estimate glomerular filtration rate (eGFR) was calculated according to two-level race variable Chronic Kidney Disease–Epidemiology Collaboration (CKDEPI) equation. There is no information about uric acid and hemoglobin	
6	Saranburut, 2017 - Model 1 (validation Clinical only)	n/a	15	NI	Age; sex; systolic blood pressure; waist circumference; diabetes mellitus	Age (<45, 45-54, 55-59, ≥55); Sex (male, female); Waist circumference (≤80 for male or ≤90 for male, >80 for female or >90 for male); Diabetes (yes, no); Systolic blood pressure (<120, 120-129, 130-139, 140-149, 150-159, ≥160)	Age (health survey), sex (health survey). Hypertension was defined as systolic blood pressure ≥ 140 mmHg or diastolic blood pressure ≥ 90 mmHg or use of oral antihypertensive medication. Diabetes mellitus was defined as a fasting glucose of ≥126 mg/dl or a positive history of diabetes. Waist circumference was measured midway between the lowest ribs and the iliac crest.	n/a
6	Saranburut, 2017 - Model 2 (validation	n/a	16	NI	Age; sex; systolic blood pressure; diabetes mellitus; glomerular filtration rate at baseline	Age (<45, 45-54, 55-59, ≥55); Sex (male, female); Diabetes (yes, no); Systolic blood pressure (<120, 120-129, 130-139, 140-149, 150-159,	Age (health survey), sex (health survey). Hypertension was defined as systolic blood pressure ≥ 140 mmHg or	n/a

For peer review only

	Clinical + Limited laboratory tests)					≥160); eGFR (≥90, 75-89, 60-74)	diastolic blood pressure ≥ 90 mmHg or use of oral antihypertensive medication. Diabetes mellitus was defined as a fasting glucose of ≥126 mg/dl or a positive history of diabetes. Serum creatinine (sCr) was measured by the enzymatic assay on the Vitros 350 analyzer (Ortho-Clinical Diagnostics, USA) using IDMS-Standard Reference Material (SRM) 967 as the standard. Estimate glomerular filtration rate (eGFR) was calculated according to two-level race variable Chronic Kidney Disease–Epidemiology Collaboration (CKDEPI) equation	
7	Thakkinstian, 2011 (derivation)	37	10	NI	Age; history of kidney stones; diabetes mellitus; hypertension	Age (<40, 40-59, 60-69, ≥70); Hypertension (taking antihyper-tensive drug(s) or had systolic blood pressure ≥140 mmHg or diastolicblood pressure ≥90 mmHg); Diabetes (taking oral hypoglycaemicagents or fasting plasma glucose levels ≥126 mg/dL); History of kidney stone was measured by self-reporting kidney stone	Age (survey), diabetes (history of illness, relevant medicines used or laboratory tests/physical examinations), hypertension (history of illness, relevant medicines used or laboratory tests/physical examinations), and history of kidney stones (self-reported in survey).	NI
8	Wen, 2020 - Simple Risk Score (derivation)	NI	15	Time-varying	Waist circumference; systolic blood pressure; sex; education; diabetes	Waist circumference [<80/<75, 80-84.9/75-79.9, 85-89.9/80-84.9, 90-94.9/85-89.9, ≥95/≥90 (for male/female)]; systolic blood pressure (<120, 120-139, 140-159, >160); sex (male, female); education (illiterate, primary school and above); diabetes (no or yes)	During medical examinations, participants took two blood pressure measurements using a non-invasive automatic HEM-907 blood pressure monitor after 5 minutes of rest. Systolic blood pressure was identified as the	NI

For peer review only - http://bmjopen.bmj.com/site/about/guidelines.xhtml

For peer review only

							average values of two independent measurements; Diabetes was defined as: (1) FPG ≥7.0 mmol/L, or (2) self-reported diagnosis of diabetes, or (3) the use of antidiabetic medications; According to the number of years of education, they were divided into four groups (illiterate for 0 years, primary school for 1–6 years, junior high school for 7–9years, and senior high school for ≥10 years); Sex was self-reported; Information about waist circumference was no available	
8	Wen, 2020 - Best-fit Risk Score (derivation)	NI	19	Time-varying	Urinary Albumin-to-creatinine ratio; systolic blood pressure; C-reactive protein; triglycerides; sex; education; diabetes	Urinary Albumin-to-creatinine ratio (<5.0, 5.0-10.0, >10.0); systolic blood pressure (<120, 120-139, 140-159, >160); C-reactive protein (<1.0, 1-3, >3.0); triglycerides (<1.0, 1.0-1.7, >1.7); sex (male, female); education (illiterate, primary school and above); diabetes (no or yes)	Urinary albumin and creatinine were measured from fresh morning spot urine samples; During medical examinations, participants took two blood pressure measurements using a non-invasive automatic HEM-907 blood pressure monitor after 5 minutes of rest. Systolic blood pressure was identified as the average values of two independent measurements; Diabetes was defined as: (1) FPG ≥7.0 mmol/L, or (2) self-reported diagnosis of diabetes, or (3) the use of antidiabetic medications; According to the number of years of education, they were divided into four groups	NI

For peer review only - http://bmjopen.bmj.com/site/about/guidelines.xhtml

							(illiterate for 0 years, primary school for 1–6 years, junior high school for 7–9years, and senior high school for ≥10 years); Sex was self-reported; Information about waist circumference, C-reactive protein and triglycerides were no available	
9	Wu, 2016 (derivation)	NI	10	Baseline	Age, gender and body mass index (BMI) status.	Age (≤ 40, 41−50, 51−60, 61−70, ≥71), gender (male, female) and body mass index (BMI) status (normal, overweight: 23−24.9 kg/m2, obesity: ≥25 kg/m2).	Age (self-reported), gender (self-reported) and body mass index (BMI) status (calculated from participant's measured body weight and height).	NI
9	Wu, 2016 (validation)	n/a	10	Baseline	Age, gender and body mass index (BMI) status.	Age (≤ 40, 41 − 50, 51 − 60, 61 − 70, 71+), gender (male, female) and body mass index (BMI) status (normal, overweight: 23−24.9 kg/m2, obesity: ≥25 kg/m2).	Age (self-reported), gender (self-reported) and body mass index (BMI) status (calculated from participant's measured body weight and height).	n/a

For peer review only

S3.4 Table: Sample size and missing data

		Sample Size			Missing Data		
N°	Study	Baseline sample size	Number of outcome events	Total outcome events per candidate predictors	Missing data	Number of participants with missing data	Missing data per candidate predictors
1	Asgari, 2020 European Risk Assessment tool (6-years validation)	3270	722	n/a	Complete-case	2817	n/a
1	Asgari, 2020 European Risk Assessment tool (9-years validation)	3240	1359	n/a	Complete-case	2847	n/a
2	Bradshaw, 2019 - Model 1 (derivation)	8698	947	31,57	Complete-case	896	29,87
2	Bradshaw, 2019 - Model 2 (derivation)	8698	947	41,17	Complete-case	896	38,96
2	Bradshaw, 2019 - Model 3a (derivation)	8698	947	NI	Complete-case	896	NI
2	Bradshaw, 2019 - Model 3b (derivation)	8698	947	118,38	Complete-case	896	112,00
2	Bradshaw, 2019 - Model 3a (CARRS-I urban validation)	4065	NI	n/a	Complete-case	1300	n/a
2	Bradshaw, 2019 - Model 3a (UDAY rural validation)	4940	NI	n/a	Complete-case	1233	n/a
3	Carrillo-Larco, 2017 - CRONICAS-CKD (derivation complete)	2368	81	2,25	Complete-case	235	6,53
3	Carrillo-Larco, 2017 - CRONICAS-CKD (derivation lab-free)	2368	81	3,12	Complete-case	235	9,04
3	Carrillo-Larco, 2017 - CRONICAS-CKD (validation complete)	1459	79	n/a	Complete-case	79	n/a
3	Carrillo-Larco, 2017 - CRONICAS-CKD (validation lab-free)	1459	79	n/a	Complete-case	79	n/a
4	Mogueo, 2015 - Korean model (eGFR validation)	902	259	n/a	Complete-case	383	n/a
4	Mogueo, 2015 - Thai model (eGFR validation)	902	259	n/a	Complete-case	383	n/a
4	Mogueo, 2015 - Korean model (eGFR or proteinuria validation)	902	268	n/a	Complete-case	383	n/a
4	Mogueo, 2015 - Thai model (eGFR or proteinuria validation)	902	268	n/a	Complete-case	383	n/a
5	Saranburut, 2017 - Framingham Heart Study (MDRD validation)	2141	222	n/a	Complete-case	NI	n/a
5	Saranburut, 2017 - Framingham Heart Study (CKD-EPI validation)	2328	233	n/a	Complete-case	NI	n/a
6	Saranburut, 2017 - Model 1 (derivation Clinical only)	3186	271	18,07	Complete-case	NI	NI
6	Saranburut, 2017 - Model 1 BMI (derivation Clinical only)	3186	271	18,07	Complete-case	NI	NI
6	Saranburut, 2017 - Model 2 (derivation Clinical + Limited laboratory tests)	3186	271	16,94	Complete-case	NI	NI
6	Saranburut, 2017 - Model 3 (derivation Clinical + Full laboratory tests)	3186	271	12,32	Complete-case	NI	NI
6	Saranburut, 2017 - Model 1 (validation Clinical only)	1395	27	n/a	Complete-case	NI	NI

For peer review only

6	Saranburut, 2017 - Model 2 (validation Clinical + Limited laboratory tests)	1395	27	n/a	Complete-case	NI	NI
7	Thakkinstian, 2011 (derivation)	3459	626	16,92	NI	NI	NI
8	Wen, 2020 - Simple Risk Score (derivation)	3266	590	NI	Complete-case	992	NI
8	Wen, 2020 - Best-fit Risk Score (derivation)	3266	590	NI	Complete-case	992	NI
9	Wu, 2016 (derivation)	14374	294	NI	Complete-case	3135	NI
9	Wu, 2016 (validation)	4371	48	n/a	Complete-case	911	n/a

For peer review only

S3.5 Table: **Model development**

		Model Development					
N°	Study	Regression method	Were the model assumptions verified?	Predictors selection	If the prediction model was a replication, which was the original model?	If there were pre-selection, describe the method	Was a shrinkage method used?
1	Asgari, 2020 European Risk Assessment tool (6-years validation)	n/a	n/a	n/a	n/a	n/a	n/a
1	Asgari, 2020 European Risk Assessment tool (9-years validation)	n/a	n/a	n/a	n/a	n/a	n/a
2	Bradshaw, 2019 - Model 1 (derivation)	Logistic	NI	Pre-selection	n/a	Step-down selection procedure based on the Akaike information criterion to select the final predictors	No
2	Bradshaw, 2019 - Model 2 (derivation)	Logistic	NI	Pre-selection	n/a	Step-down selection procedure based on the Akaike information criterion to select the final predictors	No
2	Bradshaw, 2019 - Model 3a (derivation)	Logistic	NI	Pre-selection	n/a	Step-down selection procedure based on the Akaike information criterion to select the final predictors	No
2	Bradshaw, 2019 - Model 3b (derivation)	Logistic	NI	Pre-selection	n/a	Step-down selection procedure based on the Akaike information criterion to select the final predictors	No
2	Bradshaw, 2019 - Model 3a (CARRS-I urban validation)	n/a	n/a	n/a	n/a	n/a	n/a
2	Bradshaw, 2019 - Model 3a (UDAY rural validation)	n/a	n/a	n/a	n/a	n/a	n/a
3	Carrillo-Larco, 2017 - CRONICAS-CKD (derivation complete)	Logistic	NI	Pre-selection	n/a	Stepwise backward elimination method	No

For peer review only - http://bmjopen.bmj.com/site/about/guidelines.xhtml

3	Carrillo-Larco, 2017 - CRONICAS-CKD (derivation lab-free)	Logistic	NI	Pre-selection	n/a	Stepwise backward elimination method	No
3	Carrillo-Larco, 2017 - CRONICAS-CKD (validation complete)	n/a	n/a	n/a	n/a	n/a	n/a
3	Carrillo-Larco, 2017 - CRONICAS-CKD (validation lab-free)	n/a	n/a	n/a	n/a	n/a	n/a
4	Mogueo, 2015 - Korean model (eGFR validation)	n/a	n/a	n/a	n/a	n/a	n/a
4	Mogueo, 2015 - Thai model (eGFR validation)	n/a	n/a	n/a	n/a	n/a	n/a
4	Mogueo, 2015 - Korean model (eGFR or proteinuria validation)	n/a	n/a	n/a	n/a	n/a	n/a
4	Mogueo, 2015 - Thai model (eGFR or proteinuria validation)	n/a	n/a	n/a	n/a	n/a	n/a
5	Saranburut, 2017 - Framingham Heart Study (MDRD validation)	n/a	n/a	n/a	n/a	n/a	n/a
5	Saranburut, 2017 - Framingham Heart Study (CKD-EPI validation)	n/a	n/a	n/a	n/a	n/a	n/a
6	Saranburut, 2017 - Model 1 (derivation Clinical only)	Logistic	NI	Pre-selection	n/a	Variables were sequentially added in a pre-specified order and incorporated using a p< 0.05 threshold for entry and retention in the final model	No
6	Saranburut, 2017 - Model 1 BMI (derivation Clinical only)	Logistic	NI	Pre-selection	n/a	Variables were sequentially added in a pre-specified order and incorporated using a p< 0.05 threshold for entry and retention in the final model	No
6	Saranburut, 2017 - Model 2 (derivation Clinical + Limited laboratory tests)	Logistic	NI	Pre-selection	n/a	Variables were sequentially added in a pre-specified order and incorporated using a p< 0.05 threshold for entry and retention in the final model	No
6	Saranburut, 2017 - Model 3 (derivation Clinical + Full laboratory tests)	Logistic	NI	Pre-selection	n/a	Variables were sequentially added in a pre-specified order and incorporated using a p< 0.05 threshold for entry and retention in the final model	No

For peer review only

For peer review only - http://bmjopen.bmj.com/site/about/guidelines.xhtml

6	Saranburut, 2017 - Model 1 (validation Clinical only)	n/a	n/a	n/a	n/a	n/a	n/a
6	Saranburut, 2017 - Model 2 (validation Clinical + Limited laboratory tests)	n/a	n/a	n/a	n/a	n/a	n/a
7	Thakkinstian, 2011 (derivation)	Logistic	NI	Pre-selection	n/a	Factors with p values < 0.15 in a univariate analysis were considered to be simultaneously included in the multivariate logistic equation. Model selection was performed using F-tests, and thus only significant variables were kept in the final model. C statistic of models with and without a particular variable were then compared; if dropping that variable did not significantly reduce the explanation of the CKD, that variable was omitted in the final parsimonious model.	No
8	Wen, 2020 - Simple Risk Score (derivation)	Logistic	NI	Pre-selection	n/a	Risk factors were investigated by forward stepwise logistic regression and only statiscally significant (a two-sided P value <0.05) risk factors were retained.	No
8	Wen, 2020 - Best-fit Risk Score (derivation)	Logistic	NI	Pre-selection	n/a	Risk factors were investigated by forward stepwise logistic regression and only statiscally significant (a two-sided P value <0.05) risk factors were retained.	No
9	Wu, 2016 (derivation)	Logistic	NI	Pre-selection	n/a	Stepwise logistic regression model. Variables with a p value less than 0.1 were kept in the final model.	No
9	Wu, 2016 (validation)	n/a	n/a	n/a	n/a	n/a	n/a

For peer review only - http://bmjopen.bmj.com/site/about/guidelines.xhtml

n/a: not applicable; NI: no information

S3.6 Table: Model performance

		Model Performance				
N°	Study	Calibration	Discrimination (%)	Classification measures	Cut-off point	For replication studies, was the cut-off the same?
1	Asgari, 2020 European Risk Assessment tool (6-years validation)	Hosmer-Lemeshow X2 test (for intercept adjusted model): 13.53 with a p-value 0.09 (for male) and 10.1 with a p-value 0.26 (for women)	AUC (95% CI) for final intercept adjusted model = Male: 0.76 (0.72-0.79) and Female: 0.71 (0.69-0.73)	Men: Sensitivity = 72.7%, Specificity = 67.6%. Women: Sensitivity = 66.8%, Specificity = 65.6%.	Men: 25. Women: 19	No
1	Asgari, 2020 European Risk Assessment tool (9-years validation)	Hosmer-Lemeshow X2 test (for intercept adjusted model): 12.54 with a p-value 0.13 (for male) and 8.19 with a p-value 0.41 (for women)	AUC (95% CI) for final intercept adjusted model = Male: 0.71 (0.67-0.74) and Female: 0.70 (0.68-0.73)	Men: Sensitivity = 64.5%, Specificity = 69.5%. Women: Sensitivity = 56.9%, Specificity = 76.6%	Men: 25. Women: 23	No
2	Bradshaw, 2019 - Model 1 (derivation)	Calibration slope: 0.96	C-statistic (95% CI) = 0.79 (0.78-0.81)	Sensitivity = 72%, Specificity = 72%, PPV = 24%, NPV = 96%	0.09	n/a
2	Bradshaw, 2019 - Model 2 (derivation)	Calibration slope: 0.98	C-statistic (95% CI) = 0.73 (0.72-0.75)	Sensitivity = 68%, Specificity = 67%, PPV = 20%, NPV = 95%	0.09	n/a
2	Bradshaw, 2019 - Model 3a (derivation)	Calibration slope: 0.98	C-statistic (95% CI) = 0.77 (0.75-0.79)	Sensitivity = 71%, Specificity = 70%, PPV = 22%, NPV = 95%	0.09	n/a
2	Bradshaw, 2019 - Model 3b (derivation)	Calibration slope: 0.99	C-statistic (95% CI) = 0.77 (0.76-0.79)	Sensitivity = 71%, Specificity = 70%, PPV = 22%, NPV = 95%	0.09	n/a

For peer review only - http://bmjopen.bmj.com/site/about/guidelines.xhtml

2	Bradshaw, 2019 - Model 3a (CARRS-I urban validation)	NI	C-statistic (95% CI) = 0.74 (0.73-0.74)	NI	0.09	Yes
2	Bradshaw, 2019 - Model 3a (UDAY rural validation)	NI	C-statistic (95% CI) = 0.70 (0.69-0.71)	NI	0.09	Yes
3	Carrillo-Larco, 2017 - CRONICAS-CKD (derivation complete)	Hosmer-Lemeshow X2 test: 4.13 with a p-value of 0.53 (for final multivariable model).	AUC = 76.2%	Sensibility = 82.5%, Specificity = 70.0%, PPV = 8.8%, NPV = 99.1%, LHR+ = 2.8, LHR- = 0.3	2	n/a
3	Carrillo-Larco, 2017 - CRONICAS-CKD (derivation lab-free)	Hosmer-Lemeshow X2 test: 4.13 with a p-value of 0.53 (for final multivariable model).	AUC = 76%	Sensibility = 80.0%, Specificity = 72.0%, PPV = 9.1%, NPV = 99.0%, LHR+ = 2.9, LHR- = 0.3	2	n/a
3	Carrillo-Larco, 2017 - CRONICAS-CKD (validation complete)	NI	AUC = 70.0%.	Sensitivity = 70.5%, Specificity = 69.1%, PPV = 11.4%, NPV = 97.6%, LHR+ = 2.3, LHR- = 0.4	2	Yes
3	Carrillo-Larco, 2017 - CRONICAS-CKD (validation lab-free)	NI	AUC = 70.0%.	Sensitivity = 70.5%, Specificity = 69.7%, PPV = 11.6%, NPV = 97.7%, LHR+ = 2.3, LHR- = 0.4	2	Yes
4	Mogueo, 2015 - Korean model (eGFR validation)	Expected/Observed rate (95%) = 0.76 (0.67-0.86); Brier score = 0.164; Yates slope = 0.208	C-statistic (95% CI) = 0.797 (0.765-0.829)	Sensitivity = 82%, Specificity = 67%	0.30	NI
4	Mogueo, 2015 - Thai model (eGFR validation)	Expected/Observed rate (95%) = 0.98 (0.87-1.10); Brier score = 0.165; Yates slope = 0.200	C-statistic (95% CI) = 0.760 (0.726-0.793)	Sensitivity = 73%, Specificity = 72%	0.31	NI
4	Mogueo, 2015 - Korean model (eGFR or proteinuria validation)	Expected/Observed rate (95%) = 0.76 (0.67-0.85); Brier score = 0.161; Yates slope = 0.225	C-statistic (95% CI) = 0.811 (0.780-0.842)	Sensitivity = 84%, Specificity = 68%	0.31	NI

For peer review only - http://bmjopen.bmj.com/site/about/guidelines.xhtml

4	Mogueo, 2015 - Thai model (eGFR or proteinuria validation)	Expected/Observed rate (95%) = 0.97 (0.86-1.09); Brier score = 0.164; Yates slope = 0.211	C-statistic (95% CI) = 0.772 (0.739-0.805)	Sensitivity = 74%, Specificity = 73%	0.32	NI
5	Saranburut, 2017 - Framingham Heart Study (MDRD validation)	Hosmer-Lemeshow X2 test: 30.2 (p<0.001)	AUC (95% CI) = 0.69 (0.66-0.73)	NI	NI	NI
5	Saranburut, 2017 - Framingham Heart Study (CKD-EPI validation)	Hosmer-Lemeshow X2 test: 256.5 (p<0.001)	AUC (95% CI) = 0.63 (0.57-0.65)	NI	NI	NI
6	Saranburut, 2017 - Model 1 (derivation Clinical only)	Hosmer-Lemeshow X2 test: 9.02 (p=0.34)	AUC (95% CI) = 0.72 (0.69-0.75)	NI	NI	n/a
6	Saranburut, 2017 - Model 1 BMI (derivation Clinical only)	Hosmer-Lemeshow X2 test: 8.87 (p=0.35)	AUC (95% CI) = 0.72 (0.69-0.75)	NI	NI	n/a
6	Saranburut, 2017 - Model 2 (derivation Clinical + Limited laboratory tests)	Hosmer-Lemeshow X2 test: 10.87 (p=0.21)	AUC (95% CI) = 0.79 (0.76-0.82)	NI	NI	n/a
6	Saranburut, 2017 - Model 3 (derivation Clinical + Full laboratory tests)	Hosmer-Lemeshow X2 test: 8.28 (p=0.41)	AUC (95% CI) = 0.80 (0.77-0.82)	NI	NI	n/a
6	Saranburut, 2017 - Model 1 (validation Clinical only)	Hosmer-Lemeshow X2 test: 4.31 (p=0.229)	AUC (95% CI) = 0.66 (0.55-0.78)	NI	NI	NI
6	Saranburut, 2017 - Model 2 (validation Clinical + Limited laboratory tests)	Hosmer-Lemeshow X2 test: 2.29 (p=0.514)	AUC (95% CI) = 0.88 (0.80-0.95)	NI	NI	NI
7	Thakkinstian, 2011 (derivation)	Calibration was assessed by subtracting the two Somer's D correlation coefficients: 0.045 (95% CI: 0.034-0.057)	C-statistic of internal validation = 0.741	Sensitivity = 76%, Specificity = 69%	5	n/a

For peer review only - http://bmjopen.bmj.com/site/about/guidelines.xhtml

8	Wen, 2020 - Simple Risk Score (derivation)	Hosmer-Lemeshow X2 test: 4.89 (p=0.769)	AUC (95% CI) = 0.717 (0.689-0.744)	Sensitivity = 70.49%, Specificity = 65.14%, PPV = 29.8%, NPV = 91.3%, LHR+ = 2.02, LHR- = 0.45	14	n/a
8	Wen, 2020 - Best-fit Risk Score (derivation)	Hosmer-Lemeshow X2 test: 2.52 (p=0.961)	AUC (95% CI) = 0.721 (0.693-0.748)	Sensitivity = 56.83%, Specificity = 76.61%, PPV = 33.8%, NPV = 89.4%, LHR+ = 2.43, LHR- = 0.56	24	n/a
9	Wu, 2016 (derivation)	Internal validation dataset: Hosmer-Lemeshow X2 test P=0.798	AUC (95% CI) of internal validation = 0.894 (0.861-0.926)	Sensitivity = 0.820, Specificity = 0.863	36	n/a
9	Wu, 2016 (validation)	Hosmer-Lemeshow X2 test P=397	AUC = 0.880 (95%CI: 0.829-0.931)	NI	NI	NI

AUC, area under the curve; CI, confident interval; NI, no information.

For peer review only

S3.7 Table: Results

		Results			
N°	Study	Was a simplified model presented?	Were the coefficients of the regression model presented?	Was the baseline risk presented?	Were there alternative results presentation?
1	Asgari, 2020 European Risk Assessment tool (6-years validation)	No	No	Yes	No
1	Asgari, 2020 European Risk Assessment tool (9-years validation)	No	No	Yes	No
2	Bradshaw, 2019 - Model 1 (derivation)	Yes	No	No	No
2	Bradshaw, 2019 - Model 2 (derivation)	Yes	No	No	No
2	Bradshaw, 2019 - Model 3a (derivation)	No	No	No	No
2	Bradshaw, 2019 - Model 3b (derivation)	Yes	No	No	No
2	Bradshaw, 2019 - Model 3a (CARRS-I urban validation)	No	No	No	No
2	Bradshaw, 2019 - Model 3a (UDAY rural validation)	No	No	No	No
3	Carrillo-Larco, 2017 - CRONICAS-CKD (derivation complete)	Yes	Yes	No	No
3	Carrillo-Larco, 2017 - CRONICAS-CKD (derivation lab-free)	No	Yes	No	No
3	Carrillo-Larco, 2017 - CRONICAS-CKD (validation complete)	Yes	No	No	No
3	Carrillo-Larco, 2017 - CRONICAS-CKD (validation lab-free)	No	No	No	No
4	Mogueo, 2015 - Korean model (eGFR validation)	No	No	No	No
4	Mogueo, 2015 - Thai model (eGFR validation)	No	No	No	No
4	Mogueo, 2015 - Korean model (eGFR or proteinuria validation)	No	No	No	No
4	Mogueo, 2015 - Thai model (eGFR or proteinuria validation)	No	No	No	No
5	Saranburut, 2017 - Framingham Heart	No	Yes	No	No

For peer review only - http://bmjopen.bmj.com/site/about/guidelines.xhtml

	Study (MDRD validation)				
5	Saranburut, 2017 - Framingham Heart Study (CKD-EPI validation)	No	Yes	No	No
6	Saranburut, 2017 - Model 1 (derivation Clinical only)	No	Yes	No	Yes
6	Saranburut, 2017 - Model 1 BMI (derivation Clinical only)	No	No	No	Yes
6	Saranburut, 2017 - Model 2 (derivation Clinical + Limited laboratory tests)	Yes	Yes	No	Yes
6	Saranburut, 2017 - Model 3 (derivation Clinical + Full laboratory tests)	Yes	Yes	No	No
6	Saranburut, 2017 - Model 1 (validation Clinical only)	No	No	No	Yes
6	Saranburut, 2017 - Model 2 (validation Clinical + Limited laboratory tests)	Yes	No	No	Yes
7	Thakkinstian, 2011 (derivation)	No	Yes	No	Yes
8	Wen, 2020 - Simple Risk Score (derivation)	No	Yes	Yes	Yes
8	Wen, 2020 - Best-fit Risk Score (derivation)	No	Yes	Yes	Yes
9	Wu, 2016 (derivation)	No	Yes	No	Yes
9	Wu, 2016 (validation)	No	Yes	No	Yes

For peer review only - http://bmjopen.bmj.com/site/about/guidelines.xhtml

S3.8 Table: Discussion

		Discussion		
N°	Study	Interpretation of the results	Comparison with other studies in LAC	Generalizability
1	Asgari, 2020 European Risk Assessment tool (6-years validation)	Exploratory	No	Non-generalizability
1	Asgari, 2020 European Risk Assessment tool (9-years validation)	Exploratory	No	Non-generalizability
2	Bradshaw, 2019 - Model 1 (derivation)	NI	No	NI
2	Bradshaw, 2019 - Model 2 (derivation)	NI	No	NI
2	Bradshaw, 2019 - Model 3a (derivation)	Confirmatory	Yes	Non-generalizability
2	Bradshaw, 2019 - Model 3b (derivation)	NI	No	NI
2	Bradshaw, 2019 - Model 3a (CARRS-I urban validation)	Confirmatory	Yes	Non-generalizability
2	Bradshaw, 2019 - Model 3a (UDAY rural validation)	Confirmatory	Yes	Non-generalizability
3	Carrillo-Larco, 2017 - CRONICAS-CKD (derivation complete)	Exploratory	Yes	Generalizable
3	Carrillo-Larco, 2017 - CRONICAS-CKD (derivation lab-free)	Exploratory	Yes	Generalizable
3	Carrillo-Larco, 2017 - CRONICAS-CKD (validation complete)	Exploratory	Yes	Generalizable
3	Carrillo-Larco, 2017 - CRONICAS-CKD (validation lab-free)	Exploratory	Yes	Generalizable
4	Mogueo, 2015 - Korean model (eGFR validation)	Exploratory	Yes	Non-generalizability
4	Mogueo, 2015 - Thai model (eGFR validation)	Exploratory	Yes	Non-generalizability
4	Mogueo, 2015 - Korean model (eGFR or proteinuria validation)	Exploratory	Yes	Non-generalizability
4	Mogueo, 2015 - Thai model (eGFR or proteinuria validation)	Exploratory	Yes	Non-generalizability
5	Saranburut, 2017 - Framingham Heart Study (MDRD validation)	Exploratory	No	Non-generalizability
5	Saranburut, 2017 - Framingham Heart Study (CKD-EPI validation)	Exploratory	No	Non-generalizability
6	Saranburut, 2017 - Model 1 (derivation Clinical only)	Exploratory	No	Non-generalizability
6	Saranburut, 2017 - Model 1 BMI (derivation Clinical only)	Exploratory	No	Non-generalizability
6	Saranburut, 2017 - Model 2 (derivation Clinical + Limited laboratory tests)	Exploratory	No	Non-generalizability
6	Saranburut, 2017 - Model 3 (derivation Clinical + Full laboratory tests)	Exploratory	No	Non-generalizability
6	Saranburut, 2017 - Model 1 (validation Clinical only)	Exploratory	No	Non-generalizability
6	Saranburut, 2017 - Model 2 (validation Clinical + Limited laboratory tests)	Exploratory	No	Non-generalizability
7	Thakkinstian, 2011 (derivation)	Confirmatory	No	Non-generalizability

For peer review only - http://bmjopen.bmj.com/site/about/guidelines.xhtml

8	Wen, 2020 - Simple Risk Score (derivation)	Confirmatory	Yes	Non-generalizability
8	Wen, 2020 - Best-fit Risk Score (derivation)	Exploratory	Yes	Non-generalizability
9	Wu, 2016 (derivation)	Exploratory	No	Non-generalizability
9	Wu, 2016 (validation)	Exploratory	No	Non-generalizability

For peer review only

For peer review only - http://bmjopen.bmj.com/site/about/guidelines.xhtml

S4 Table: PROBAST
S4.1 Table: Risk of Bias (RoB)

Study	Participants		Predictors		
	Were appropriate data sources used, e.g., cohort, RCT, or nested case–control study data?	Were all inclusions and exclusions of participants appropriate?	Were predictors defined and assessed in a similar way for all participants?	Were predictor assessments made without knowledge of outcome data?	Are all predictors available at the time the model is intended to be used?
Asgari, 2020 European Risk Assessment tool (6-years validation)	Y	Y	Y	Y	Y
Asgari, 2020 European Risk Assessment tool (9-years validation)	Y	Y	Y	Y	Y
Bradshaw, 2019 - Model 1 (derivation)	Y	Y	Y	Y	PY
Bradshaw, 2019 - Model 2 (derivation)	Y	Y	Y	Y	Y
Bradshaw, 2019 - Model 3a (derivation)	Y	Y	Y	Y	PY
Bradshaw, 2019 - Model 3b (derivation)	Y	Y	Y	Y	PY
Bradshaw, 2019 - Model 3a (CARRS-I urban validation)	Y	Y	Y	Y	PY
Bradshaw, 2019 - Model 3a (UDAY rural validation)	Y	Y	Y	Y	PY
Carrillo-Larco, 2017 - CRONICAS-CKD (derivation complete)	Y	Y	Y	Y	PY
Carrillo-Larco, 2017 - CRONICAS-CKD (derivation lab-free)	Y	Y	Y	Y	Y
Carrillo-Larco, 2017 - CRONICAS-CKD (validation complete)	Y	Y	Y	Y	PY
Carrillo-Larco, 2017 - CRONICAS-CKD (validation lab-free)	Y	Y	Y	Y	Y
Mogueo, 2015 - Korean model (eGFR validation)	Y	Y	Y	Y	PY
Mogueo, 2015 - Thai model (eGFR validation)	Y	Y	Y	Y	Y
Mogueo, 2015 - Korean model (eGFR or proteinuria validation)	Y	Y	Y	Y	PY
Mogueo, 2015 - Thai model (eGFR or proteinuria validation)	Y	Y	Y	Y	Y

For peer review only - http://bmjopen.bmj.com/site/about/guidelines.xhtml

Saranburut, 2017 - Framingham Heart Study (MDRD validation)	Y	Y	Y	Y	PY
Saranburut, 2017 - Framingham Heart Study (CKD-EPI validation)	Y	Y	Y	Y	PY
Saranburut, 2017 - Model 1 (derivation Clinical only)	Y	Y	Y	Y	Y
Saranburut, 2017 - Model 1 BMI (derivation Clinical only)	Y	Y	Y	Y	Y
Saranburut, 2017 - Model 2 (derivation Clinical + Limited laboratory tests)	Y	Y	Y	Y	PY
Saranburut, 2017 - Model 3 (derivation Clinical + Full laboratory tests)	Y	Y	Y	Y	PY
Saranburut, 2017 - Model 1 (validation Clinical only)	Y	Y	Y	Y	Y
Saranburut, 2017 - Model 2 (validation Clinical + Limited laboratory tests)	Y	Y	Y	Y	PY
Thakkinstian, 2011 (derivation)	Y	Y	Y	Y	Y
Wen, 2020 - Simple Risk Score (derivation)	Y	Y	Y	Y	Y
Wen, 2020 - Best-fit Risk Score (derivation)	Y	Y	Y	Y	Y
Wu, 2016 (derivation)	Y	Y	Y	Y	Y
Wu, 2016 (validation)	Y	Y	Y	Y	Y

Answer options: Y (yes), PY (probably yes), N (no), PN (probably no), NI (no information), n/a (not applicable).

For peer review only

For peer review only - http://bmjopen.bmj.com/site/about/guidelines.xhtml

Study	Outcome					
	Was the outcome determined appropriately?	Was a prespecified or standard outcome definition used?	Were predictors excluded from the outcome definition?	Was the outcome defined and determined in a similar way for all participants?	Was the outcome determined without knowledge of predictor information?	Was the time interval between predictor assessment and outcome determination appropriate?
Asgari, 2020 European Risk Assessment tool (6-years validation)	Y	Y	Y	Y	NI	Y
Asgari, 2020 European Risk Assessment tool (9-years validation)	Y	Y	Y	Y	NI	Y
Bradshaw, 2019 - Model 1 (derivation)	Y	Y	Y	Y	NI	PY
Bradshaw, 2019 - Model 2 (derivation)	Y	Y	Y	Y	NI	Y
Bradshaw, 2019 - Model 3a (derivation)	NI	Y	Y	Y	NI	PY
Bradshaw, 2019 - Model 3b (derivation)	Y	Y	Y	Y	NI	PY
Bradshaw, 2019 - Model 3a (CARRS-I urban validation)	Y	Y	Y	Y	NI	PY
Bradshaw, 2019 - Model 3a (UDAY rural validation)	Y	Y	Y	Y	NI	PY
Carrillo-Larco, 2017 - CRONICAS-CKD (derivation complete)	Y	Y	Y	Y	PY	PY
Carrillo-Larco, 2017 - CRONICAS-CKD (derivation lab-free)	Y	Y	Y	Y	PY	Y
Carrillo-Larco, 2017 - CRONICAS-CKD (validation complete)	Y	Y	Y	Y	PY	PY

For peer review only - http://bmjopen.bmj.com/site/about/guidelines.xhtml

Carrillo-Larco, 2017 - CRONICAS-CKD (validation lab-free)	Y	Y	Y	Y	PY	Y
Mogueo, 2015 - Korean model (eGFR validation)	Y	Y	Y	Y	NI	PY
Mogueo, 2015 - Thai model (eGFR validation)	Y	Y	Y	Y	NI	Y
Mogueo, 2015 - Korean model (eGFR or proteinuria validation)	Y	Y	Y	Y	NI	PY
Mogueo, 2015 - Thai model (eGFR or proteinuria validation)	Y	Y	Y	Y	NI	Y
Saranburut, 2017 - Framingham Heart Study (MDRD validation)	Y	Y	Y	Y	NI	PY
Saranburut, 2017 - Framingham Heart Study (CKD-EPI validation)	Y	Y	Y	Y	NI	PY
Saranburut, 2017 - Model 1 (derivation Clinical only)	Y	Y	Y	Y	NI	Y
Saranburut, 2017 - Model 1 BMI (derivation Clinical only)	Y	Y	Y	Y	NI	Y
Saranburut, 2017 - Model 2 (derivation Clinical + Limited laboratory tests)	Y	Y	Y	Y	NI	PY
Saranburut, 2017 - Model 3 (derivation Clinical + Full laboratory tests)	Y	Y	Y	Y	NI	PY
Saranburut, 2017 - Model 1 (validation Clinical only)	Y	Y	Y	Y	NI	Y
Saranburut, 2017 - Model 2 (validation Clinical + Limited laboratory tests)	Y	Y	Y	Y	NI	PY
Thakkinstian, 2011 (derivation)	Y	Y	Y	Y	NI	Y
Wen, 2020 - Simple Risk Score (derivation)	Y	Y	Y	Y	NI	Y

For peer review only - http://bmjopen.bmj.com/site/about/guidelines.xhtml

Wen, 2020 - Best-fit Risk Score (derivation)	Y	Y	Y	Y	NI	Y
Wu, 2016 (derivation)	Y	Y	Y	Y	NI	Y
Wu, 2016 (validation)	Y	Y	Y	Y	NI	Y

Answer options: Y (yes), PY (probably yes), N (no), PN (probably no), NI (no information), n/a (not applicable).

For peer review only

For peer review only - http://bmjopen.bmj.com/site/about/guidelines.xhtml

Study	Analysis								
	Were there a reasonable number of participants with the outcome?	Were continuous and categorical predictors handled appropriately?	Were all enrolled participants included in the analysis?	Were participants with missing data handled appropriately?	Was selection of predictors based on univariable analysis avoided? [development studies only]	Were complexities in the data (e.g., censoring, competing risks, sampling of control participants) accounted for appropriately?	Were relevant model performance measures evaluated appropriately?	Were model overfitting and optimism in model performance accounted for? [development studies only]	Do predictors and their assigned weights in the final model correspond to the results from the reported multivariable analysis? [development studies only]
Asgari, 2020 European Risk Assessment tool (6-years validation)	Y	Y	N	N	n/a	NI	N	n/a	n/a
Asgari, 2020 European Risk Assessment tool (9-years validation)	Y	Y	N	N	n/a	NI	N	n/a	n/a
Bradshaw, 2019 - Model 1 (derivation)	Y	N	N	N	N	NI	Y	Y	NI
Bradshaw, 2019 - Model 2 (derivation)	Y	N	N	N	N	NI	Y	Y	NI
Bradshaw, 2019 - Model 3a (derivation)	NI	NI	N	N	N	NI	Y	Y	NI
Bradshaw, 2019 - Model 3b (derivation)	Y	N	N	N	N	NI	Y	Y	NI
Bradshaw, 2019 - Model 3a (CARRS-I urban validation)	NI	Y	N	N	n/a	NI	NI	n/a	n/a

For peer review only - http://bmjopen.bmj.com/site/about/guidelines.xhtml

Bradshaw, 2019 - Model 3a (UDAY rural validation)	NI	Y	N	N	n/a	NI	NI	n/a	n/a
Carrillo-Larco, 2017 - CRONICAS-CKD (derivation complete)	N	N	N	N	N	NI	N	Y	Y
Carrillo-Larco, 2017 - CRONICAS-CKD (derivation lab-free)	N	N	N	N	N	NI	N	Y	Y
Carrillo-Larco, 2017 - CRONICAS-CKD (validation complete)	N	Y	N	N	n/a	NI	N	n/a	n/a
Carrillo-Larco, 2017 - CRONICAS-CKD (validation lab-free)	N	Y	N	N	n/a	NI	N	n/a	n/a
Mogueo, 2015 - Korean model (eGFR validation)	Y	Y	N	N	n/a	NI	PY	n/a	n/a
Mogueo, 2015 - Thai model (eGFR validation)	Y	Y	N	N	n/a	NI	PY	n/a	n/a
Mogueo, 2015 - Korean model (eGFR or proteinuria validation)	Y	Y	N	N	n/a	NI	PY	n/a	n/a
Mogueo, 2015 - Thai model (eGFR or proteinuria validation)	Y	Y	N	N	n/a	NI	PY	n/a	n/a
Saranburut, 2017 - Framingham Heart Study (MDRD validation)	Y	Y	N	N	n/a	NI	N	n/a	n/a
Saranburut, 2017 - Framingham Heart Study (CKD-EPI validation)	Y	Y	N	N	n/a	NI	N	n/a	n/a
Saranburut, 2017 - Model 1 (derivation Clinical only)	PY	N	N	N	N	NI	N	Y	Y
Saranburut, 2017 - Model 1 BMI (derivation Clinical only)	PY	N	N	N	N	NI	N	Y	NI

For peer review only - http://bmjopen.bmj.com/site/about/guidelines.xhtml

Saranburut, 2017 - Model 2 (derivation Clinical + Limited laboratory tests)	PY	N	N	N	N	NI	N	Y	Y
Saranburut, 2017 - Model 3 (derivation Clinical + Full laboratory tests)	PN	N	N	N	N	NI	N	Y	Y
Saranburut, 2017 - Model 1 (validation Clinical only)	N	Y	N	N	n/a	NI	N	n/a	n/a
Saranburut, 2017 - Model 2 (validation Clinical + Limited laboratory tests)	N	Y	N	N	n/a	NI	N	n/a	n/a
Thakkinstian, 2011 (derivation)	PY	N	NI	NI	N	NI	N	Y	Y
Wen, 2020 - Simple Risk Score (derivation)	NI	N	N	N	N	NI	N	N	Y
Wen, 2020 - Best-fit Risk Score (derivation)	NI	N	N	N	N	NI	N	N	Y
Wu, 2016 (derivation)	NI	N	N	N	N	NI	N	N	Y
Wu, 2016 (validation)	N	Y	N	N	n/a	NI	N	n/a	Y

Answer options: Y (yes), PY (probably yes), N (no), PN (probably no), NI (no information), n/a (not applicable).

For peer review only - http://bmjopen.bmj.com/site/about/guidelines.xhtml

S4.2 Table: Applicability

N°	Study	Participants	Predictors	Outcome
1	Asgari, 2020 European Risk Assessment tool (6-years validation)	Low	Low	Low
1	Asgari, 2020 European Risk Assessment tool (9-years validation)	Low	Low	Low
2	Bradshaw, 2019 - Model 1 (derivation)	Low	Low	Low
2	Bradshaw, 2019 - Model 2 (derivation)	Low	Low	Low
2	Bradshaw, 2019 - Model 3a (derivation)	Low	Low	Low
2	Bradshaw, 2019 - Model 3b (derivation)	Low	Low	Low
2	Bradshaw, 2019 - Model 3a (CARRS-I urban validation)	Low	Low	Low
2	Bradshaw, 2019 - Model 3a (UDAY rural validation)	Low	Low	Low
3	Carrillo-Larco, 2017 - CRONICAS-CKD (derivation complete)	Low	Low	Low
3	Carrillo-Larco, 2017 - CRONICAS-CKD (derivation lab-free)	Low	Low	Low
3	Carrillo-Larco, 2017 - CRONICAS-CKD (validation complete)	Low	Low	Low
3	Carrillo-Larco, 2017 - CRONICAS-CKD (validation lab-free)	Low	Low	Low
4	Mogueo, 2015 - Korean model (eGFR validation)	Low	Low	Low
4	Mogueo, 2015 - Thai model (eGFR validation)	Low	Low	Low
4	Mogueo, 2015 - Korean model (eGFR or proteinuria validation)	Low	Low	Low
4	Mogueo, 2015 - Thai model (eGFR or proteinuria validation)	Low	Low	Low
5	Saranburut, 2017 - Framingham Heart Study (MDRD validation)	Low	Low	Low
5	Saranburut, 2017 - Framingham Heart Study (CKD-EPI validation)	Low	Low	Low
6	Saranburut, 2017 - Model 1 (derivation Clinical only)	Low	Low	Low
6	Saranburut, 2017 - Model 1 BMI (derivation Clinical only)	Low	Low	Low
6	Saranburut, 2017 - Model 2 (derivation Clinical + Limited laboratory tests)	Low	Low	Low
6	Saranburut, 2017 - Model 3 (derivation Clinical + Full laboratory tests)	Low	Low	Low
6	Saranburut, 2017 - Model 1 (validation Clinical only)	Low	Low	Low
6	Saranburut, 2017 - Model 2 (validation Clinical + Limited laboratory tests)	Low	Low	Low
7	Thakkinstian, 2011 (derivation)	Low	Low	Low
8	Wen, 2020 - Simple Risk Score (derivation)	Low	Low	Low
8	Wen, 2020 - Best-fit Risk Score (derivation)	Low	Low	Low
9	Wu, 2016 (derivation)	Low	Low	Low

For peer review only - http://bmjopen.bmj.com/site/about/guidelines.xhtml

9	Wu, 2016 (validation)	Low	Low	Low

Answer options: Low (low concern for applicability), Hig (High concern for applicability) and Unclear (Unclear concern for applicability)

For peer review only

For peer review only - http://bmjopen.bmj.com/site/about/guidelines.xhtml

S1 Figure: Countries where studies were conducted.

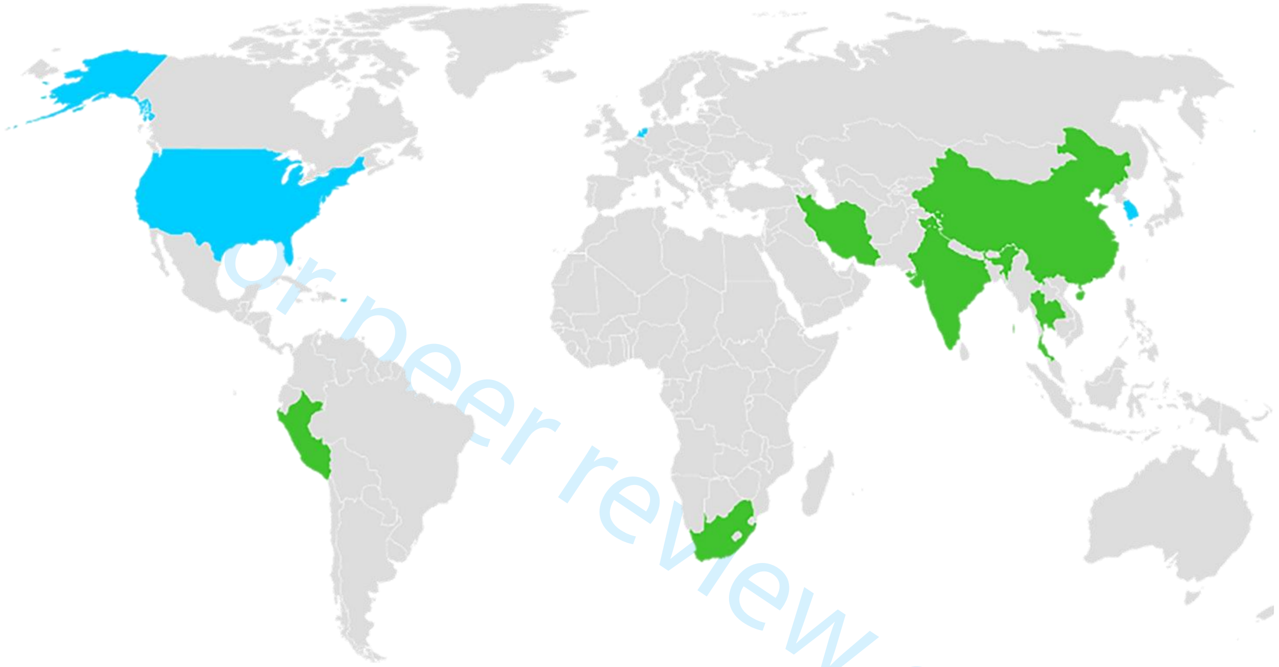

LMIC that developed and/or validated models included in this review (Green). Moreover, Asgari et al, Mogueo et al] and Saranburut et al validated risk models that were originally derivated in the Netherlands, South Korea and the United States, respectively (Blue).

For peer review only - http://bmjopen.bmj.com/site/about/guidelines.xhtml

S2 Figure: **Predictors included in the final models.**

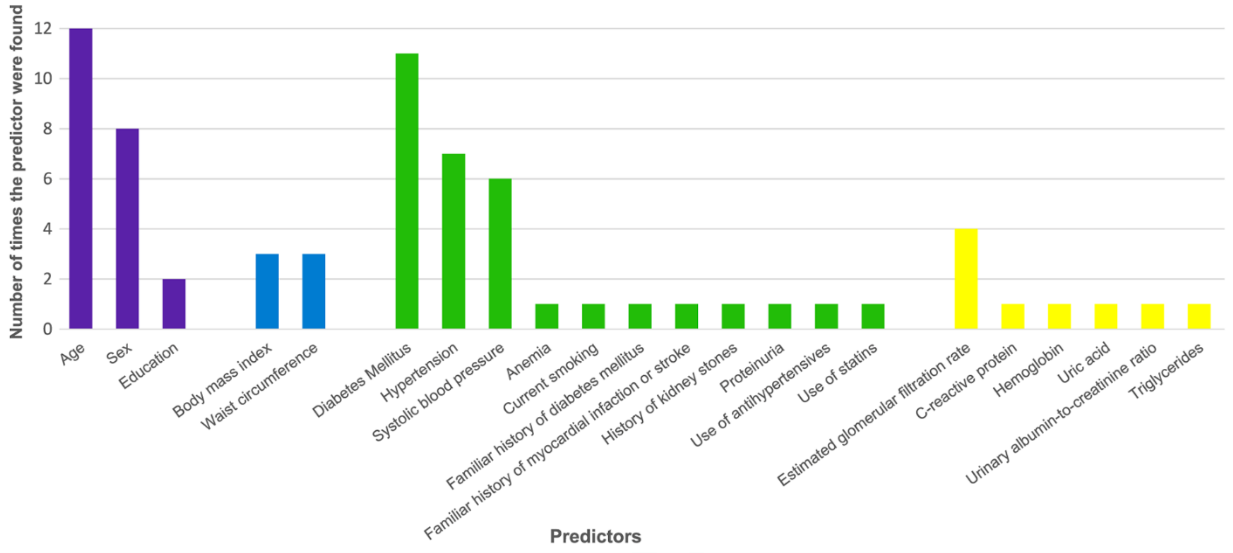

The colours of the bars identify the underlying characteristic of predictors inherent to: the subject (purple), anthropometrics (blue), clinical assessment and history (green), and laboratory measures (yellow).

For peer review only - http://bmjopen.bmj.com/site/about/guidelines.xhtml
